# Modulation of NKG2D, KIR2DL and Cytokine Production by *Pleurotus ostreatus* Glucan Enhances Natural Killer Cell Cytotoxicity Toward Cancer Cells

**DOI:** 10.3389/fcell.2019.00165

**Published:** 2019-08-13

**Authors:** Nehal M. EL-Deeb, Hala I. EL-Adawi, Abeer E. Abd EL-wahab, Ahmed M. Haddad, Hesham A. EL Enshasy, You-Wen He, Keith R. Davis

**Affiliations:** ^1^Biopharmaceutical Product Research Department, Genetic Engineering and Biotechnology Research Institute, City of Scientific Research and Technological Applications (SRTA-CITY), New Borg El-Arab City, Egypt; ^2^Department of Immunology, Duke University Medical Center, Durham, NC, United States; ^3^Department of Biology and Biotechnology Program, Indiana University, Bloomington, IN, United States; ^4^Medical Biotechnology Department, Genetic Engineering and Biotechnology Research Institute, City of Scientific Research and Technological Applications (SRTA-CITY), New Borg El-Arab City, Egypt; ^5^Environmental Biotechnology Department, Genetic Engineering and Biotechnology Research Institute, City of Scientific Research and Technological Applications (SRTA-CITY), New Borg El-Arab City, Egypt; ^6^Institute of Bioproduct Development, Universiti Teknologi Malaysia, Johor Bahru, Malaysia; ^7^Department of Bioprocess Engineering, Faculty of Chemical and Energy Engineering, Universiti Teknologi Malaysia, Johor Bahru, Malaysia

**Keywords:** NK cells, mushroom, *Pleurotus ostreatus*, breast cancer, lung cancer, polysaccharides, glucans

## Abstract

Medicinal mushrooms have been used for centuries against cancer and infectious diseases. These positive biological effects of mushrooms are due in part to the indirect action of stimulating immune cells. The objective of the current study is to investigate the possible immunomodulatory effects of mushroom polysaccharides on NK cells against different cancer cells. In this current study, fruiting bodies isolated from cultured *Pleurotus ostreatus* were extracted and partially purified using DEAE ion-exchange chromatography. The activation action of the collected fractions on Natural Killer cells was quantified against three different cancer cell lines in the presence or absence of human recombinant IL2 using three different activation and co-culture conditions. The possible modes of action of mushroom polysaccharides against cancer cells were evaluated at the cellular and molecular levels. Our results indicate that *P. ostreatus* polysaccharides induced NK-cells cytotoxic effects against lung and breast cancer cells with the largest effect being against breast cancer cells (81.2%). NK cells activation for cytokine secretion was associated with upregulation of KIR2DL genes while the cytotoxic activation effect of NK cells against cancer cells correlated with NKG2D upregulation and induction of IFNγ and NO production. These cytotoxic effects were enhanced in the presence of IL2. Analysis of the most active partially purified fraction indicates that it is predominantly composed of glucans. These results indicate bioactive 6-linked glucans present in *P. ostreatus* extracts activate NK-cell cytotoxicity via regulation of activation and induction of IFNγ and NO. These studies establish a positive role for bioactive *P. ostreatus* polysaccharides in NK-cells activation and induction of an innate immune response against breast and lung cancer cells.

## Introduction

Cancer is considered as one of the major human death causes worldwide. Recently, many of anti-cancer therapies are available, including chemo- and immune-therapeutic agents, which are known to give rise to various adverse effects. Therefore, it is needful to explore novel anti-tumor drugs with immunomodulatory effects avoiding any side effects. The immune response is a mechanism that break down and removes antigens from genetically non-identical organisms, foreign organisms and viruses, and cancer cells. The immune system relies on a variety of lymphocytes that serve specific functions in mediating immunity. Natural killer (NK) cells, unsensitized lymphocytes that found by Herberman et al. (1975) are not restricted by the MHC, but can target and damage a variety of cells via activation of innate immune system. NK cells have two roles pertinent to the natural immune response against pathogens and virally infected and neoplastic cells (Glas et al., 2000; Sepulveda and Puente, 2000). The first role is cytotoxicity that mediated by target cells (viral and bacterial infected cells) recognition and lysis. The second is the production of cytokine, mainly IFNγ, that can regulate both natural and specific immune responses. Mushrooms have gained much concerns lately due to their potential useful therapeutic effects (Balkwill, 2009; El Enshasy and Hatti-Kaul, 2013; Guggenheim et al., 2014). Many research articles are explained the positive role of natural products to activate both innate and adaptive immune system. Medicinal mushrooms have been considered as a novel therapeutic agent that may enhance cancer immunotherapy and patients’ survival. Mushrooms are known to produce antimicrobial, anti-inflammatory, cardiovascular-protective, antidiabetic, hepatoprotective, and anticancer agents (Nouroz et al., 2016). It is well-established that mushrooms are able to regulate the immune responses by affecting hematopoietic stem cells, lymphocytes, macrophages, T cells, dendritic cells (DCs), and NK cells (Ooi and Liu, 2000). Over the last 40 years, new research demonstrated the effective roles of mushrooms to reduce the growth of tumor cells, decrease tumoral angioneogenesis, and increase malignant-cell phagocytosis; these effects have been correlated with induced cytokine production (Guggenheim et al., 2014). Polysaccharides isolated from mushrooms significantly induce both innate and adaptive immune response and thus have inherent immune-stimulatory properties with varieties of clinical and medicinal applications (Ooi and Liu, 2000; Tsan and Gao, 2007). Several mushroom polysaccharides have been established as potent antitumor agents: Lentinan from *Lentinula edodes* (Chihara et al., 1970), SSG from *Sclerotinia sclerotiorum* FKL (Suzuki et al., 1988), and Schizophyllan from *Schizophyllum commne* Fries (Mitani et al., 1982; Daba and Ezeronye, 2003; Hong et al., 2012). These polysaccharides regulate both macrophages and T cells immunomodulatory chemokines and cytokines. Also, the D-Fraction polysaccharide Maitake that extracted from maitake mushroom (*Grifola frondosa* S.F. Gray) showed abilities to induce immune system activation by its effect on the macrophages, DCs, and NK cells (Kodama et al., 2003). To further explore this interesting finding, the current study focused on the immune- stimulatory effects of *Pleurotus ostreatus* polysaccharide fractions on NK cells and the role of cytokine secretion and stimulatory receptors in three NK-cancer cells co-culture models.

## Materials and Methods

### Mushroom Spawn Preparation

The used spawn was prepared in 250 ml bottles where sorghum grains were mixed with 5% (w/w) CaSO_4_ and soaked in water for 18 h. Then, all the excessed water was drained off and the bottles were filled to 3/4 with sorghum grains and sterilized by autoclave at 121°C for 20 min. The sorghum grains were inoculated with actively growing mycelium of *P. ostreatus* on PDA plates and incubated at 28°C for 12 to 15 days.

### Mushroom Cultivation

The *P. ostreatus* mushroom was cultivated using polythene bag method described by Bano and Srivastava (1962), with minor modifications. Dried rice straw was chopped into 5 to 7 cm length and soaked in water for 4 h in the presence of 5% (w/w) gypsum. The excess water was drained, and the substrate sterilized by autoclaving at 121°C for 20 min. About half kilogram of the substrate was placed in 40 × 60 cm polyethylene bags that were spawned with 10% mushroom mycelia grown on sorghum grains. This process was done in 3 layers each above 5 cm layer of the rice straw substrate. Subsequently, the resulted bags were placed into running room at 25°C ± 2°C under dark conditions. After spawn running process completion, the bags were placed into a humidified room at 22 ± 2°C and 80–90%. The bags were cut open on the sides without disturbing the beds and water sprayed twice daily to maintain moisture level. After 2 weeks ago, the fruiting bodies start to grow in 3 successive flushes, the complete flatten fruiting bodies were reaped, weighted and air dried at shading room at room temperature.

### Extraction of Polysaccharides

For exopolysaccharide (EPS) extraction, about 100 g of dried mushroom fruiting bodies was boiled in 1 L of distilled water for about 2 h in 3 w/v water, the protein fractions in the filtrate was precipitated by treating the clear supernatant with 10% trichloroacetic acid (1:1), and then centrifuged at 10,000 rpm at 2°C for 30 min. The collected supernatant was subjected to three successive three volume absolute alcohol extractions. At the end of extraction time; the EPS, were collected by centrifugation at 10,000 rpm at 2°C for 30 min. The obtained ethanol soluble EPS were recovered in a rotary evaporator at 40°C and stored at 4°C until the time of analysis. The extracted EPS were dialyzed against ddH2 over 5 days using dialysis membrane a having a 1000 Da MWCO (Thermo Fisher Scientific). The total protein concentrations of the samples were quantified by the Lowry method (Lowry et al., 1951).

### Partial Purification of the Extracted Polysaccharides Using DEAE Cellulose Column

About 0.3 g of the water-soluble polysaccharides were fractionated using DEAE ion exchange Colum chromatography (diethylaminoethyl cellulose backed column; 1.8 cm × 150 cm). After loading the extracts onto the column, fractions were eluted from the Colum using 100 ml of distilled water then with a NaCl salt solution gradient (0.5 to 1 mol/L). The resulted fractions were collected at constant rate (300 drop/min). The total Carbohydrate content was quantified by phenol-sulfuric acid method (Masuko et al., 2005) and the total protein concentrations was quantitatively measured by the Lowry method (Lowry et al., 1951).

### Viability Assays of the Crude Extracts With Its Semi-Purified Fractions on Peripheral Blood Mononuclear Cells (PBMCs) and NK Cells

#### Healthy Blood Donors

Peripheral blood from healthy donors were obtained from City of Scientific Research and Technological Applications and HLA allotypes for both cancer cells and blood samples established (Supplementary Table S1).

### Ethics Statement

This current study was implemented in City of Scientific research and technological applications (SRTA-City), center of excellency for drug preclinical studies (CE-DPS) Alexandria, Egypt and the protocols of blood sample collection were approved by the Research Ethical Committee at CE-DPS, SRTA-City, Alexandria, Egypt under international, national, and/or institutional guidelines. The blood samples were collected from healthy volunteers and all volunteers provided written informed consent in conformity with our all Declaration.

#### Isolation of PBMCs

PBMCs were recovered from the blood by gradient centrifugation method (Lohr et al., 1995), suspended in RPMI medium (2 × 10^5^ cells/ml) and 100 μl aliquots/well seeded into a rounded bottom 96 well plate.

#### Isolation of NK Cells

From whole blood of health volunteers, NK cells were isolated using Easy Sep human NK isolation Kit (Stem cells) according to the instruction protocols. About 5 × 10^7^ cells/ml were incubated in RPMI media with or without IL-2 in the presence of glutaraldehyde fixed monocytes cells as feeder cells with at a 1:2 ratio. Different concentrations of the extracted polysaccharides were incubated with the isolated PBMC and NK cells for 3 days at 37°C at 5% CO_2_. After that, the effects on viability of the polysaccharides were quantified using BioVision’s MTS Cell Proliferation Assay Kit. MTS assay is based on the quantification of a colored formazan product (soluble in cell culture media) that resulted from viable cells reductions of MTS tetrazolium compound.

### Quantification of the Induced Cytokines in LPS-IL-2-Induced PBMC Cells

About 2 × 10^5^ cells/ml of PBMCs were suspended in RPMI medium and 100 μl plated into a rounded bottom 96-well plate and cultured for about 24 h. After incubation, the inflammatory responses were induced with either 100 μl of LPS of *E. coli* (10 ng/ml) and IL-2, 500 U/ml for 24 h as a positive control or polysaccharides (5 mg/ml) After incubation, the levels of INFγ and TNF-α were quantitatively measured using Thermo Fisher Scientific^®^ Human INFγ and TNF-α ELISA Kit, according to the manufacturer’s instructions. Each experiment was repeated three times and the means and standard deviation (SD) calculated using GraphPad Prism 7.

### Cytotoxicity Assay

The anticancer activity of the activated NK cells combined with mushroom polysaccharides against HepG2, MCF7 and A549 cells were quantified using BioVision’s MTS Cell Proliferation Assay Kit.

#### NK Cells Activation

Natural Killer cells were recovered from health human blood cells and then cultured as described above. The non-toxic calculated doses of mushroom polysaccharides and its fractions were used separately to activate NK cells for 1, 3, and 5 days using the previously described incubation conditions.

#### Viability Assay

After activation, NK cells were collected with centrifugation at 2000 rpm for 10 min and finally, 3 times washed with prewarmed phosphate buffer saline solutions (PBS, pH:7.6). The activated NK cells were co-incubated with HepG2, MCF7 and A549 cells with ratios 1:1, 1:2 and 1:3 (starting with 2 × 10^5^ cells/ml NK cells), the cytotoxicity assay of the activated NK cells against cancer cells were quantified using BioVision’s MTS Cell Proliferation Assay Kit after 5 days.

### Enhancement the Anticancer Effects of Activated NK With IL-2 by Mushroom Fractions

The most effective mushroom fraction (Fraction 1; MU1) that showed the most potency in the activation of NK cells against cancer cells was selected for detailed studies. Initial experiments show that 5 mg/ml MU1 was not toxic to NK cells and that a 3-day activation time was optimal for cytolytic activity. These conditions were used for our detailed studies. About 6 × 10^5^ cells/ml NK cell were activated with MU1 in the presence of 2 ng/ml IL-2. After NK activation, the activated NK cells were recovered with centrifugation at 2000 rpm, then washed twice with prewarmed PBS. The most sensitive cancer cell lines (MCF7 and A459 cells) at concentrations 2 × 10^5^ cells/ml co-incubated with the recovered pre-activated NK cells. The anticancer activity evaluated after 5 days of culture using BioVision’s MTS Cell Proliferation Assay Kit as descried above, the cytotoxicity results were visualized confirmed using trypan blue exclusion assay. Each experiment was three times repeated and the means and SD calculated with GraphPad Prism 7.

### NK Lytic Activities Against MCF7-EGFP

MCF7 cells that express green fluorescent protein (MCF7-EGFP) were kindly provided from faculty of pharmacy, Egypt, in which GFP considered as a tumor-specific marker. In order to confirm the cytotoxic effects on the targeted MCF7-EGFP cells in the presence of IL-2 upon the direct contact with Mu1 activated NK cells, GFP signal intensity was quantified by flowcytometry. As described above, MCF7-EGFP cells were co-incubated with NK cells activated with MU1 (5 mg/ml) with or without IL-2 for 3 days, NK cells activated with CD3 and CD28 was used as positive stimulator control (1 μg/ml) in the positive control sample. MCF7-EGFP were collected in FACS staining buffer and analyzed by flowcytometry to quantify the GFP levels comparing with the negative and positive control samples.

### Quantification of the Induced INFγ, and TNF-α in NK-Cancer Cell Models by Mushroom Extract

MCF7 and A459 cells were co-incubated with NK cells activated with or without MU1 (5 mg/ml) for 3 days in the presence of IL-2. After treatment, the intracellular induced INFγ, and TNF-α were checked by intercellular flowcytometry staining kit (biolegend) in the presence of protein transport inhibitor and CD3 and CD28 as positive stimulator control (1 μg/ml). For flowcytometry, intracellular staining was performed in FACs staining buffer using antibodies against INFγ (PE biolegend) and TNF-α (APC-biolegend).

Also, NK-cancer cells were co-cultured as described above but without protein transport inhibitor, the extracellular induced INFγ, and TNF-α in culture filtrates were quantified using INFγ, and TNF-α ELISA kit (Biolegend) according to the manufacturer’s.

### Quantification of the Activation Markers of NK Cells in NK-Cancer Cell Models Using RTqPCR

MCF7 and A459 cells were co-incubated with NK cells activated with MU1 (5 mg/ml) with or without IL-2 for 3 days. After treatment, NK cells total RNA was extracted using Thermo Fisher Scientific MagJET RNA Kit, according to the manufacturer’s protocol, and genomic DNA was eliminated using a DNase (QIAGEN). RNA quality was confirmed by the A260/A280 ratio using a NanoDrop (Thermal Scientific) spectrophotometer. Aliquots of 10 ng RNA from each sample were used for subsequent cDNA synthesis. The total cDNA was synthesized using Thermo Fisher Scientific cDNA Synthesis Kit based on the manufacturer’s protocol and PCR machine (biorad). cDNA samples were used to evaluate the expression of INFγ, NKG2D and KIR2DI genes using Maxima SYBR Green/ROX qPCR Master Mix using forward and reverse primer (Supplementary Table S2), SYBR green PCR master mix, according to the manufactured protocol and qPCR machine (Real-Time PCR Detection System Bio-Rad, CFX96 Touch Deep Well). The thermal cycling protocol of RT was as follows: 50°C for 2 min, 95°C for 15 min 40 cycles of 15 s at 94°C, 30 s at 50°C and 30 s at 72°C. RT-PCR were analyzed, and gene expression figures were generated by CFX96 Touch Deep Well, Real-Time PCR Detection System Bio-Rad) software.

### Nitric Oxide (NO) Measurements in Induced NK Cells

NK cells were activated with MU1 (5 mg/ml) with 2 ng/ml IL-2 for 5 days or IL2 alone as control group. The pre-activated NK cells were incubated with the target cells (MCF7 or A459 cells) with a 3:1 ratio for 24 h; the induced NO concentrations were quantified using Nitric Oxide Colorimetric Assay Kit (Biovision, United States). Both LPS and INFγ at final concentrations 10 ng/ml was used to induce NK-NO in a positive control group.

### Cell Cycle Analysis of the Treated MCF7 and A459 Cells Using Flow Cytometry

Using flow cytometry and a propidium iodide (PI) labeling protocol, the alterations in cell cycle pattern in response to the activated NK cells were determined according to Leonce et al. (2001). In this assay, PI labeling is used to differentiate between living cells and dead cells and the cell cycle analysis is done based on the stoichiometric binding of PI to intracellular DNA. At the end of treatment with the activated NK cells, cancer cells were 3 times washed with PBS and then collected by trypsinization. The obtained cells (about 2 × 10^5^ cells/ml) were then re-suspended in warm PBS, fixed in about 4 ml ice cold ethanol and stained for 30 min with 0.5 mL warm PI solution (each 7 ml of PI solution has 6 ml of PBS, 0.35 ml of 0.1% PI solution, 0.7 ml RNase A solution (1 mg/ml). The samples were store on 4°C until the analysis by flowcytometry. For a positive control group, A mixture of LPS and INFγ (final concentrations of 10 ng/ml) was used. Each experiment was repeated three times and the means and SD were calculated with GraphPad Prism 7.

### Analysis of the Most Potent Polysaccharides Fraction

The most potent fraction (Mu1) was selected for further purification and composition analysis.

#### Size-Exclusion Chromatography

Size-exclusion chromatographic analysis was implemented using an Agilent 1200 HPLC system equipped with a Superose 6 (GE) column. A 1mg/ml solution of the sample was prepared and centrifuged to remove any insoluble portion. A single 100 μl injection of the dialyzed samples was made into the HPLC and the sample eluted using ammonium acetate (50 mM) at 1.0 ml/m flow rate of eluting compounds were monitored using a RI detector.

#### Glycosyl Composition

Glycosyl composition analysis was accomplished by combined gas chromatography/mass spectrometry (GC/MS) of the per-O-trimethylsilyl (TMS) derivatives of the monosaccharide methyl glycosides resulted from acidic methanolysis of the sample as reported by Santander et al. (2013). Briefly, 300 μg of semi-purified mushroom fraction 1 was heated for 17 h at 80°C with methanolic HCl in a sealed screw-top glass test tube. After cooling and solvent removal under nitrogen steam, the sample was mixed with a methanol, pyridine, and acetic anhydride mixture for 30 min. After that, the solvents were evaporated, and the sample derivatization was performed with Tri-Sil^®^ (Pierce) at 80°C for 30 min. GC/MS analysis of TMS methyl glycosides was carried out using an Agilent 7890A GC interfaced to a 5975C MSD, using a Supelco Equity-1 fused silica capillary column (30 m × 0.25 mm ID).

#### Glycosyl Linkage Analysis

The glycosyl linkage analysis was performed as following: the semi-purified mushroom fraction 1 were permethylated, reduced, re-permethylated, depolymerized, reduced, and acetylated. Then, the consequent partially methylated alditol acetates (PMAAs) analyzed by gas chromatography-mass spectrometry (GC-MS) as illustrated by York et al. (1986). 1 mg of mu1 was suspended in 200 μl of dimethyl sulfoxide, and then stirred for 2 days and then the resulted samples was permethylated using potassium dimsyl anion and iodomethane. The reduction process of permethylated uronic acids were performed using lithium borodeuteride. Then, the formed products was permethylated secondly by the method of Anumula and Taylor (1992) to confirm the polymer complete methylation. After that, the permethylated sample was hydrolyzed in sealed tube by 2 M trifluoroacetic acid for 2 h at 121°C), reduced with NaBD4, and then mixed with acetic anhydride/TFA to perform the acetylation process. The obtained PMAAs were tested using an Agilent 7890A GC interfaced to a 5975C MSD (mass selective detector, electron impact ionization mode); separated on Supelco 2331 fused silica capillary column (30 m × 0.25 mm ID).

### Statistical Analyses

Each experiment was confirmed three times and data are introduced as experimental result mean ± SEM. Between groups comparisons were estimated by a one-way analysis of variance with multiple comparisons, and two way ANOVA with Turkey’s test. The correlation analysis was performed by GraphPad 7.0.

## Results

### Partial Purification of Mushroom Crude Extract by DEAE Cellulose Anion-Exchange Chromatography

**FIGURE 1:**
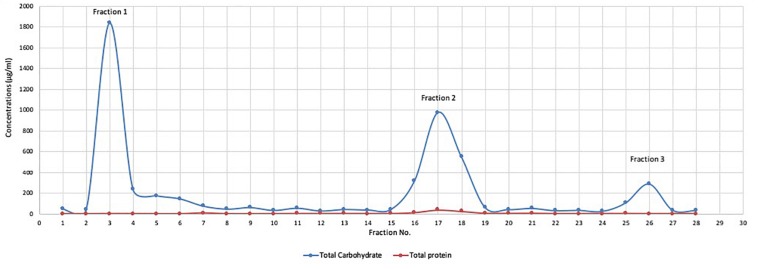
The total protein and total carbohydrate of DEAE cellulose column fractions. Polysaccharide fractions were eluted from DEAE cellulose column with 100 ml of distilled water and NaCl salt solution gradient (0.5 to 1 mol/L) grading at constant rate (300 drop/min). Carbohydrate content was determined by phenol-sulfuric acid method and the protein concentrations in the samples was quantified by the Lowry method. The results indicated the presence of 3 major beaks with low protein content. The maximum carbohydrate concentrations were recorded in beak 1 with concentration (1.8 mg/ml) followed by beak 2 (0.97 mg/ml) and the lowest concentration recorded in beak 3 (0.28 mg/ml).

The collected mushroom fruiting bodies were homogenized (100 g of mushroom fruiting bodies in 300 ml distilled water), and polysaccharides extracted by three volumes absolute ethanol. The crude extract was subjected to total protein quantification and the results demonstrated that the concentrations of total protein in crude extracts ranged from 1.1 to 1.6 mg/ml in six separate extractions. The crude extract was subjected to partial purification using a DEAE cellulose column and three distinct fractions (Mu1, Mu2, and Mu3) were identified according to total carbohydrate content assay. The maximum total carbohydrate concentrations (1.83 mg/ml) were recorded in Fraction 1(Mu1) followed by Fraction 2 (Mu2, 0.975 mg/ml) and Fraction 3 (Mu3, 0.28 mg/ml). After purification, the protein concentrations in the three fractions were very low and ranged from 0 to 38.18 μg//ml; the maximum concentration was 38.18 μg/ml in Mu2 (Figure 1).

### Viability Assays of the Crude and Purified Mushroom Fractions on PBMC and NK Cells

**FIGURE 2:**
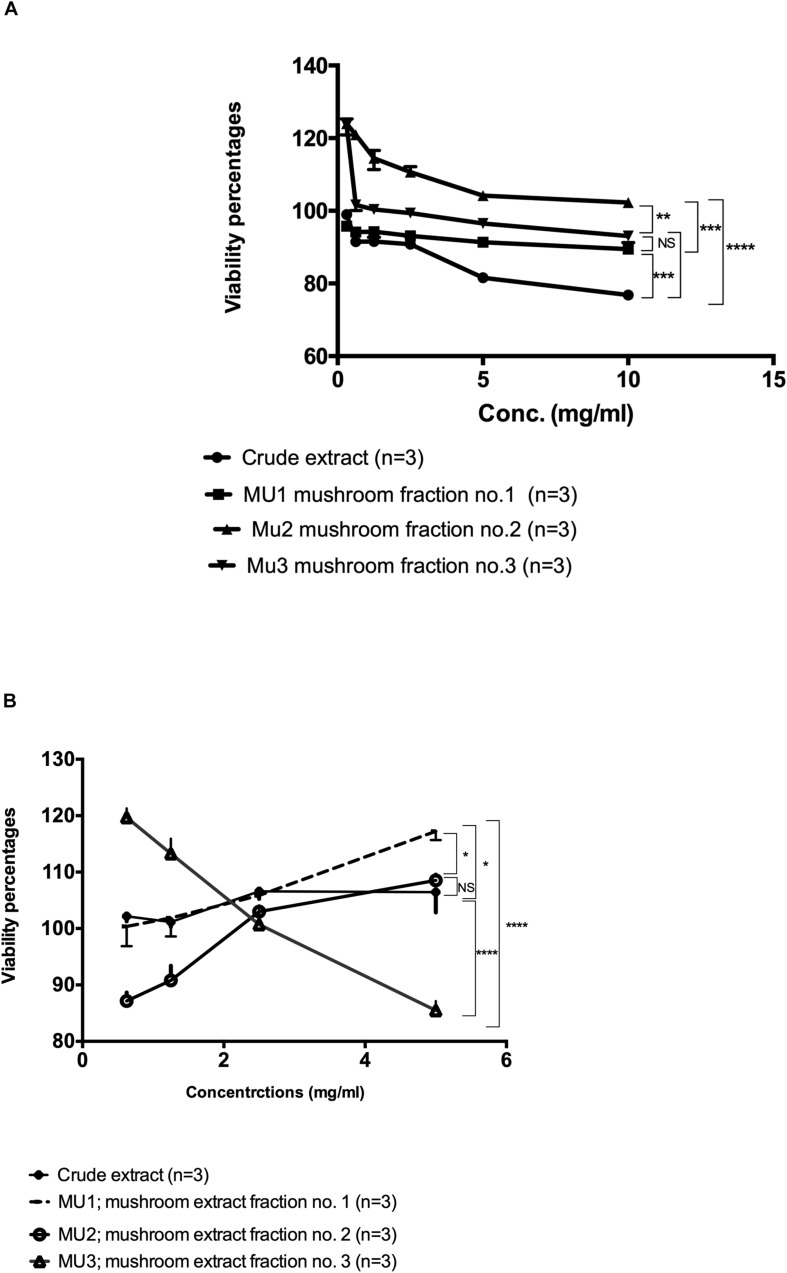
Viability assay of the Mu extract with its fractions on PBMC and NK cells. **(A)** The viability assay test of the semi-purified mushroom fractions 1, 2, and 3 (Mu1, Mu2, and Mu3) with its crude extract on PBMCs using MTS Cell Proliferation Assay. All three Fractions showed abelites to induce cellular proliferations up to 120%. Data are mean and SD, ^****^p < 0.0002, ^∗∗∗^*p* < 0.001, ^∗∗^*p* < 0.01, ^*^p < 0.0370 and NS *p* = 0.423 with two way ANOVA with Turkey’s test, **(B)** The viability assay test of the semi-purified mushroom fractions 1, 2, and 3 (Mu1, Mu2, and Mu3) with its crude extract on PBMCs using MTS Cell Proliferation Assay, NK cells proliferations increased by using Mu1 and Mu2. Data are mean and SD, ^****^p < 0.0001, ^*^p < 0.08 and NS *p* = 0.6025 with two-way ANOVA with Turkey’s test.

In order to compare the potential viability effects of the crude mushroom extract with the purified DEAE-purified fractions, cytotoxicity assays on PBMC and NK cells were performed using MTS assay protocol. On PBMC (Figure 2A), all purified fractions did not show any major toxic effects on PBMC cells and some fractions showed ability to induce cellular proliferation especially for Fraction 2 and 3 (Mu2, Mu3) with cellular growth up to 120% of the control. While, the maximum toxic effects of the crude extract were a 23.4% reduction in viability (Figure 2A). On the other hand, on NK cells (Figure 2B), the all extracts didn’t show any toxic effects at higher concentrations (5 mg/ml). By treating cells with crude, Mu1 and Mu2 extracts at 5 mg/ml, the cellular proliferation increased, while on Mu3 treated NK cells this increased growth was noted at 2.5 mg/ml. The maximum cytotoxicity percentage (14.5%) was detected in NK cells treated with 5mg/ml Mu3. The obtained results of this experiment confirm our success to develop NK cells expansion without any viable mixed cells or genetically modified cell.

### Regulation of the Induced TNFα and INFγ Using Mushroom Polysaccharides

**FIGURE 3:**
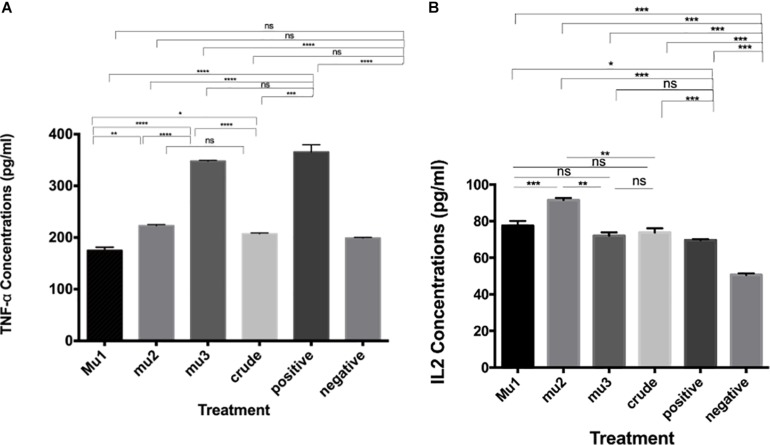
Induction of TNFα and INFγ by mushroom polysaccharides in PBMC cells. ELISA assays to quantify the levels of TNF-α **(A)** and IL-2 **(B)** induced by the crude extract and the mushroom fractions in PBMC cells; Mu1, Mu2 and Mu3 (5 mg/ml) for 24 h. All groups were compared with the positive control (induced with 100 μl of *E. coli* LPS and INFγ, 10 ng/ml). Data are mean and SD, ^****^p < 0.0002, ^∗∗∗^*p* < 0.001, ^∗∗^*p* < 0.01, ^*^p < 0.030 and ns *p* = 0.332 with one-way ANOVA with multiple comparisons (*n* = 2).

The effects of mushroom extract and purified fractions on cytokines (TNF-α and INFγ) induction of PBMC were measured using ELISA assay and compared with the positive induced cells (LPS- IL-2-PBMCs induced cells). The results indicate that, comparing with the positive control (211.18 pg/ml), crude extract (82.62 pg/ml), Mu1 (191.74 pg/ml), Mu2 (62.31 pg/ml) and Mu3 (69.08 pg/ml) showed activities to induce TNF-α in PBMC with vantage to Mu1 and without significant differences between Mu2 and Mu3 (Figure 3). Furthermore, all treatments showed abilities to slightly induce INFγ comparing with the positive induced PBMC (36.78 pg/ml), Mu1 (35.46 pg/ml) showed abilities to induce INFγ without significant differences with the positive control cells. While, crude extract induced INFγ with value 25.69 pg/ml followed by Mu2 (14.06 pg/ml) and Mu3 (13.21 pg/ml) without significant differences between them.

### Anticancer Activates of the Activated NK Cells

**FIGURE 4:**
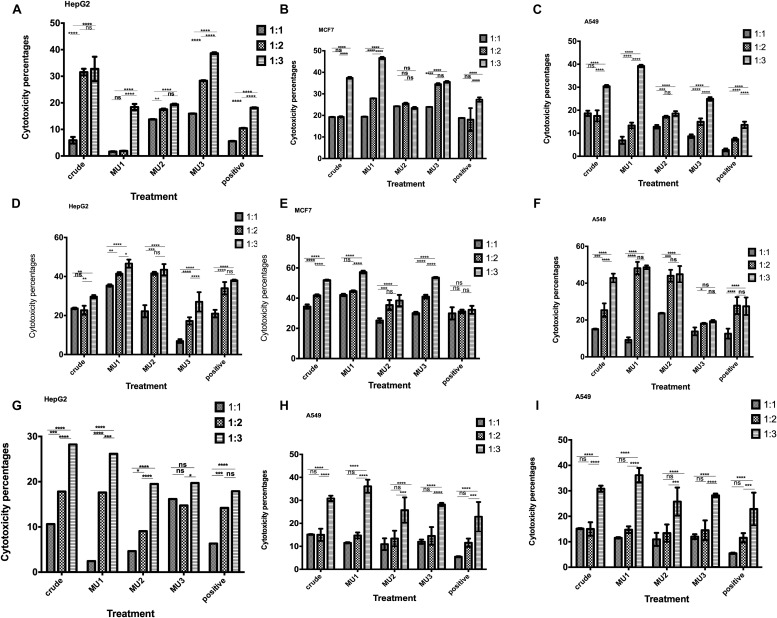
Anticancer activity of the Mushroom extracts activated NK cells against cancer cells. Cytotoxic activity of 1 day activated NK cells with crude mushroom extracts and its fractions against HepG2 **(A)**, MCF7 **(B)** and A549 **(C)** cells after 5 days incubation was quantified using MTS assay, comparing with the positive control (induced with 100 μl of *E. coli* LPS and INFγ, 10 ng/ml). The cytotoxic effects of 3 days activated NK cells with crude mushroom extracts and its fractions against HepG2 **(D)**, MCF7 **(E)** and A549 **(F)** cells after 5 days incubation also quantified as previously. Also, 5 days activated NK cells cytotoxic effects was quantified against HepG2 **(G)**, MCF7 **(H)** and A549 **(I)** cells after 5 days incubation was quantified using MTS assay. Panels **(A,D,G)** represent activated NK cells with polysaccharides for 1,2 and 3 days, respectively and co-incubated with HepG2 cells for 5 days. Panels **(B,E,H)** represent activated NK cells with polysaccharides for 1,2 and 3 days, respectively and co-incubated with A549 cells for 5 days. Panels **(C,F,I)** represent activated NK cells with polysaccharides for 1, 2 and 3 days, respectively and co-incubated with MCF7 cells for 5 days. NK cells were incubated with target cancer cells with ratio, 1:1, 1:2 and 1:3. Data are mean and SD, ^****^p < 0.0002, ^∗∗∗^*p* < 0.001, ^∗∗^*p* < 0.0125, ^*^p < 0.0370 and ns *p* = 0.4007 with two way ANOVA with Turkey’s test (*n* = 3).

**FIGURE 5:**
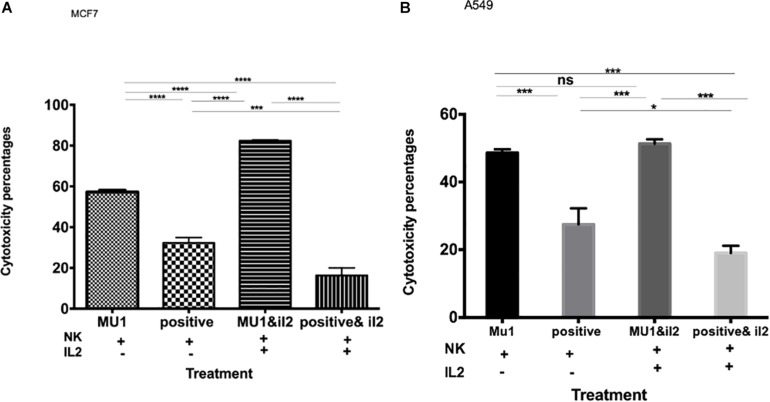
The effect of IL2 on the anticancer activity of mushroom activated NK cells. The effect of pre-activated NK cells with mushroom fraction No. 1 with or without IL2 against MCF7 **(A)** and A549 **(B)** cells, comparing with the positive control (induced with 100 μl of *E. coli* LPS and INFγ, 10 ng/ml). Data are mean and SD, ^****^p < 0.0001, ^∗∗∗^*p* < 0.001, ^*^p < 0.037 and ns *p* = 0.342 with one-way ANOVA with multiple comparisons (*n* = 3).

The anticancer effects of NK cells pre-activated with mushroom extracts and its fractions were tested against, HepG2, MCF7 and A549 cells. Among the different activation times and coincubation periods, the most significant anticancer effects were recorded after 3 days of NK cells activation and followed by 5 days incubation with cancer cells at ratio 1:3 target to effector cells (Figures 4D–F) comparing with 1 day activation (Figures 4A–C). By increasing the activation incubation to 5 days, the anticancer activity decreased (Figures 4G–I). Among the tested treatments, Mu1 is the most potent NK activator treatment against all cancer cells with highest activity against MCF7 (57.2%, Figure 5B) cells at a ratio of 1 target cells: 3 activated NK cells compared with 42.0% inhibition using 1:1 cell ratio, and 44.6% at 1:2 ratio. Also, by activating NK cells with Mu1 for 3 days and incubating with HepG2 cells at a ratio 1:3 for 5 days, the inhibition increased from 17.7% (after one day activation Figure 4A) to 46.5% (Figure 4D). In NK cells activated for 3 days with Mu1 and incubated with A549 cells at a ratio 1:3, the inhibition increased from 39.22% after one day activation, Figure 4C) to 48.6% at 6 days incubation (Figure 4I).

The anticancer effect of Mu1 activated NK cells (activated for 3 days and co-incubated with cancer cells for 5 days at a 1:3 ratio with either MCF7 or A549 were evaluated in with or without IL-2. The data in Figures 5A,B show significantly increased anticancer effects in the presence of IL-2, on MCF7 the anticancer effects increased from 57.23 to 81.2% (Figure 5A) and trended upward from 48.6 to 51.30% on A549 cells (Figure 5B).

To confirm these enhanced cytotoxicity results, trypan blue exclusion assay was used. The results indicated a significant enhancement in the anticancer effects of Mu1fraction in the presence of IL-2 on MCF7; the anticancer effects increased from 60.6 to 85.2% and trended upward from 54.1 to 62.6% on A549 cells (Supplementary Figure S1).

### Cytolytic Effects of Mu1 Activated NK Cells on MCF7-EGFP

**FIGURE 6:**
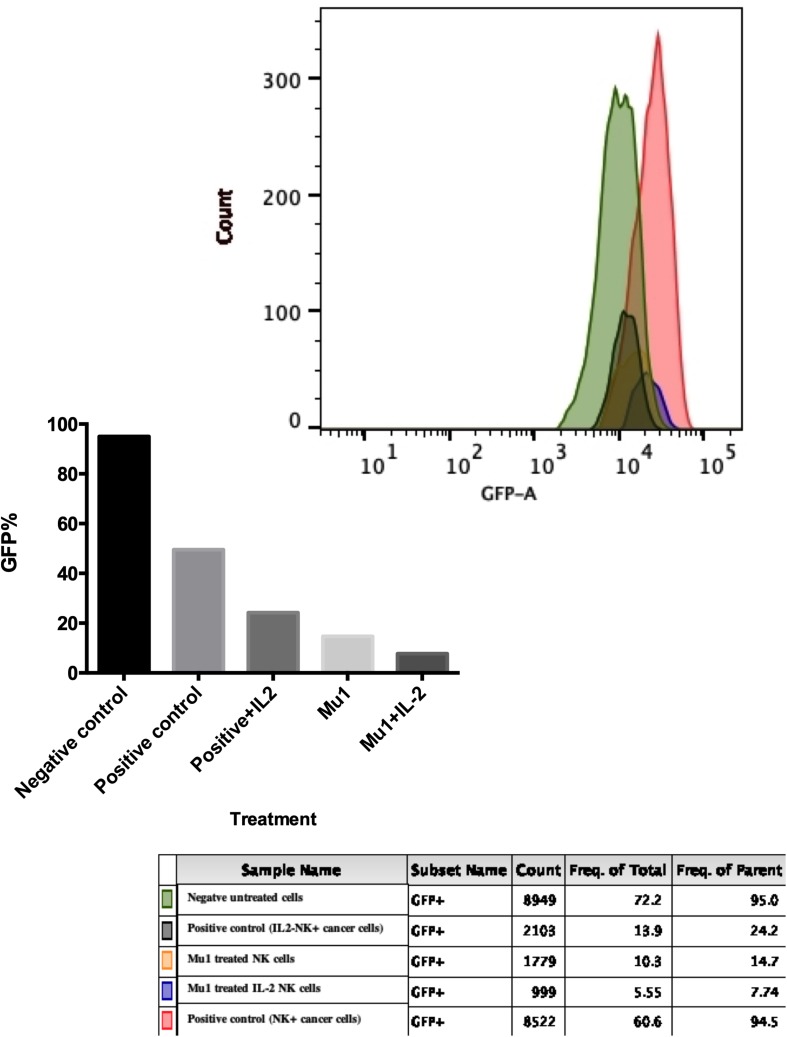
Cytotoxic effects of Mu1 activated NK cells on EGFP-MCF7 cells. Flowcytometric analysis of GFP emission after EGFP-MCF7 treatment with NK cells activated with mushroom fraction 1(Mu1) in the presence or absence of IL-2. The upper panel represent the flowcytometry histogram of the negative untreated cells (green color), positive control cells (red one), positive control with IL-2 (deep green), MU1 activated NK cells (orange) and MU1 activated NK cells with IL-2 (blue color). The lower panel represent the GFP% frequency of parent populations that showed very low GFP% (7.74) of MCF7 cells treated with Mu1 in the presence of IL-2.

The cytotoxic effects of mu1-activated NK cells against MCF7-EGFP cells was confirmed using flowcytometry as shown in Figure 6. A dramatically decrease in GFP emission was quantified in MCF7 cells treated with Mu1-IL-2 activated NK cells (7.74%) with significant differences between it and Mu1-NK cells (14.7%) and positive control (24.2%). It is obvious that the presence of IL-2 greatly enhanced the cytolytic effects of NK cells either in the presence or absence of Mu1.

### Quantification of Cytokines Production of NK Cells in NK-Cancer Cells Model Using ELISA and Flowcytometry

**FIGURE 7:**
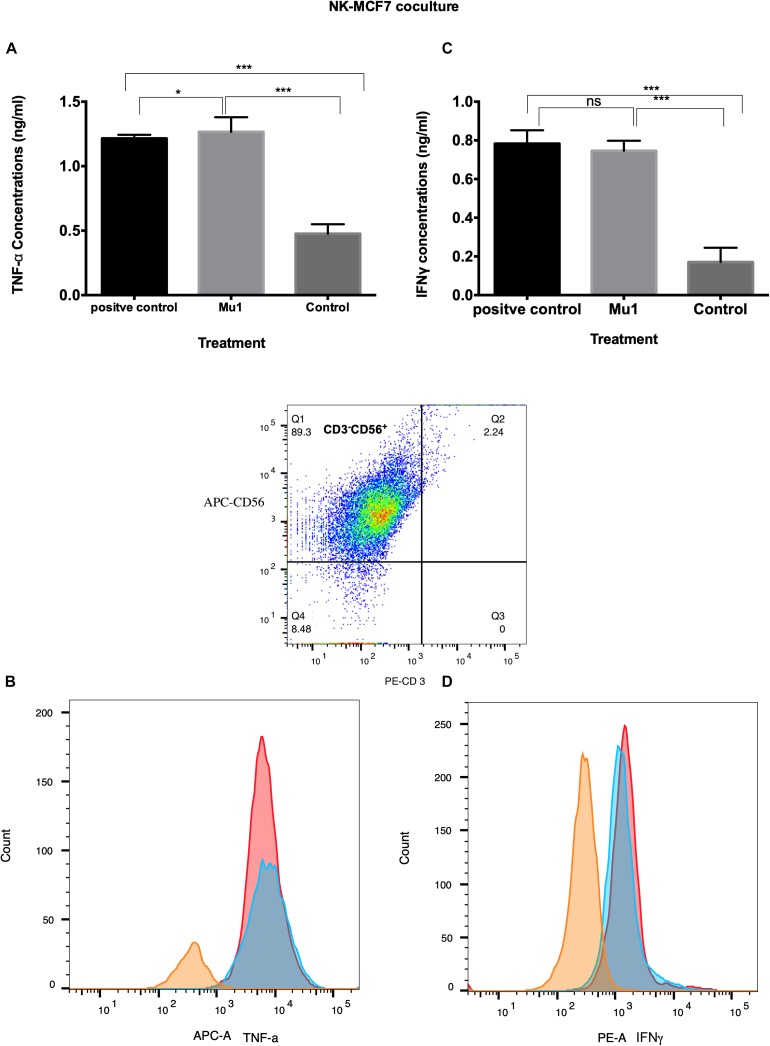
Detection of the induced TNF-α and INFγ in NK-MCF7 co-culture using ELISA and flowcytometry. Purified NK cells were first cultured for 3 days in the presence or absence of mushroom fractions mu1 and IL-2, NK cells from each group were then co-culture with MCF7 cells (ratio of 3:1, NK cells: cancer cells) or with CD3CD28 at final 10 ng/ml (as positive control), each for 3 days. Data are mean and SD, ^****^ *p* < 0.0001, ^∗∗∗^ *p* < 0.001, ^*^ *p* < 0.037 and ns *p* = 0.342 with one-way ANOVA with multiple comparisons (*n* = 3). The panels **(A)** and **(C)** represent the induced TNF-α and INFγ quantified by ELISA indicating enhancement in cytokines production after cancer cell treatment with NK cells activated with Mu1 in the presence of IL-2. Panels **(B)** and **(D)**; by gating on CD3^–^CD56^+^ cell populations, the flowcytometric histogram of cytokine production indicate an increase in its production after Mu1 + IL-2 activated NK cells treatment (red histogram) comparing with the positive control cells (blue histograms) and the negative one (orange histogram).

In MCF7 co-culture model, Mu1 treated NK cells showed abilities to induce both TNF-α and INFγ in MCF7 co-culture (Figure 7), the ELISA quantification results indicated that TNF-α increased to 1.27 from 0.47 ng/ml of the control group comparing with 1.21 ng/ml in the positive group (Figure 7C). Also, an increased in INFγ concentration from 0.17 ng/ml to 0.74 was quantified in mu1 treated Mu1 and 0.783 in CD3CD28 positive control (Figure 7A). Also, the flowcytometry results indicated increased TNF-α and INFγ similar to that observed in the CD3CD28 positive control group (Figures 7B,D).

**FIGURE 8:**
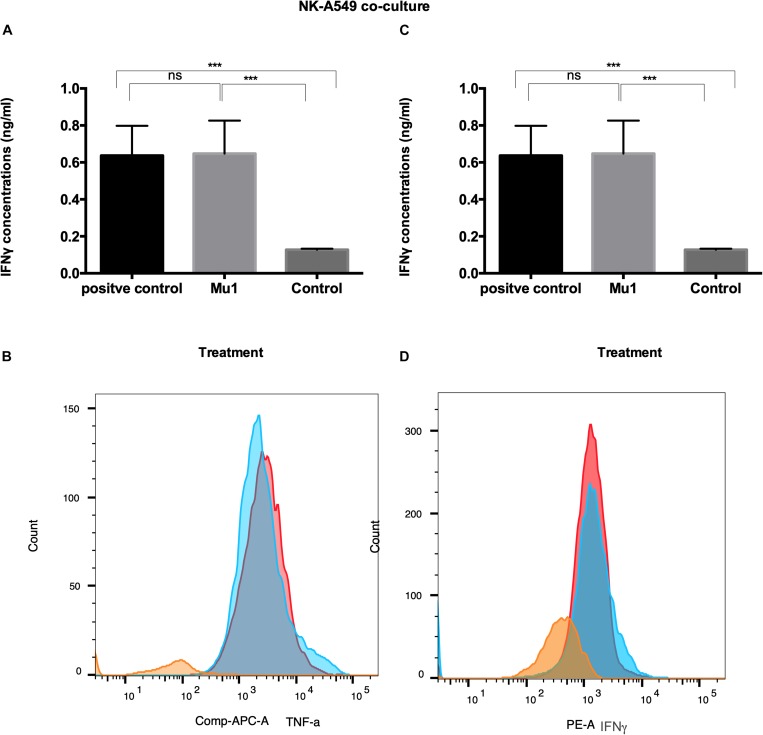
Detection of the induced TNF-α and INFγ in NK-A549 co-culture using ELISA and flowcytometry. Purified NK cells were first cultured for 3 days in the presence or absence of mushroom fractions mu1 and IL-2, NK cells from each group were then co-culture with A549 cells (ratio of 3:1, NK cells: cancer cells) or with CD3CD28 at final 10 ng/ml (as positive control), each for 3 days. Data are mean and SD, ^****^ *p* < 0.0001, ^∗∗∗^ *p* < 0.001, ^*^ *p* < 0.037 and ns *p* = 0.342 with one-way ANOVA with multiple comparisons (*n* = 3). The panels **(A)** and **(C)** represent the induced TNF-α and INFγ quantified by ELISA indicating enhancement in cytokines production after cancer cell treatment with NK cells activated with Mu1 in the presence of IL-2. Panels **(B)** and **(D)**; by gating on CD3^–^CD56^+^ cell populations, the flowcytometric histogram of cytokine production indicate an increase in its production after Mu1 + IL-2 activated NK cells treatment (red histogram) comparing with the positive control cells (blue histograms) and the negative one (orange histogram).

Also, in NK-A549 co-culture, Mu1 treated NK cells enhance both TNF-α and INFγ induction. The ELISA results demonstrated increase in TNF-α concentrations to 1.02 from 0.46 ng/ml of the control group compared to 1.24 ng/ml in the positive control group (Figure 8A). Also, an increased in INFγ concentration was recorded from 0.13 to 0.64 ng/ml in Mu1 treated cells versus 0.63 in the CD3CD28 positive control (Figure 8C).

Also, the flowcytometry results (Figures 8B,D) indicated an increase in TNF-α and INFγ levels comparing to the CD3CD28 positive control group.

### Quantification of the Activation Markers of NK Cells in NK-Cancer Cells Model Using RTqPCR

**FIGURE 9:**
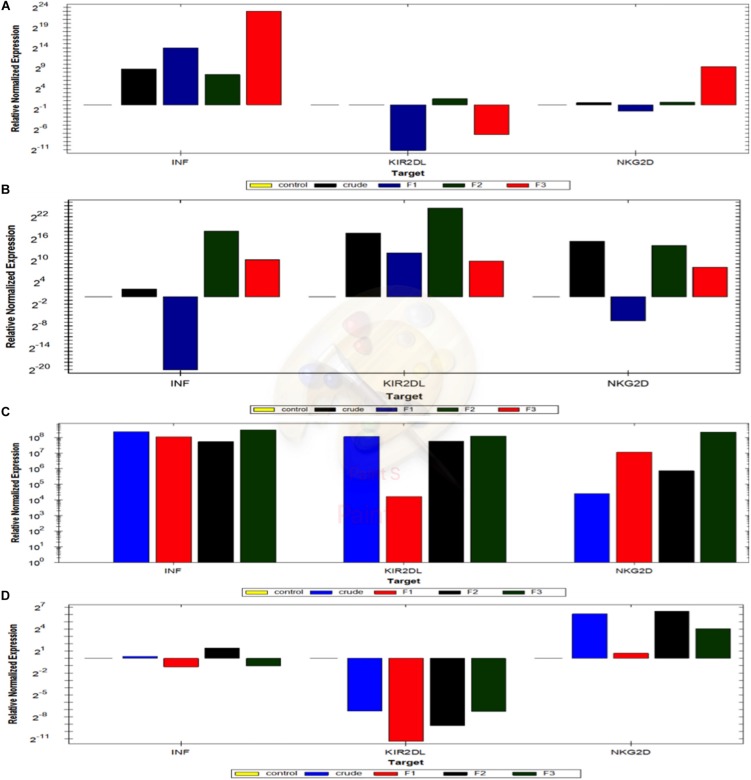
Quantification of the activation markers of NK cells in NK-Cancer cells model using RTqPCR. The expressing levels of INF gamma, KIR2Dl and NKG2D genes of NK cells reactivated with crude extract, mushroom fraction no. 1 (F1), mushroom fraction no. 2 (F2), mushroom fraction no. 3 (F3) for 3 days and co-incubate with target cancer cells MCF7 **(D)** and A549 cells **(C)** for 3 days with ratio 1:3 (target: effector cells) in the absence of IL2 and with MCF7 **(B)** and A549 cells **(A)** in the presence of IL2. All results were normalized using β actin gene expression and relative to the positive control cells (target cells with NK cells). All figures above the control base line represent the upregulation folding and that below the control base line represent downregulation folding.

The activated markers of MU pre-treated NK cells in co-culture models with cancer cells in the presence or absence of Il2 were quantified using qRT-PCR to detect the expression levels of INFγ, NKG2D and KIR2D genes. The expression pattern of these genes is significantly differed among the tested cell lines using NK cells with or without IL2 (Supplementary Tables S3–S6 and Figure 9).

#### NK-MCF7 Co-culture Model

INFγ expression levels in NK cells did not change from the control group upon using the crude extract or with the partially purified fractions (Supplementary Table S2). While, by using 2ng/ml IL-2, NK cells pre-activated with Crude, Mu2 and Mu3 showed upregulation in INFγ expression levels compared to that observed using to Mu2 (Supplementary Tables S4, S6 and Figures 9B,C). Also, IL-2addition to NK cells is greatly up-regulated the KIR2DL expression in NK-MCF7 co-culture model comparing to the NK co-culture group without IL-2, that showed downregulation pattern in KIR2DL gene among all trials of pre-activated models. While, a slight change was observed in NKG2D gene expression level upon IL-2 usage, IL-2 showed a slightly enhancement in NKG2D gene expression in F3-NK pre-activated cells (Supplementary Table S4 and Figures 9B,C).

#### NK-A549 Co-culture Model

The addition of IL-2 to NK in NK-A549 co-culture model didn’t recorded any significant enhancement in the expression levels of the all used genes (Supplementary Table S5 and Figures 9A,D) moreover, its addition reduced the upregulation effects of mushroom extracts on both NKG2D and KIR2DL genes in mushroom extract pre-activated NK cell groups. INFγ upregulation levels of NK cells that pre-activated with Mu1, Mu2 and Mu3 didn’t significantly changed after the addition of IL-2 while, IL-2 enhanced INFγ expression in NK cells pre-activated with crude extract. Also, IL-2 addition counteracted the upregulated expression of KIR2DL in NK cells to cause down-regulated expression in all pre-activated models. The same was also seen in NKG2D expression; IL-2 addition reduce the upregulation pattern in mushroom extract NK-pre-activated models to cause expression levels similar to the control non-activated NK group (Supplementary Table S5 and Figures 9A,D).

### Induction of Nitric Oxide (NO) in NK-Cancer Cells Co-culture Model

**FIGURE 10:**
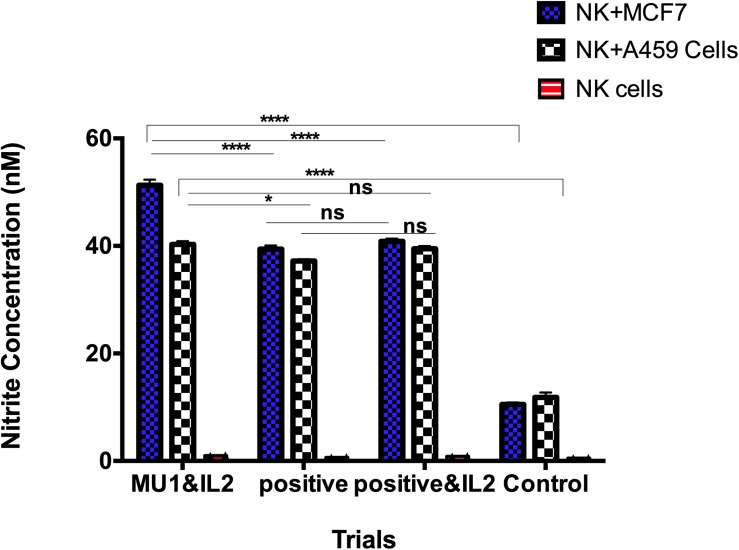
NO production by human NK cells in NK-cancer cells model. NO production by human NK cells was assessed by the Griess reaction using nitrate reductase. Purified NK cells were first cultured for 3 days in the presence or absence of mushroom fractions mu1 and IL-2, NK cells from each group were then co-culture with either MCF7 or A549 cells (ratio of 3:1, NK cells: cancer cells) or with LPS& INFγ at final 10 ng/ml (as positive control), each for 24 h. NO could not be detected above baseline in supernatants of NK cells cultured alone for 24 h. NO production was observed as nitrite/nitrate in the supernatants of NK cells stimulated by co-culture with cancer cells (target cells). Data are mean and SD, ^****^p < 0.0001, ^*^p < 0.0170 and ns *p* = 0.7869 with two way ANOVA with Turkey’s test (*n* = 3).

The accumulation of the induced nitrite/nitrate was detected in culture filtrate of human NK cells after each of treatments (Figure 10). These included cells with IL2 addition and omission and/or mushroom fractions for 5 days. The positive control group was designed as previously with adding LPS and INFγ in co-culture with either MCF7 or A549 as target cells for 24 h. In the absence of target cells and after 5 days incubation, NO concentrations in NK culture supernatant in the presence or absence of IL-2 and Mu1 fraction was not exceed the assay baseline (0.5 nmol/10^6^ cells of nitrite/nitrate). But, nitrite/nitrate concentrations were elevated in supernatants of NK cells stimulated by co-culture with a target cells (MCF7 cells or A549 cells), and the maximum induction level was observed in Mu1& IL-2 pre-activated cells co-incubated with MCF7 cells (51.3 nmol/10^6^ cells of nitrite/nitrate). While, the positive control group induced with LPS or INFγ produced 39.4 and 37.2 nmol/10^6^ cells of nitrite/nitrate with MCF7 cells or A549 cells, respectively. In the control group, in the absence of IL-2 and Mu1 fraction, the induced nitrite/nitrate was 10.5 and 11.8 nmol/10^6^ cells with MCF7 cells or A549 cells, respectively.

### Cell Cycle Analysis in NK-Cancer Cells Co-culture Model

**FIGURE 11:**
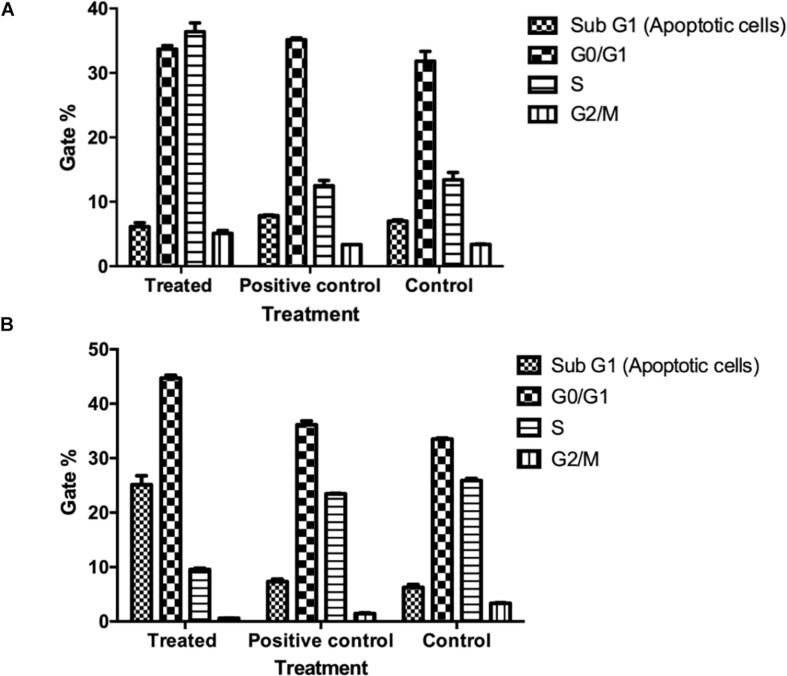
Cell cycle analysis in NK-Cancer cells co-culture model. Purified NK cells were first cultured for 5 days in the presence or absence (positive control group) of mushroom fractions Mu1 and IL-2, and following this culture, NK cells from each group were then co-culture with either A549 cells **(A)** or MCF7 **(B)** (ratio of 3:1, NK cells: cancer cells). At the end of co-culture incubation, cell cycle pattern of the all groups were cheeked using PI assays and compared with the control untreated cells and with the positive control (induced with 100 μl of *E. coli* LPS and INFγ, 10 ng/ml). mushroom fraction no. 1 (Mu1) arrest A549 cells at S phase while MCF7 treated cells arrested at G0/G1 phase.

The flowcytometry analysis of A549 (Figure 11A and Supplementary Figure S2) cells co-incubated with the pre-activated NK cells using MU1 fraction with IL-2 indicated that, the treatment arrested the A549 cells in S phase with gating 35.02% compared to 12.2 and 11.6% in negative and positive control groups, respectively. While in the positive control, NK cells were pre-activated with LPS and INFγ, the treatment arrested cells in G0/G1 phase. In addition, the pre-activated NK cells with Mu1 arrested MCF7 cells in the G0/G1 phase with high percentages of the ratios of apoptotic cells in sub G0 phase, while, NK activated cells with LPS and INFγ (positive control) arrested MCF7 cells in S phase (Figure 11B and Supplementary Figure S3).

### Analyses of the Most Active Fraction Mu1

#### Size Exclusion Chromatography Analysis

**FIGURE 12:**
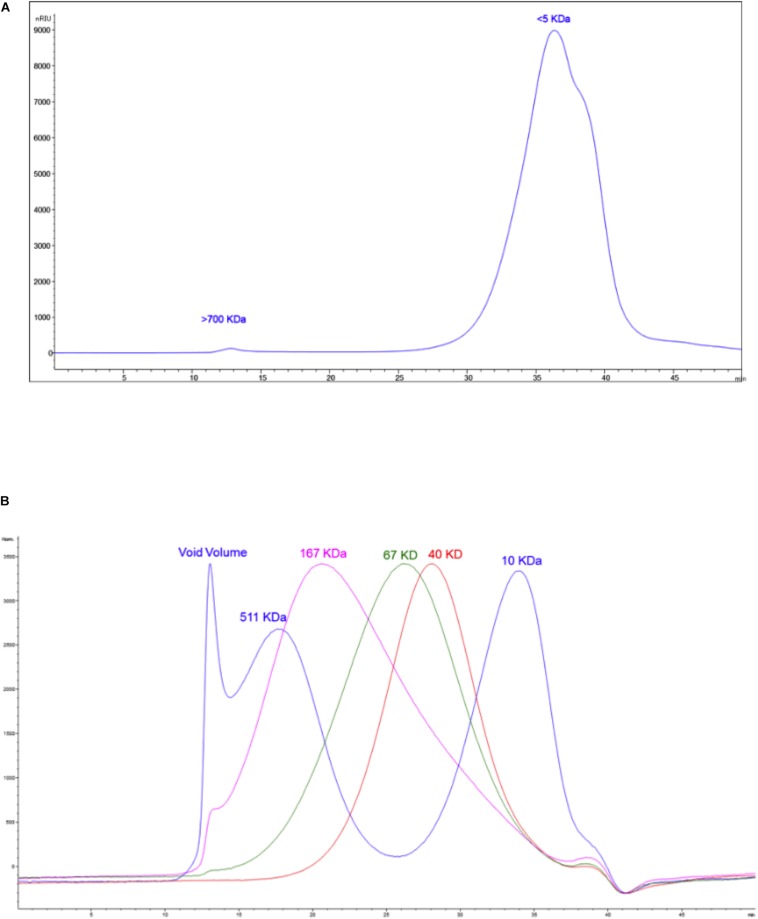
**(A)** The size exclusion chromatogram of the sample. **(B)** The overlaid chromatograms of the molecular weight standards run alongside the sample.

As a first step to further characterize the active components of Mu1, we performed size exclusion chromatography. The results (Figure 12A and Supplementary Table S7) shows the Mu1 is primarily composed of low molecular weight material around 5 kDa in size. However, a minor early eluting peak indicated the presence of a small amount of a large molecular weight component, possibly representing a high molecular weight polysaccharide. To isolate this material for further analysis, we dialyzed the sample using 8 kDa dialysis membrane for 3 days. The isolated, large molecular weight material was then used for composition and linkage analysis. Fungal cell walls often contain large amounts of mannoproteins and glucans, and smaller amounts of chitin. Thus, the large amounts of glucose and mannose along with smaller amounts of *N*-acetylglucosamine seen in the composition analysis are consistent with this (Figure 12B). The dialyzed sample is comprised mainly of glucans with amount of mannose. The presence of multiple other carbohydrate residues indicates the sample is a heterogeneous mixture of structures alongside a major glucan fraction. The overall amount of carbohydrate calculated for the sample is approximately 10%.

#### Glycosyl Composition Analysis

**FIGURE 13:**
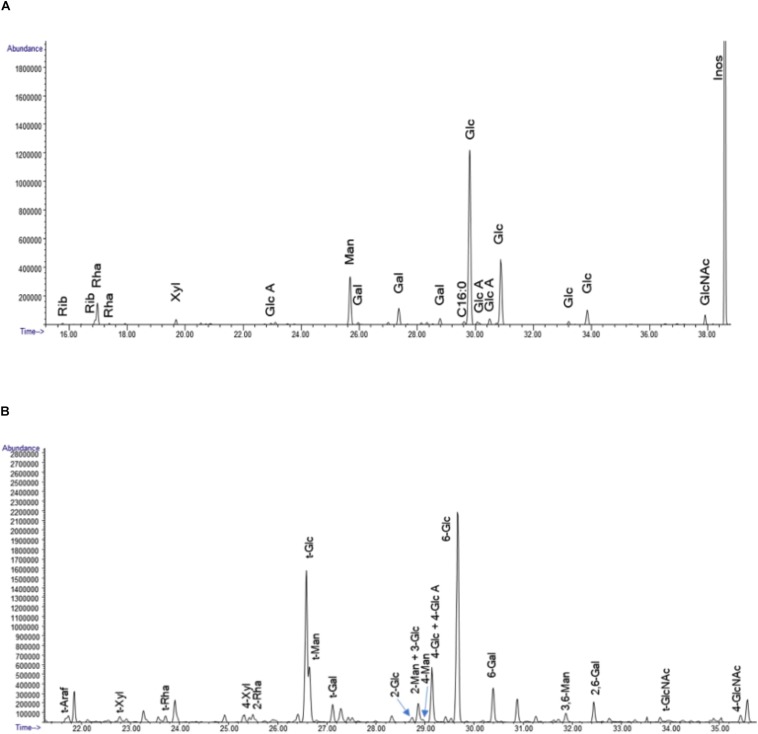
**(A)** The GC chromatograms of the TMS derivatives of the dialyzed sample. The data represent the spectrometry (GC/MS) of the per-O-trimethylsilyl (TMS) derivatives of the monosaccharide methyl glycosides produced from the sample by acidic methanolysis method. **(B)** GC chromatogram of the PMAA derivatives of the sample. The linkage method, for glycosyl linkage analysis, the samples were permethylated, reduced, repermethylated, depolymerized, reduced, and acetylated; and the resultant partially methylated alditol acetates (PMAAs) analyzed by gas chromatography-mass spectrometry (GC-MS).

The results of the linkage analysis seen in Figures 13A,B and Supplementary Table S8 are consistent with the composition analysis. The major fraction of the sample is 6-linked glucan. There is a significant amount of mannose in the sample that likely derives from mannoproteins. Some 4 linked *N*-acetyl glucosamine is also present and is likely derived from chitin in the cell wall. The remaining 20% of the linkages containing pentose, deoxyhexose, galactose and uronic acid residues indicate that the sample contains a mixture of different polysaccharides. All raw data of the composition, linkage, and SEC analysis of the polysaccharides samples after dialysis are represented in Supplementary Datasheets 1, 2.

## Discussion

Mushroom polysaccharides are known to have both direct and indirect antitumor activity on various allogeneic and syngeneic tumors and showed capability to prevent tumor metastasis. The indirect anticancer action of mushroom polysaccharides results from its regulatory roles in the activation of diverse immune responses in the host. Mushroom polysaccharides antitumor effects probably debend on NK cells direct contact as their activity is mediated through a thymus-dependent immune mechanism (Wasser, 2002).

Many reports suggest that the traditional edible fungus, *P. ostreatus*, contains different polysaccharides of both the mycelia of fungi and fruiting body (Xiao et al., 1995). But there are currently few reports on the biological effectiveness of polysaccharides isolated from *P. ostreatus* fruiting bodies, particularly with respect to immunomodulatory effects against breast, lung and liver cancer. In the current study, we separated three protein-free polysaccharides fractions. Similar results were also reported by Cao et al. (2015) for *P. ostreatus* mycelium polysaccharides. In those studies, they isolated polysaccharide moieties from *P. ostreatus* mycelium and followed by purified the isolated fractions on Sephadex G200 size-exclusion chromatography and diethylaminoethyl-52 cellulose ion-exchange chromatography. They recovered three fractions; POMP1, POMP2 and POMP3, they selected the POMP2 fraction for further purification and identification based on its higher anti-proliferation results. POMP2 also was found to not contain detectable protein or nucleic acids.

In spite of *P. ostreatus* being classified as Generally Recognized as Safe as a food product (Kodama et al., 2003), there are few reports that explained the safety profile of *P. ostreatus* extracts or polysaccharides. Deepalakshmi and Mirunalini (2014) evaluated the safety profile of a *P. ostreatus* ethanolic extract *in vivo* and concluded that the LD_50_ value of this extract was found to be >5,000 mg/kg. This confirmed that *P. ostreatus* extract has high margin of safety. Our current study determined the safety profile *in vitro* using both PBMC and NK cells. Our results indicate that PBMC are highly tolerant cells to the mushroom extract fractions, with a maximum safe dose of 20 mg/ml, while NK cells tolerated up to 5 mg/ml of the extract.

Mushroom compounds are known to combat cancers by regulating both innate and adaptive immune responses, the major components of mushroom cell wall are identified as β-glucans, this main component are thought to have a major role in initiating an immune response. One of the effective roles of β-glucans to activate immune cells could be explained by the binding of Dectin-1 and β-glucans to their own receptors on immune cells (Xu et al., 1994) that in turn activates T cells, mitogen activated protein kinases (MAPK), and nuclear factor kappa B (NF-kB) via induction different cytokines production (Dillon et al., 2006; Xu et al., 2009). Furthermore, another mushroom extracts antitumor effects could be explained by enhanced the maturation of lymphocytes and NK cells in addition to increasing the proliferation of macrophage, T helper cells, and CD4/CD8 ratio and population size (Zhang et al., 2004). While, as for NK cells (Tanaka et al., 2016), they found that Oyster mushroom enhanced IFNγ, interleukin (IL)-4, IL-5, IL-10, IL-12, IL-13, and TNF-α production in placebo-controlled human clinical trial, but the recorded increased in NK activities were relatively lower than predicted from the increased level of IFNγ without any significant differences between extract and placebo. The authors suggested that the minimal increase of NK cell activity in their study may be due to the possibility that the concentration of Oyster mushroom extract used was not sufficient for a maximum effect. While, our study recorded a modest elevation in in NK activity against MCF7 and A459 cells in the absence of IL-2; this NK-cell activity was greatly enhanced in the presence of IL-2. So, our current results explained the importance role of IL-2 to enhance the NK-cell mediated cytotoxicity induced by mushroom extract. In general, NK cells are activated by different stimulated factors as, DCs contact, MHC-1 negative cells and various cytokines as IL-1, IL-2, IL-12, IL-15, IL-18, IL-21. After this stimulations, the NK cells turned to lymphokine-activated killer cells that produce cytokines and effectors proteins as perforin, NKp44, granzymes, Fas ligand (FasL) and TRAIL (Takeda et al., 2001). Also, the activation of Tumor Necrosis Factor (TNF) family ligands that expressed on the surface of NK cells with IL-2, IL-15, IL-12, IL-18, and CD40 cytokines production induce NK cell cytotoxicity against tumor target cells and IFNγ production (Lauwerys et al., 2000). The direct effects of IFNγ on the NK cytotoxic actions against different cancer cells especially when used in clinical trials are explained. For example, Denman et al. (2012) developed an *ex vivo* NK cell expansion with large quantities for patient infusion in several clinical trials for myeloid malignancies and posterior fossa tumors, these expanded NK cells secrete large amounts of IFNγ compared to primary NK cells.

So, based on these findings we could indicated that mushroom glucans could activate NK-TNF family ligands in the presence of target cancer cells that in turn induce IFNγ production that that reflect the positive effects of IFNγ on NK -tumor cell lysis process (Aquino-Lopez et al., 2017), and that this ability is enhanced in the presence of IL-2.

Interestingly, the NKG2D receptor is mainly expressed on the cell surface of both circulating and tissue-resident NK cells with great ability to activate NK cell and induce IFN-γ cytokine production by interactions with ligands expressed on the surface of cancer cells (Wesselkamper et al., 2008). Similarly, we found that, mushroom extract could activate the cytotoxic effects of NK cells by upregulating expression of NKG2D receptors on NK cells that in turn upregulate the production of IFNγ. Moreover, NKG2D engagement could activate both gamma-delta T cells and NK cells cytolytic responses against both transfected cells and epithelial tumor cells that expressed MICA (Bauer et al., 1999). Also, the genotoxic stress of cancer cells has abilities to increase tumor cell lysis sensitivity that mediated by NKG2DL expression induction of NK cells through DDR activation (Gasser et al., 2005; Cerboni et al., 2014). But further experiment should be done to explain the opposite role on NKG2D ligand on cancer cells.

In spite of the observed upregulation in IFNγ and NKG2D in activated NK cells by mushroom extracts, we detect un expected upregulation in KIR2Dl, especially in the presence of IL-2. The KIR receptors are encoded by 15 grouped genes responsible for both inhibitory and activating receptors on chromosome. The inhibitory ones including two or three immunoglobulin domains with long cytoplasmic tails as KIR2DL or KIR3DL while, the activating KIRs including two or three short cytoplasmic tailed immunoglobulin domains (Vivier et al., 2004). In addition to KIR2DL and KIR3DL, there is KIR2DL4 (CD158d), that characterized by its central location among the KIR family members (Goodridge et al., 2003). Although it has a long cytoplasmic tail that is typical of inhibitory KIR, this receptor engagement resulted in NK cells activation for cytokines and cymokines sections not for cytotoxicity (Rajagopalan and Long, 2012). These finding could explain our up-regulation results in KIR2DL gene expression that activate NK cells for cytokine and chemokine secretion (Rajagopalan and Long, 2012) and the cytotoxic activation effect of NK cells explained by NKG2D upregulation as mentioned above. In addition to NKG2D and NKG2D genes regulations, NK cells cytotoxic effects were also accompanied by an increase in NO formation that determined by accumulation of nitrite and citrulline (Xiao et al., 1995). Interestingly, there is a closely correlation between killer activity of NK cells against tumor target cells with the nitric oxide (NO) production. Moreover, the inhibition of NO production led to block IL-2 roles to induce NK cytotoxicity against tumor cell (Yim et al., 1995; Orucevic and Lala, 1996). Furthermore, blocking NO production in mice via genetic deletion of NOS2 (inducible NOS [iNOS]) inactivation function abolished NK cell responses (Diefenbach et al., 1998) that confirming the main positive role of NO to support IL-2-NK cells induction against tumor cells. Furthermore, the induced NO (iNOS) by NK cells when cultured with tumor cells in the presence of recombinant IL-2 that in turn induced IFNγ production (Jyothi and Khar, 2000) that in turn enhance the cytotoxic effects of NK cells. All these findings also support our current results that the cytotoxic activation of NK cells using mushroom extracts is resulted from NKG2D upregulation expressions, IFNγ induction and NO production.

## Conclusion

*Pleurotus ostreatus* mushroom polysaccharides have the ability to induce NK-cell mediated cytotoxicity against lung and breast cancer cells with higher activity toward breast cancer. NK cells cytotoxic effects against cancer cells is mediated by NKG2D and IFNγ upregulation and induction and are enhanced in the presence of IL2.

## Data Availability

All datasets generated for this study are included in the manuscript and/or the Supplementary Files.

## Ethics Statement

This current study was implemented in City of Scientific Research and Technological Applications (SRTA-City), Center of Excellency for Drug Preclinical Studies (CE-DPS) Alexandria, Egypt and the protocols of blood sample collection were approved by the Research Ethical Committee at CE-DPS, SRTA-City, Alexandria, Egypt under international, national, and/or institutional guidelines. The blood samples were collected from healthy volunteers and all volunteers provided written informed consent in conformity with our all Declaration.

## Author Contributions

NE-D contributed to the practical part of anticancer, gene expression and immunology sections, contributed to preparing the idea of the manuscript, and wrote the manuscript. HE-A, AE-w, HE, Y-WH, and KD contributed to preparing the idea and revised the manuscript. AH contributed with NE-D in mushroom cultivation part.

## Conflict of Interest Statement

The authors declare that the research was conducted in the absence of any commercial or financial relationships that could be construed as a potential conflict of interest.

## Abbreviations

LPS: lipopolysaccharides
MHC: major histocompatibility complex
Mu1: mushroom extract fraction number 1
NK: natural killer cells
PBMC: peripheral blood mononuclear cells.
