# Supplementary material for: Modulation of NKG2D, KIR2DL and Cytokine Production by Pleurotus ostreatus Glucan Enhances Natural Killer Cell Cytotoxicity Toward Cancer Cells
Source: Front Cell Dev Biol. 2019 Aug 13;7:165. doi: 10.3389/fcell.2019.00165 (PMC6700253; doi:10.3389/fcell.2019.00165)

File :C:\Users\Ian\Desktop\BSB\TMS6873.D
Operator : [BSB1]Zhirui
Acquired : 17 Apr 2018 22:47 using AcqMethod TMS-EC1-1UL.M
Instrument : GC-TMS
Sample Name: KD
Misc Info :
Vial Number: 6

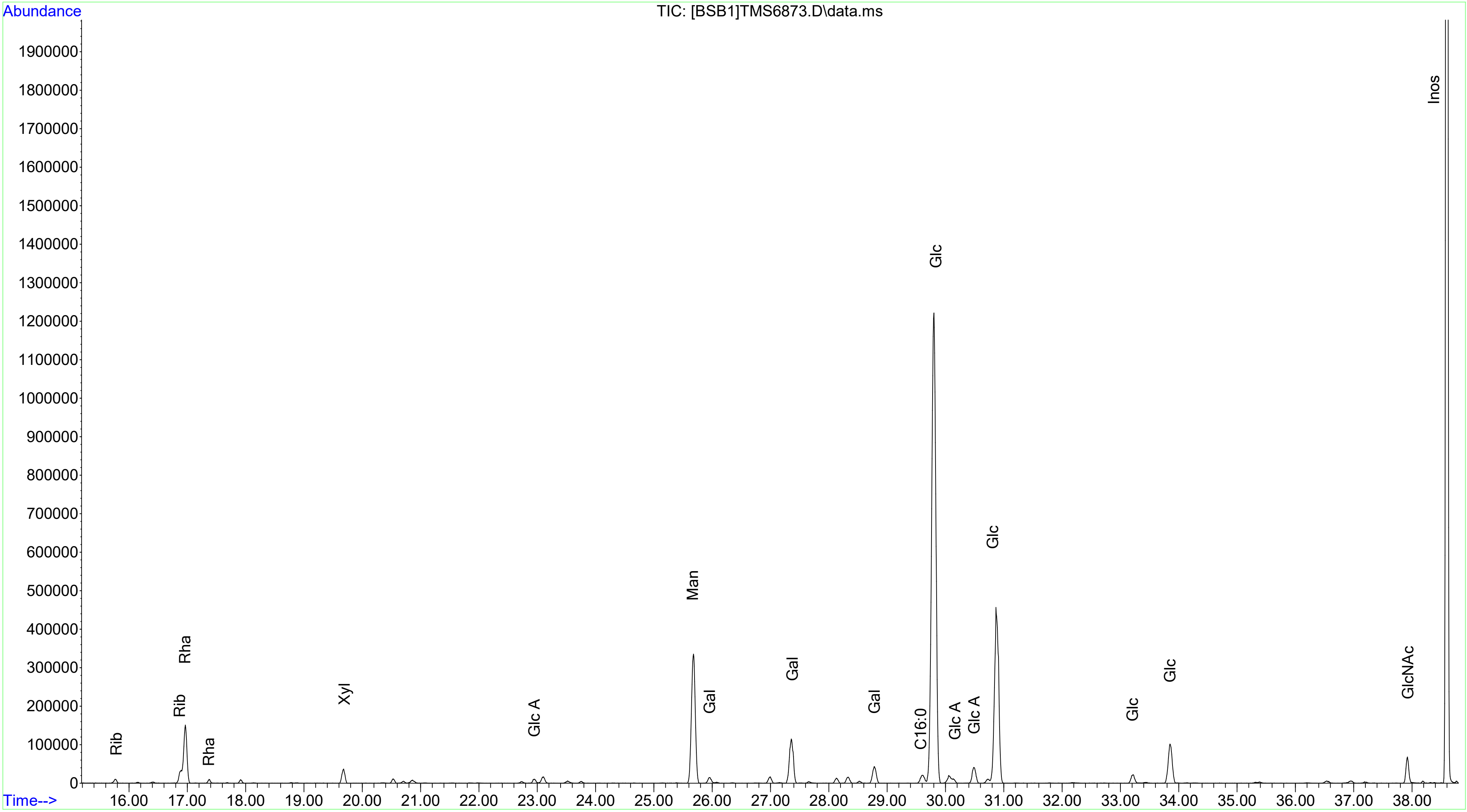

File :C:\Users\Ian\Desktop\BSB\TMS6873.D
Operator : [BSB1]Zhirui
Acquired : 17 Apr 2018 22:47 using AcqMethod TMS-EC1-1UL.M
Instrument : GC-TMS
Sample Name: KD
Misc Info :
Vial Number: 6

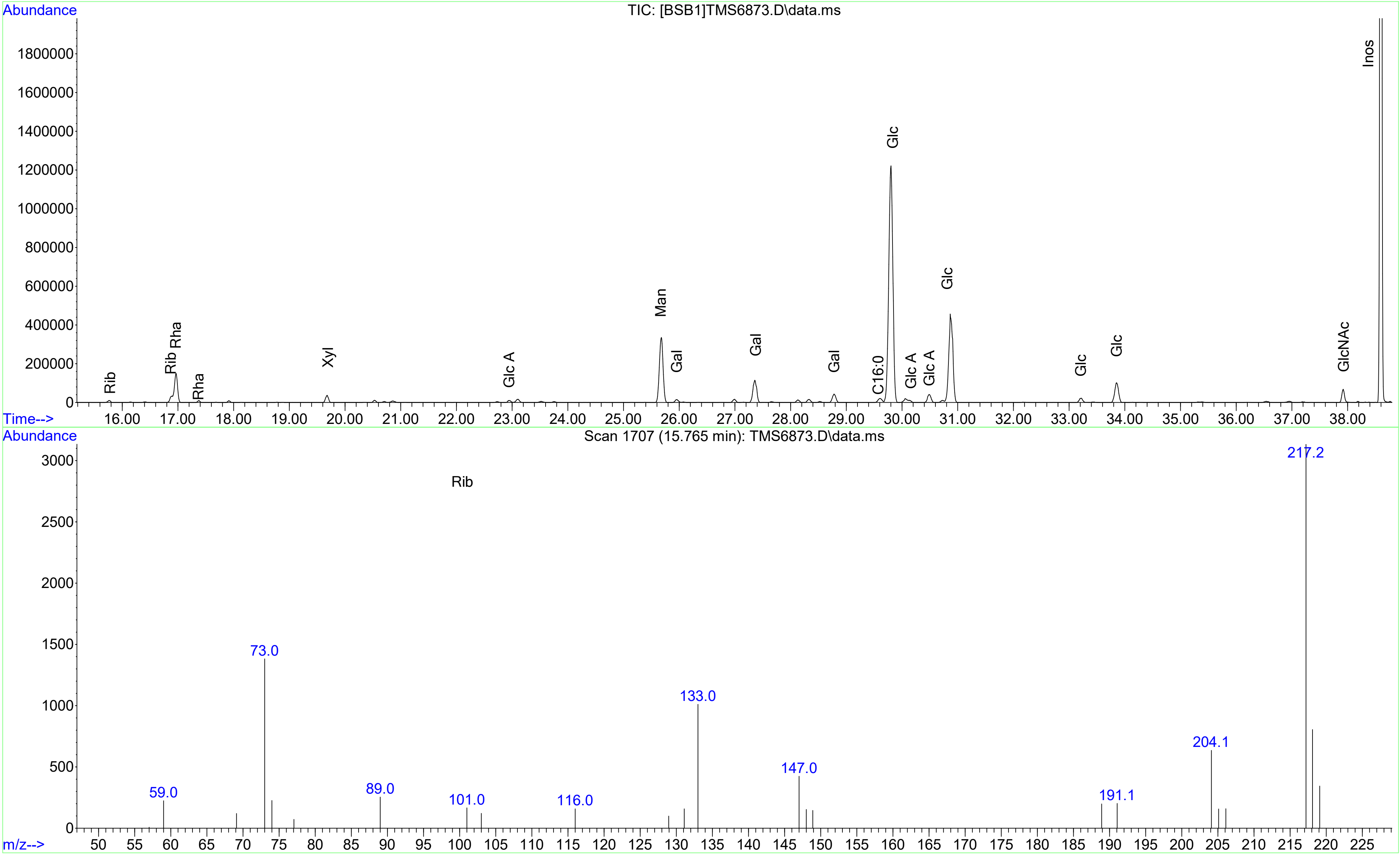

File :C:\Users\Ian\Desktop\BSB\TMS6873.D
Operator : [BSB1]Zhirui
Acquired : 17 Apr 2018 22:47 using AcqMethod TMS-EC1-1UL.M
Instrument : GC-TMS
Sample Name: KD
Misc Info :
Vial Number: 6

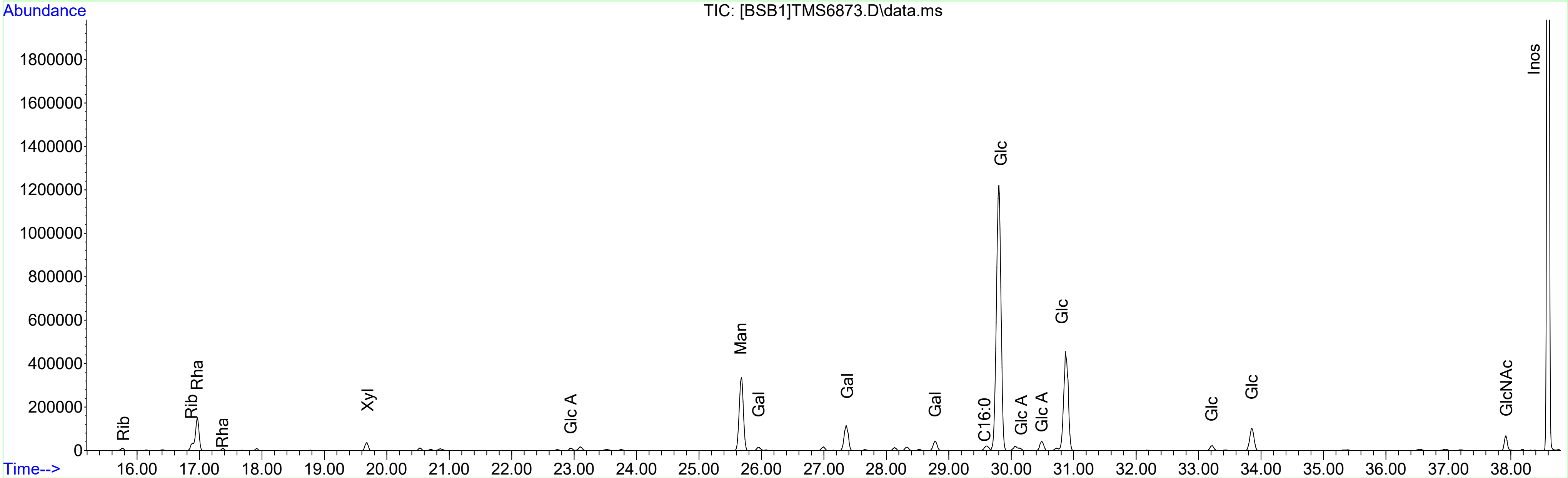


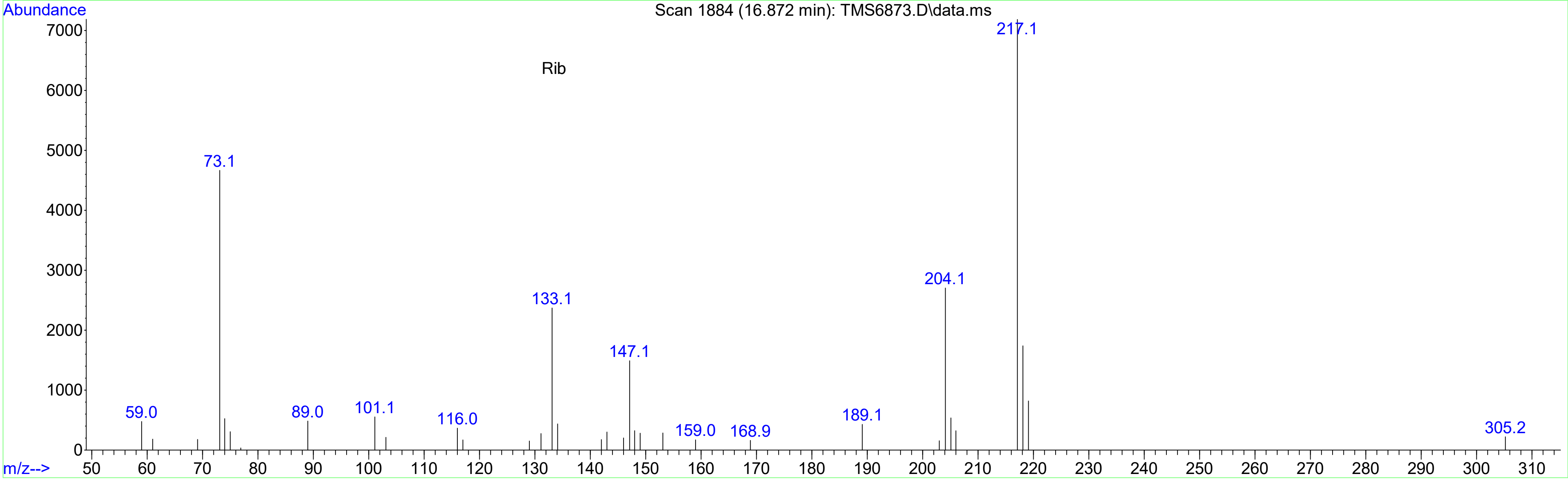

File       :C:\Users\Ian\Desktop\BSB\TMS6873.D
Operator   : [BSB1]Zhirui
Acquired   : 17 Apr 2018  22:47     using AcqMethod TMS-EC1-1UL.M
Instrument :    GC-TMS
Sample Name: KD
Misc Info  :
Vial Number: 6

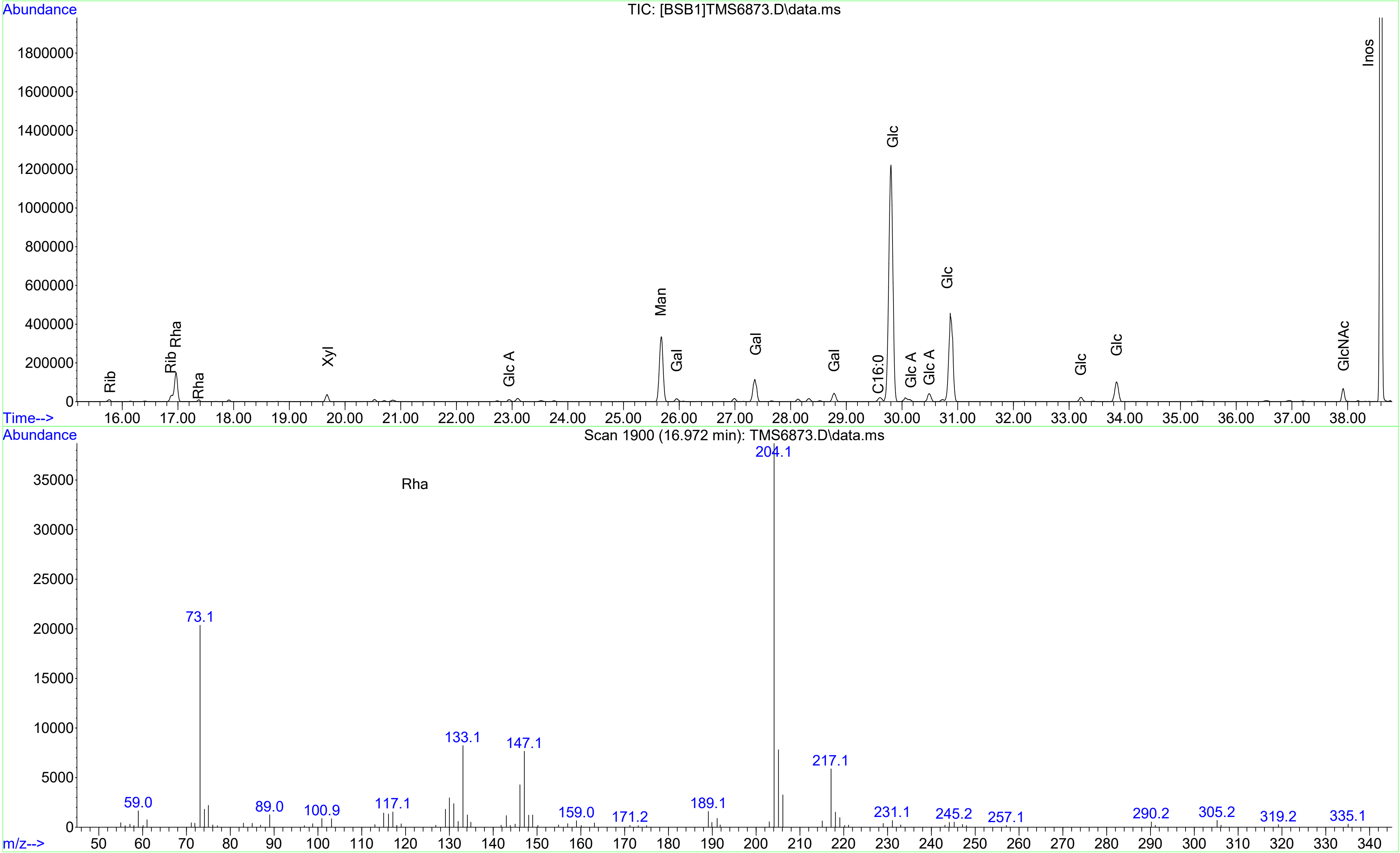

File :C:\Users\Ian\Desktop\BSB\TMS6873.D
Operator : [BSB1]Zhirui
Acquired : 17 Apr 2018 22:47 using AcqMethod TMS-EC1-1UL.M
Instrument : GC-TMS
Sample Name: KD
Misc Info :
Vial Number: 6

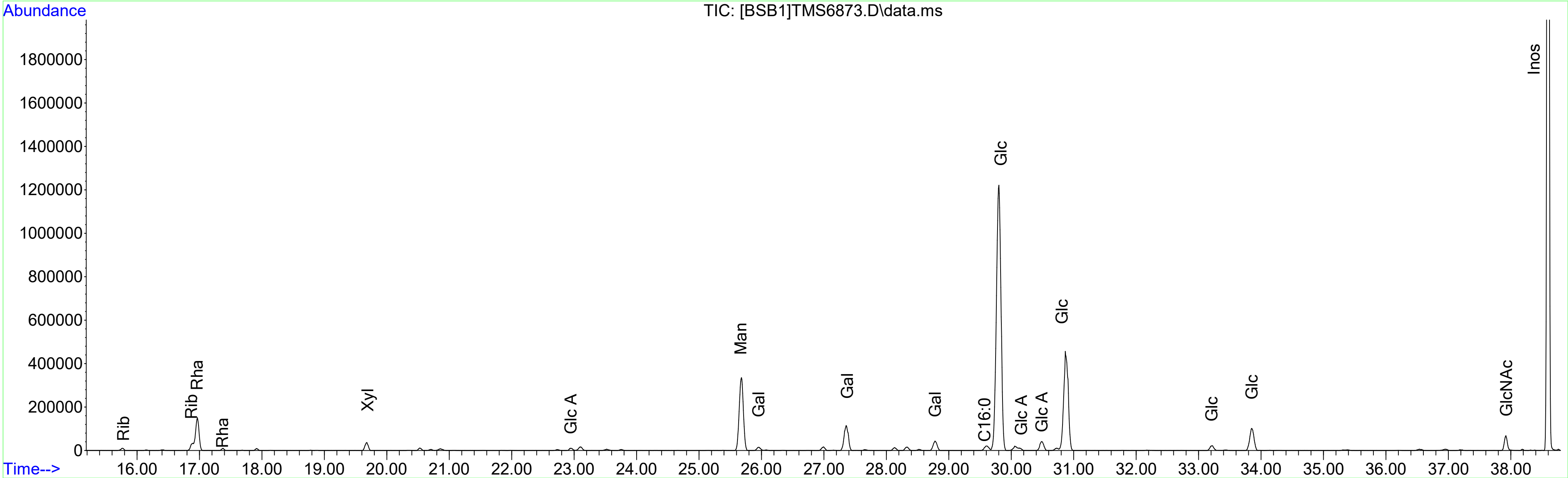


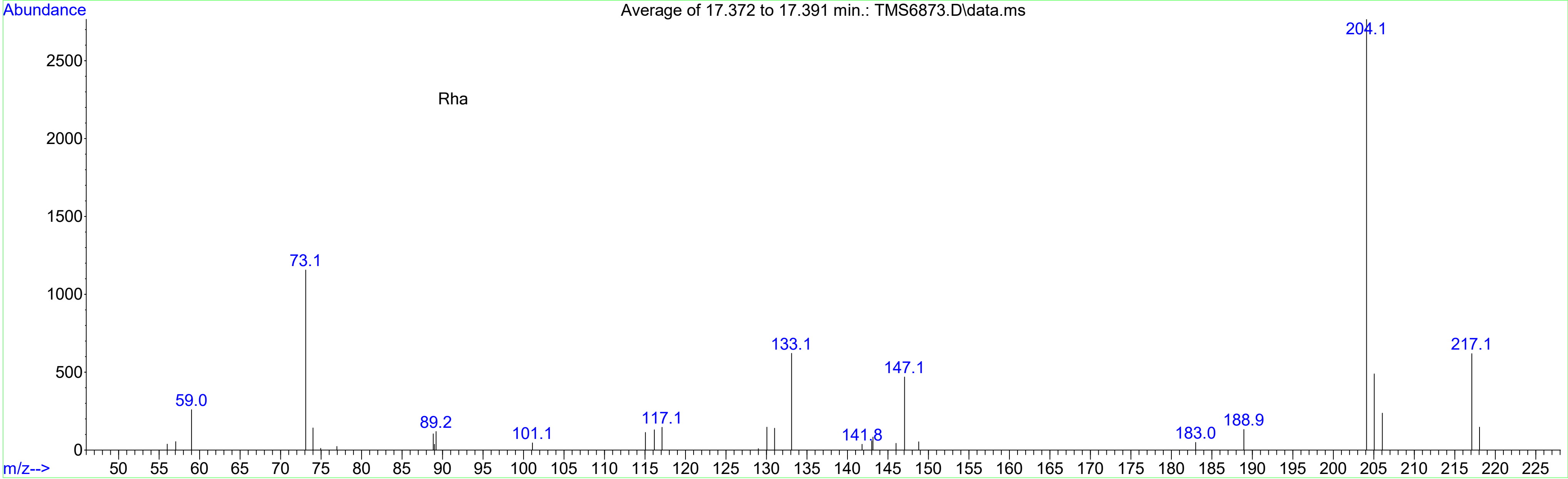

File :C:\Users\Ian\Desktop\BSB\TMS6873.D
Operator : [BSB1]Zhirui
Acquired : 17 Apr 2018 22:47 using AcqMethod TMS-EC1-1UL.M
Instrument : GC-TMS
Sample Name: KD
Misc Info :
Vial Number: 6

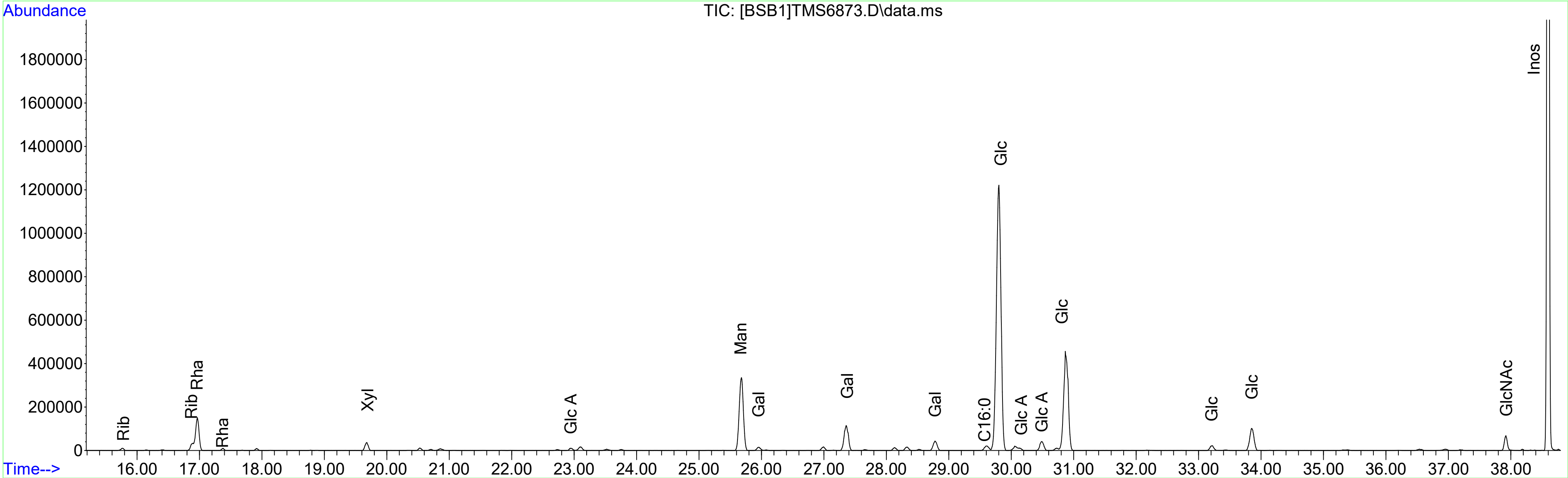


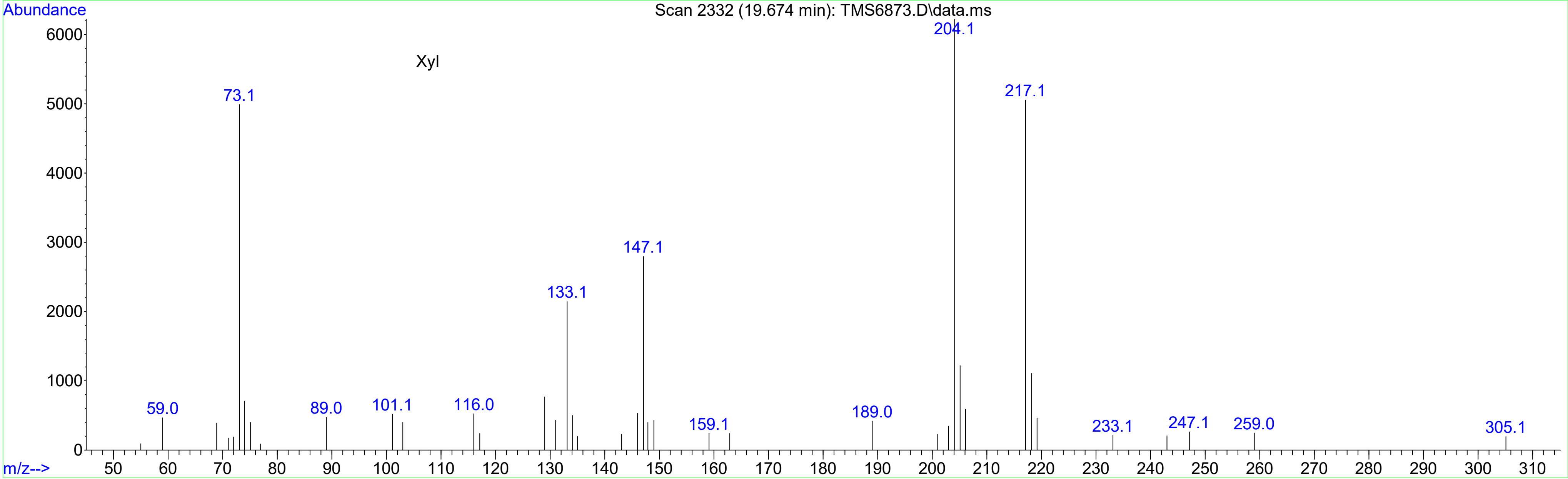

File :C:\Users\Ian\Desktop\BSB\TMS6873.D
Operator : [BSB1]Zhirui
Acquired : 17 Apr 2018 22:47 using AcqMethod TMS-EC1-1UL.M
Instrument : GC-TMS
Sample Name: KD
Misc Info :
Vial Number: 6

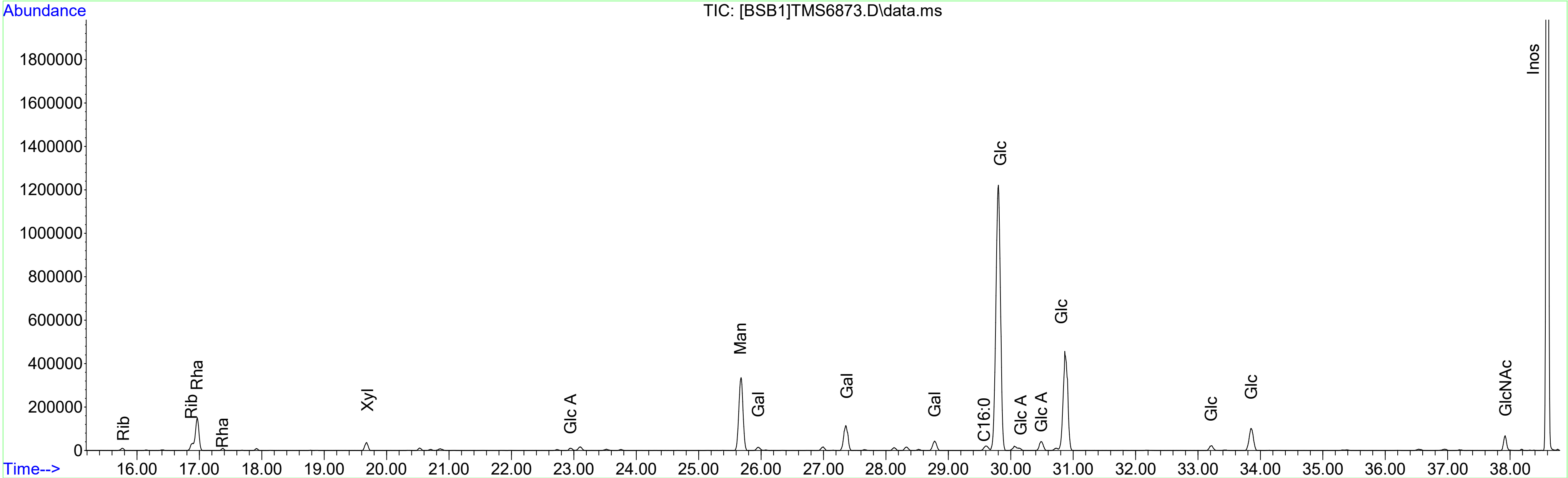


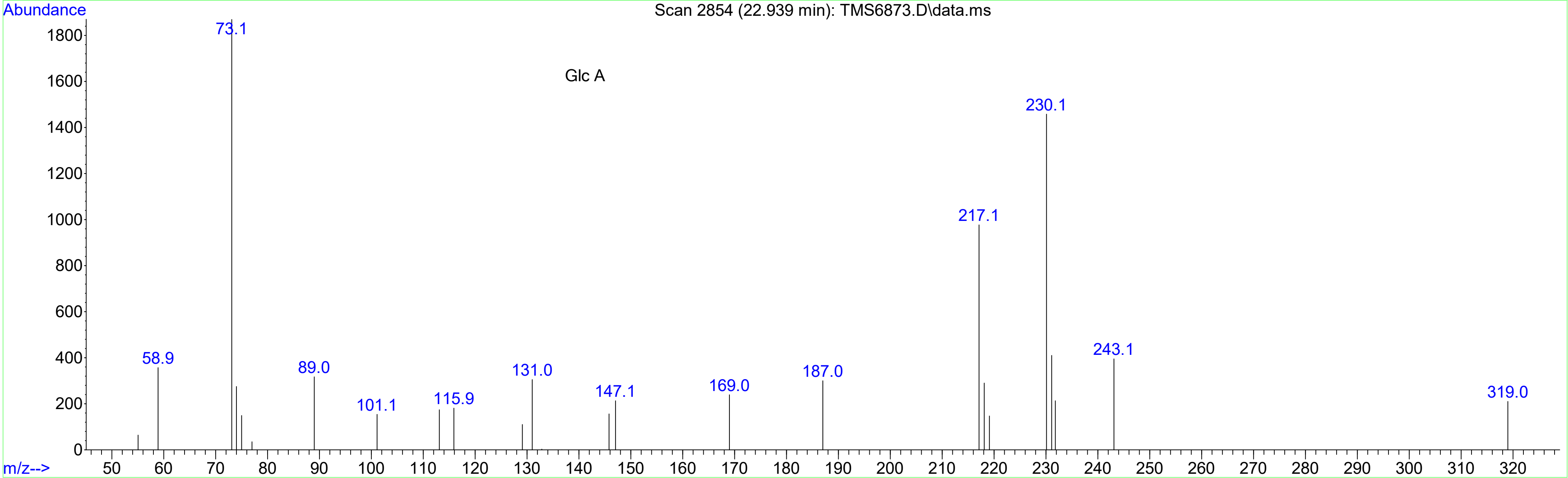

File :C:\Users\Ian\Desktop\BSB\TMS6873.D
Operator : [BSB1]Zhirui
Acquired : 17 Apr 2018 22:47 using AcqMethod TMS-EC1-1UL.M
Instrument : GC-TMS
Sample Name: KD
Misc Info :
Vial Number: 6

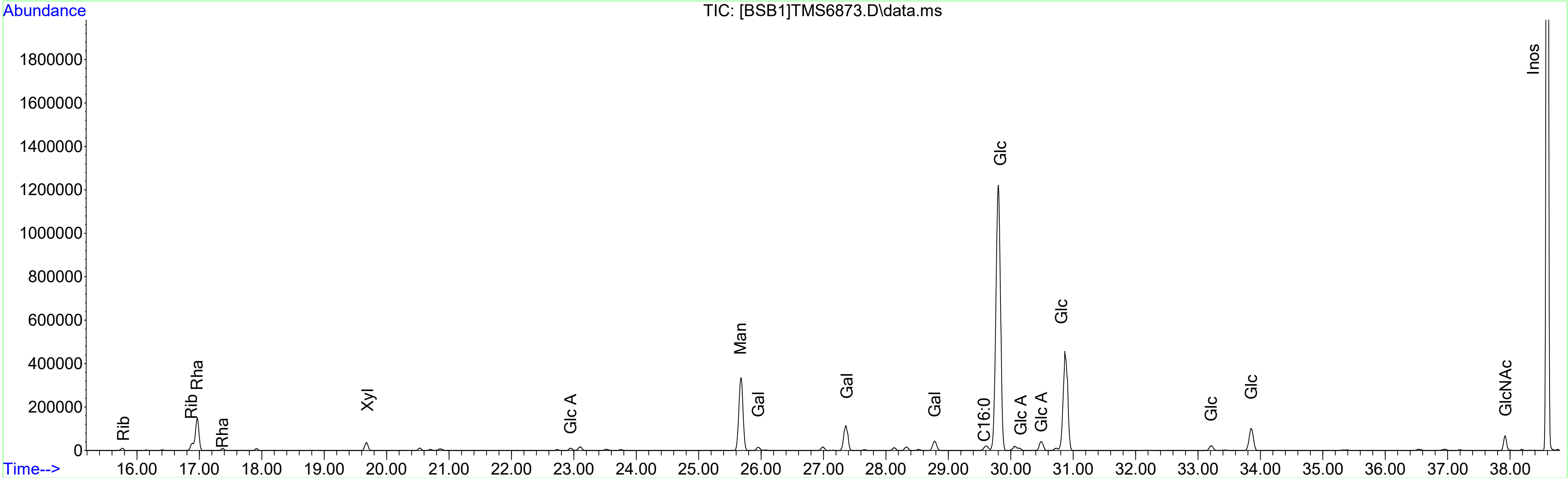


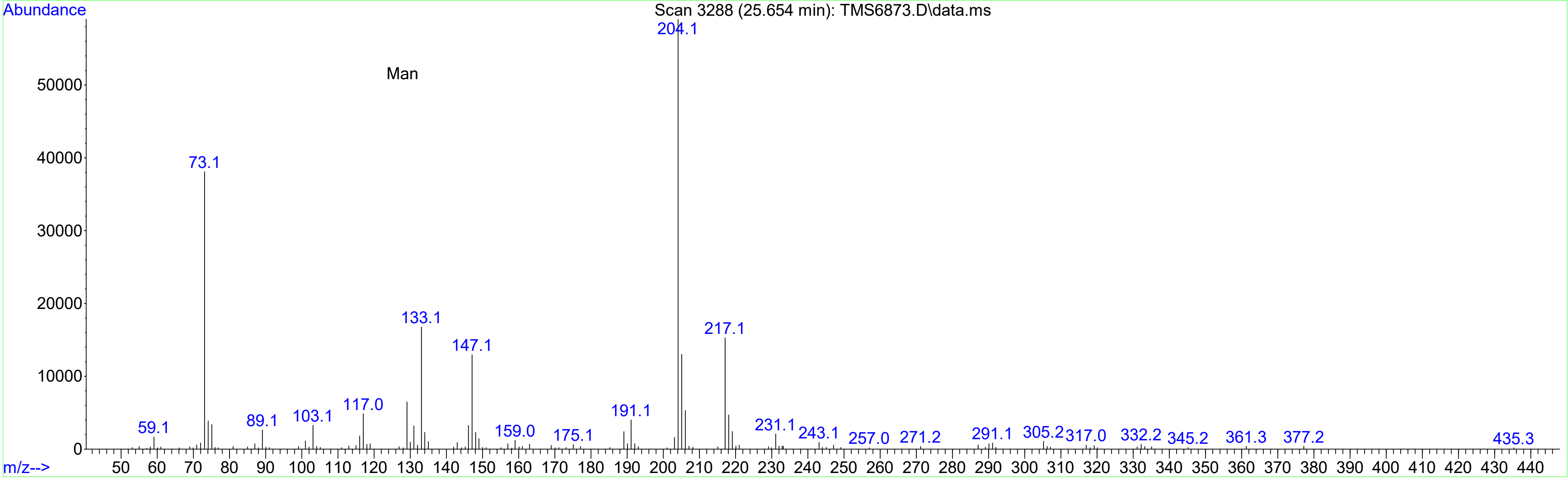

File :C:\Users\Ian\Desktop\BSB\TMS6873.D
Operator : [BSB1]Zhirui
Acquired : 17 Apr 2018 22:47 using AcqMethod TMS-EC1-1UL.M
Instrument : GC-TMS
Sample Name: KD
Misc Info :
Vial Number: 6

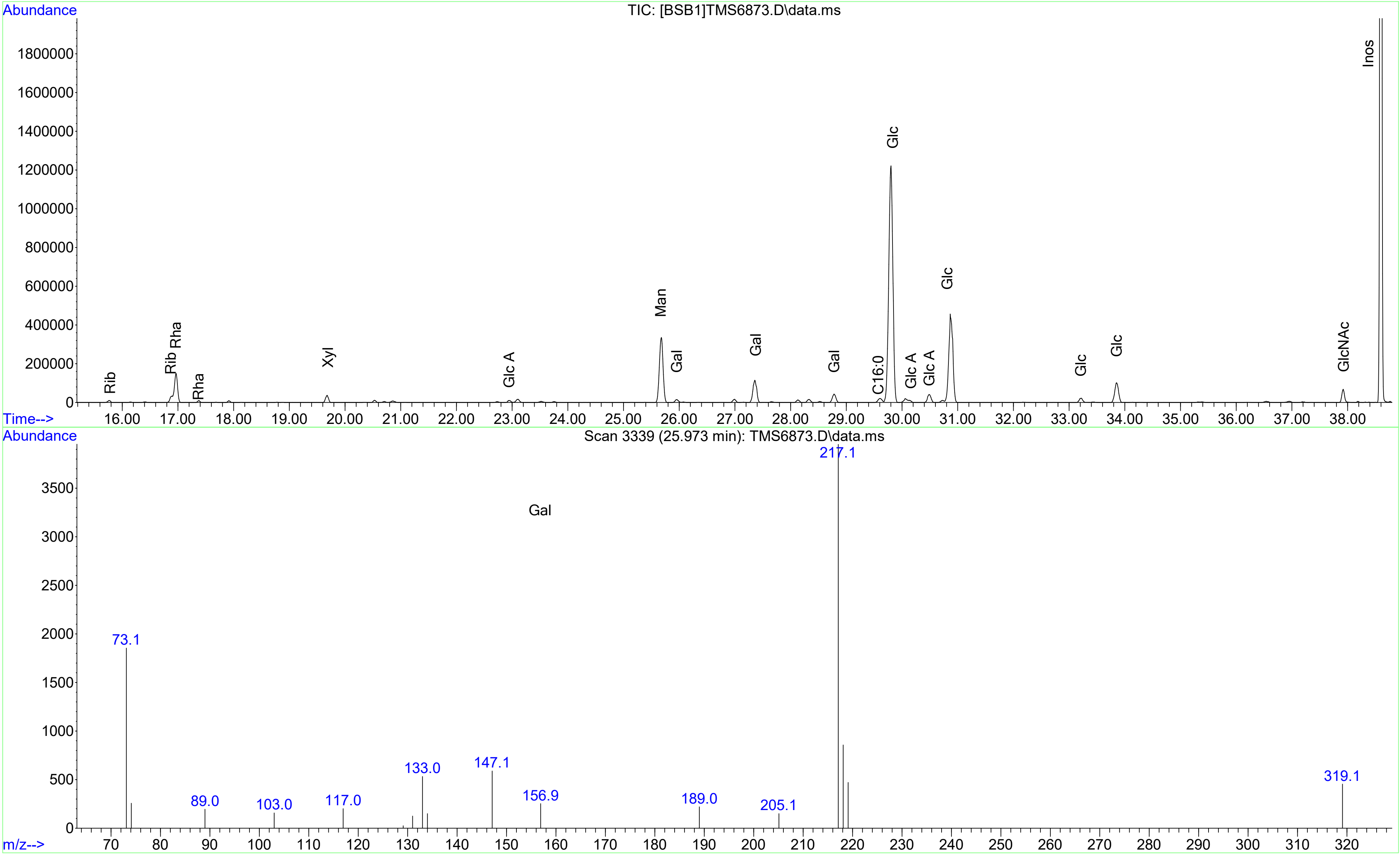

File :C:\Users\Ian\Desktop\BSB\TMS6873.D
Operator : [BSB1]Zhirui
Acquired : 17 Apr 2018 22:47 using AcqMethod TMS-EC1-1UL.M
Instrument : GC-TMS
Sample Name: KD
Misc Info :
Vial Number: 6

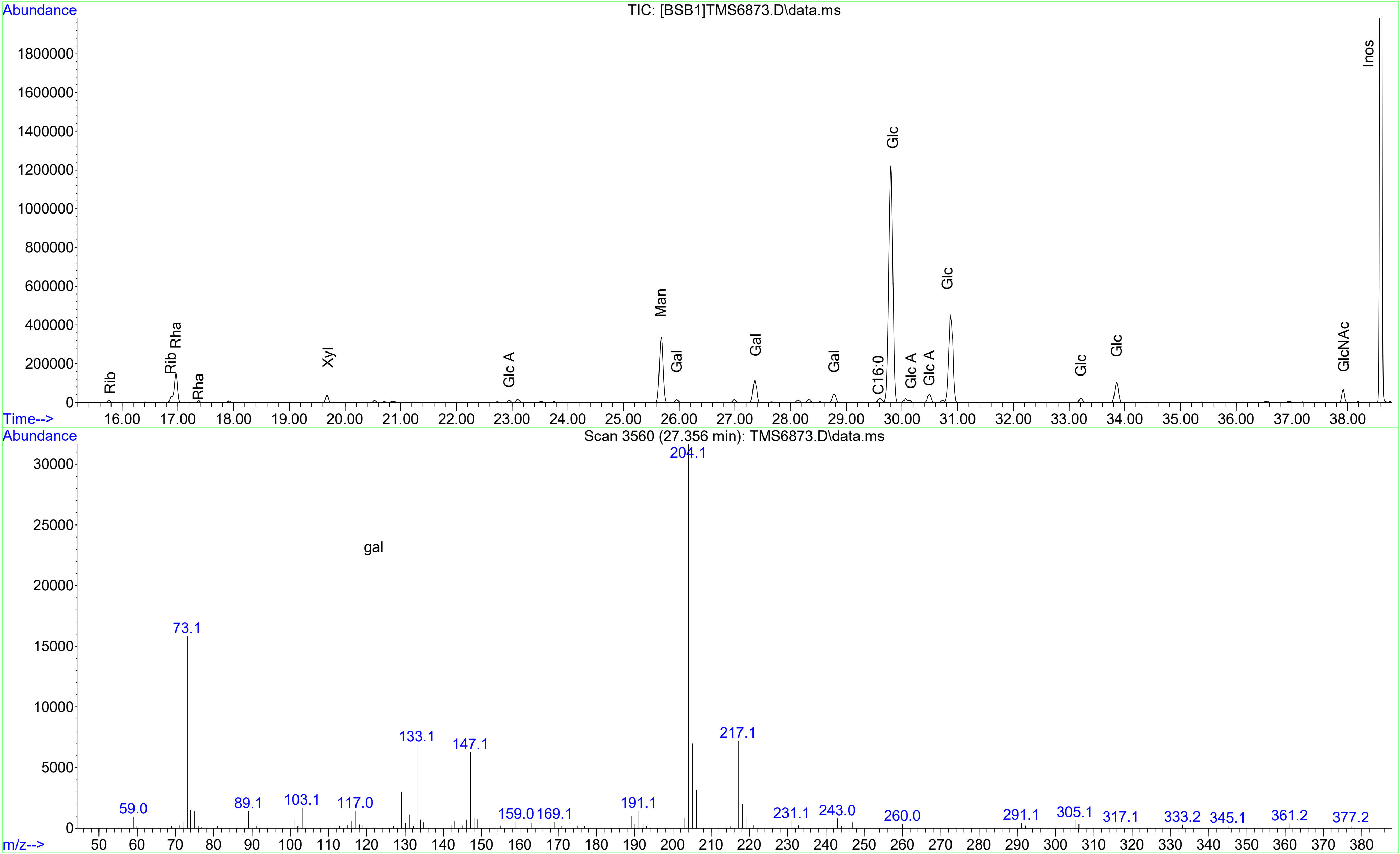

File :C:\Users\Ian\Desktop\BSB\TMS6873.D
Operator : [BSB1]Zhirui
Acquired : 17 Apr 2018 22:47 using AcqMethod TMS-EC1-1UL.M
Instrument : GC-TMS
Sample Name: KD
Misc Info :
Vial Number: 6

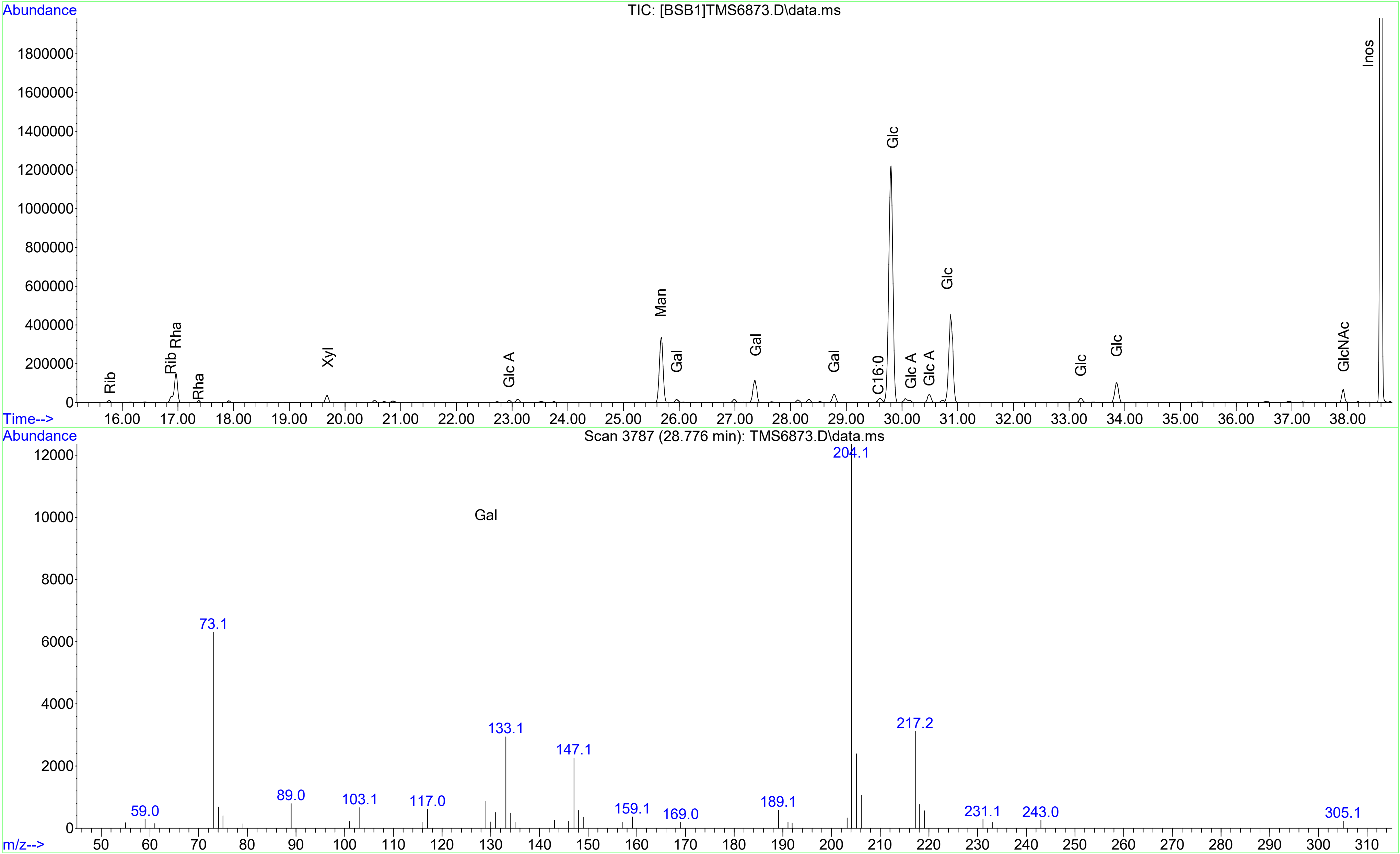

File :C:\Users\Ian\Desktop\BSB\TMS6873.D
Operator : [BSB1]Zhirui
Acquired : 17 Apr 2018 22:47 using AcqMethod TMS-EC1-1UL.M
Instrument : GC-TMS
Sample Name: KD
Misc Info :
Vial Number: 6

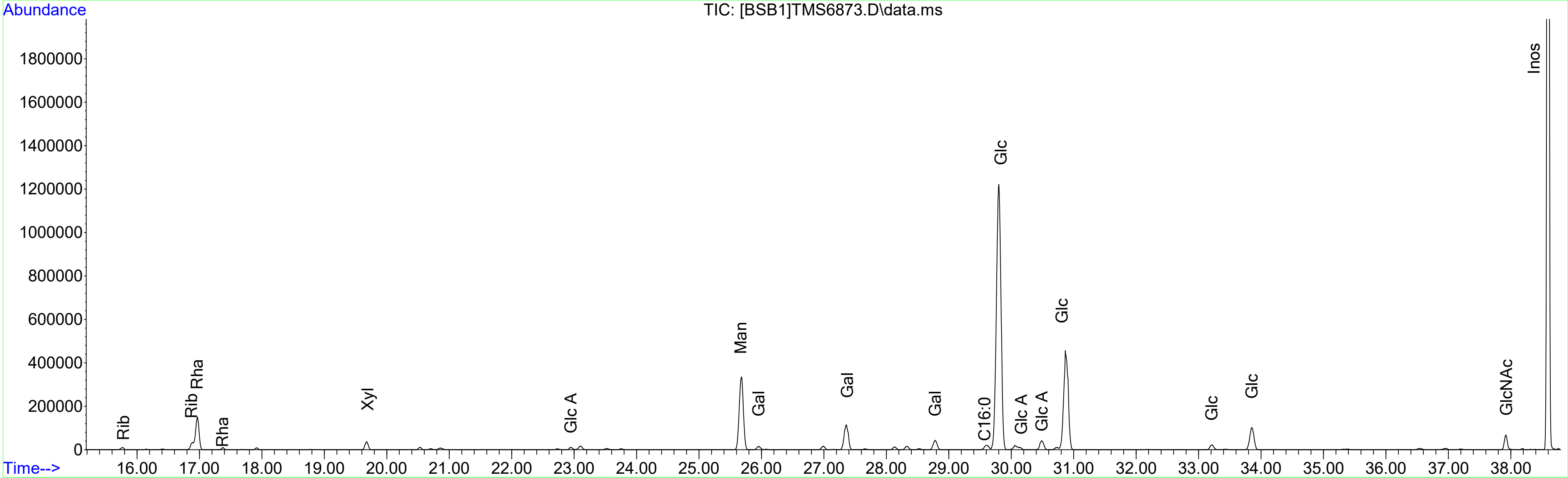


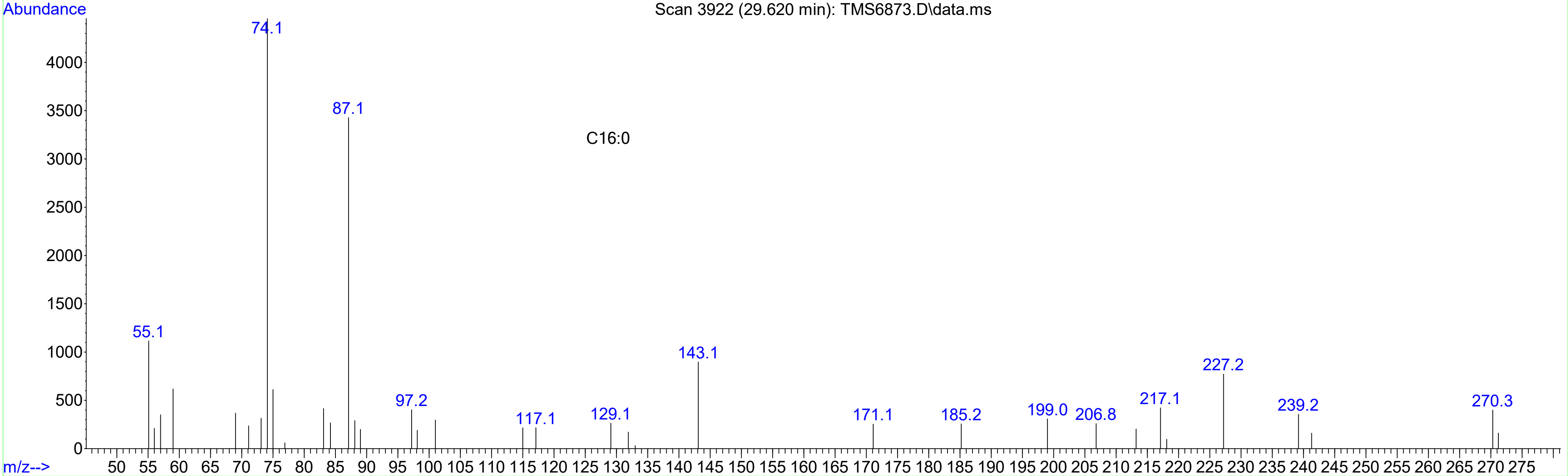

File :C:\Users\Ian\Desktop\BSB\TMS6873.D
Operator : [BSB1]Zhirui
Acquired : 17 Apr 2018 22:47 using AcqMethod TMS-EC1-1UL.M
Instrument : GC-TMS
Sample Name: KD
Misc Info :
Vial Number: 6

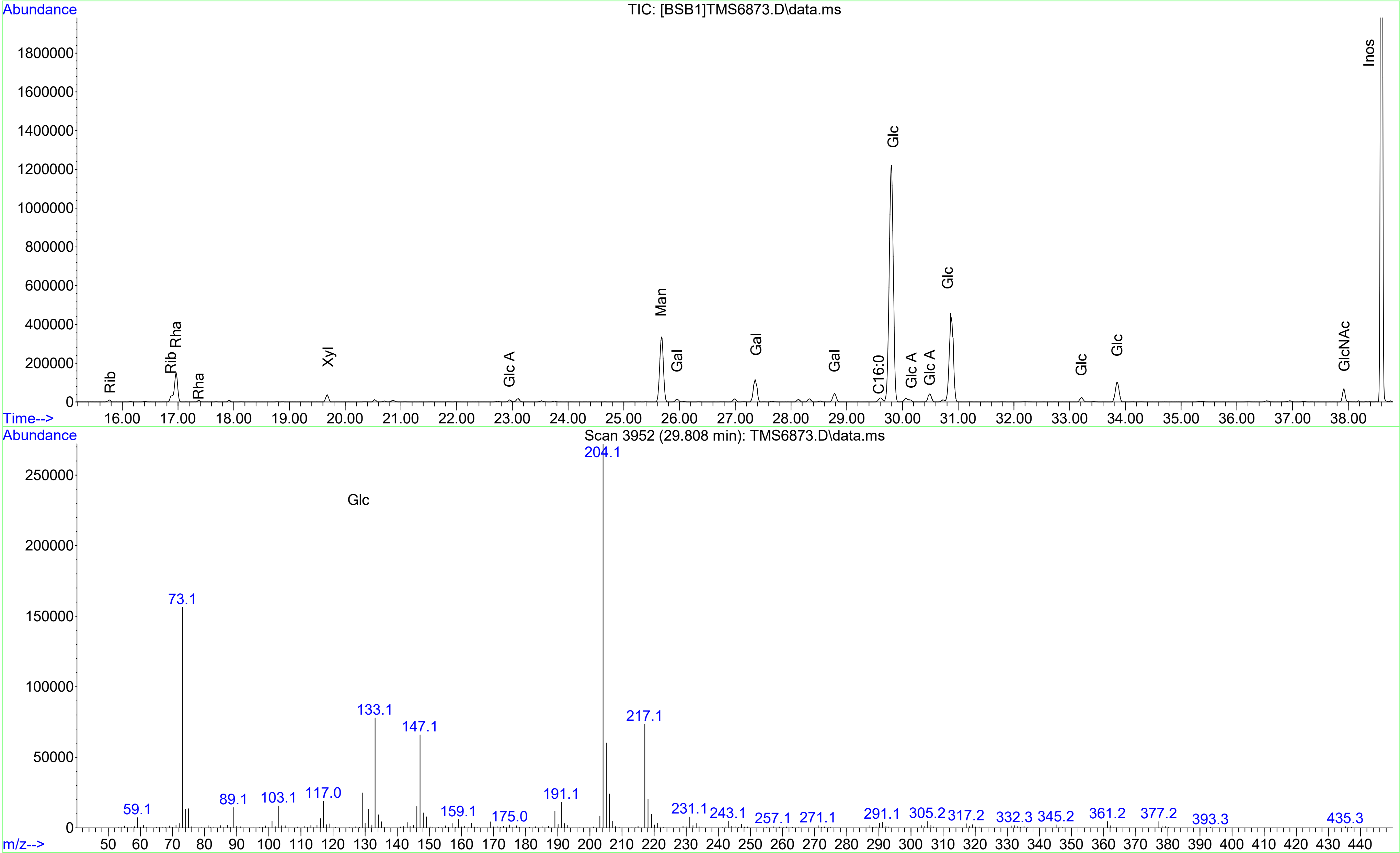

File :C:\Users\Ian\Desktop\BSB\TMS6873.D
Operator : [BSB1]Zhirui
Acquired : 17 Apr 2018 22:47 using AcqMethod TMS-EC1-1UL.M
Instrument : GC-TMS
Sample Name: KD
Misc Info :
Vial Number: 6

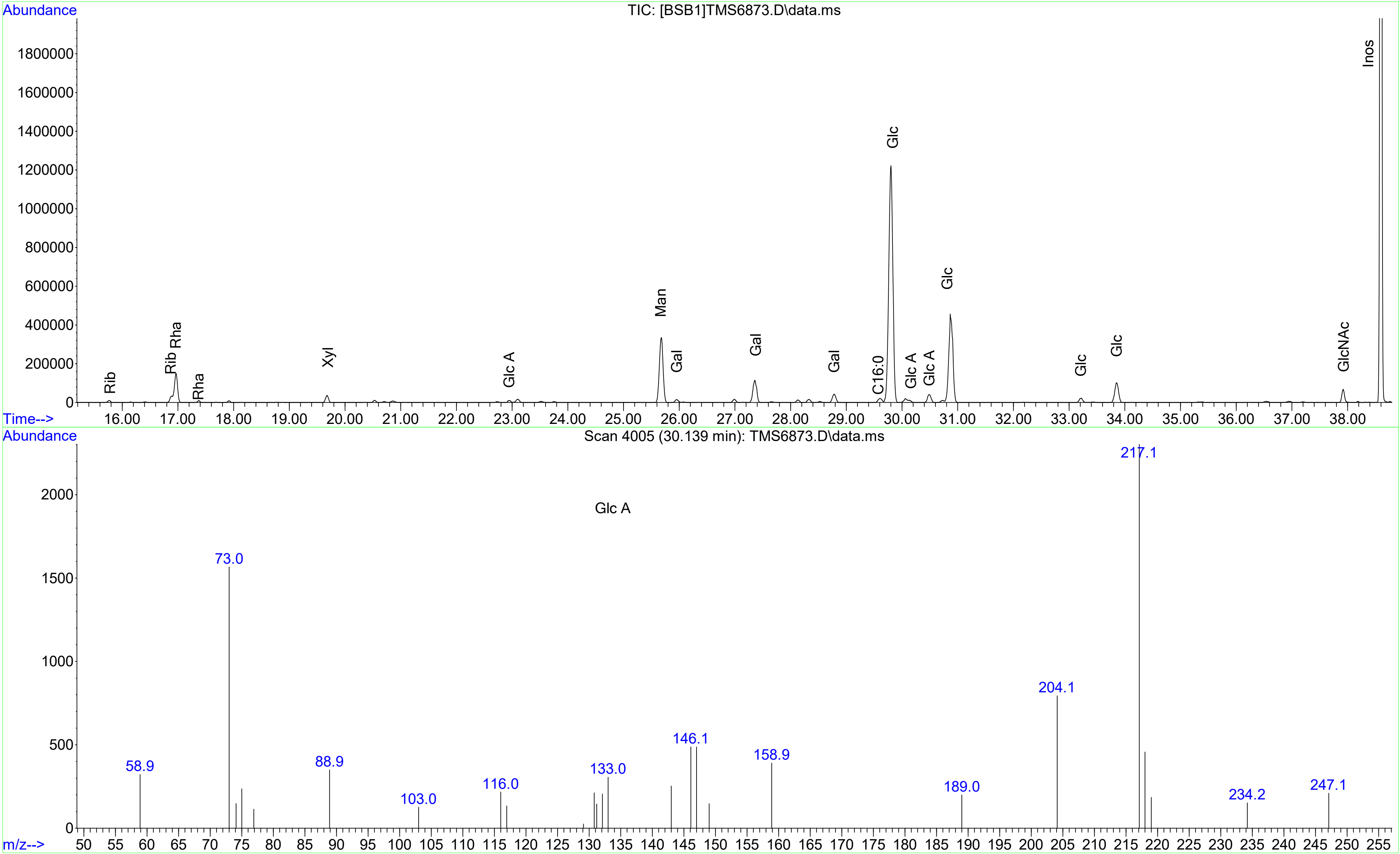

File :C:\Users\Ian\Desktop\BSB\TMS6873.D
Operator : [BSB1]Zhirui
Acquired : 17 Apr 2018 22:47 using AcqMethod TMS-EC1-1UL.M
Instrument : GC-TMS
Sample Name: KD
Misc Info :
Vial Number: 6

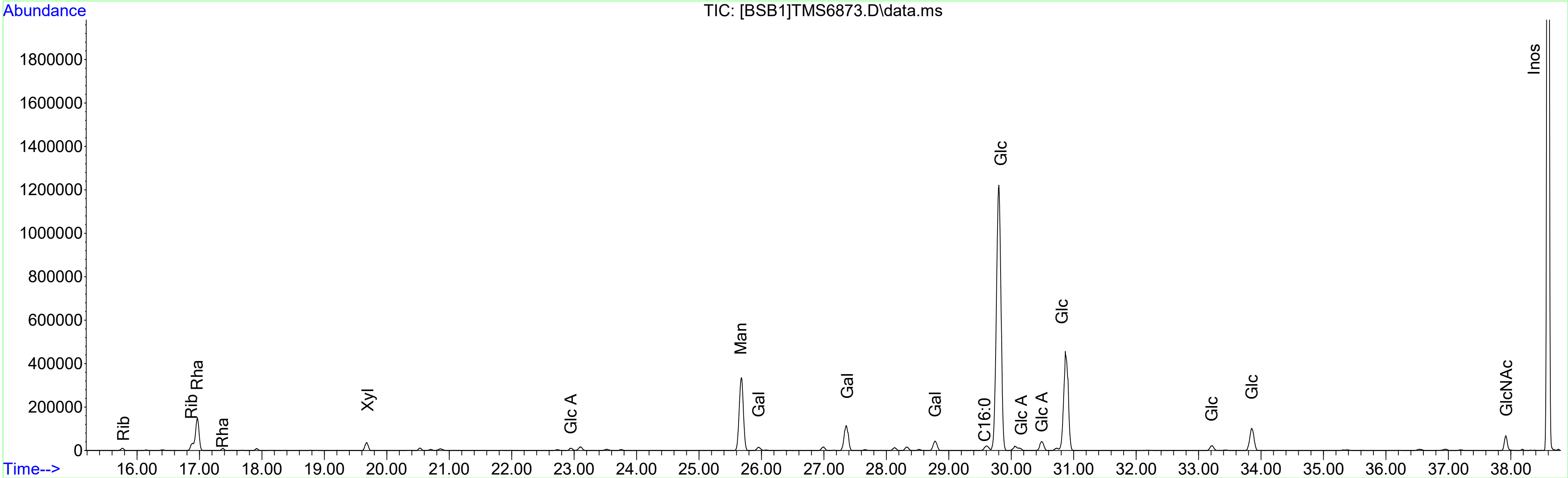


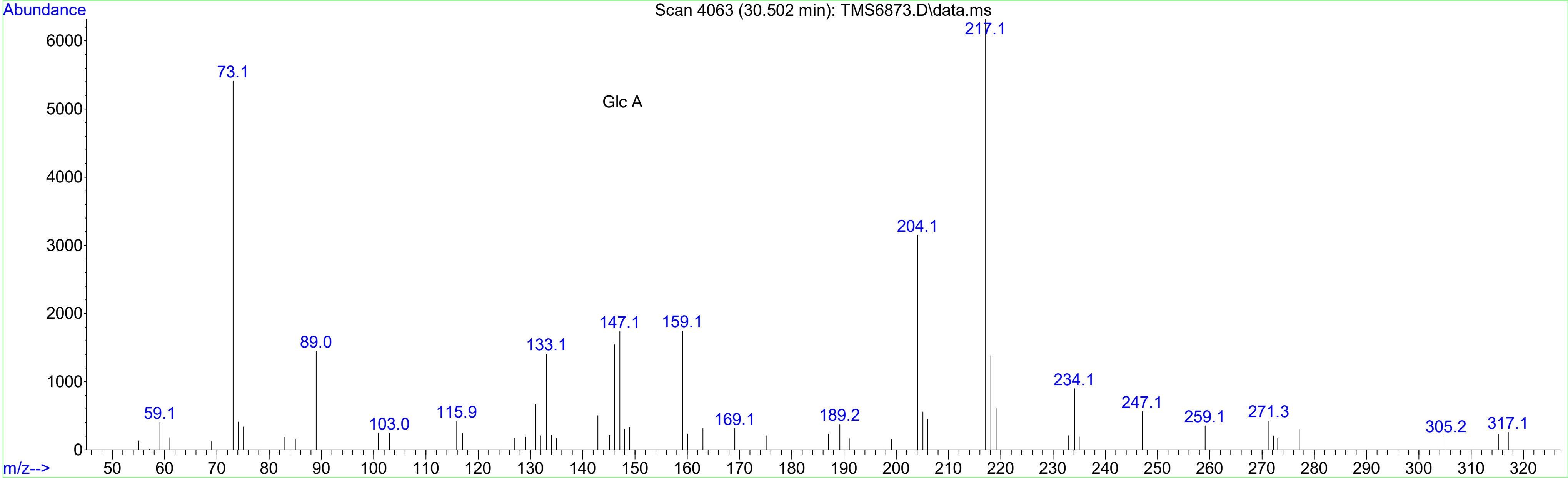

File :C:\Users\Ian\Desktop\BSB\TMS6873.D
Operator : [BSB1]Zhirui
Acquired : 17 Apr 2018 22:47 using AcqMethod TMS-EC1-1UL.M
Instrument : GC-TMS
Sample Name: KD
Misc Info :
Vial Number: 6

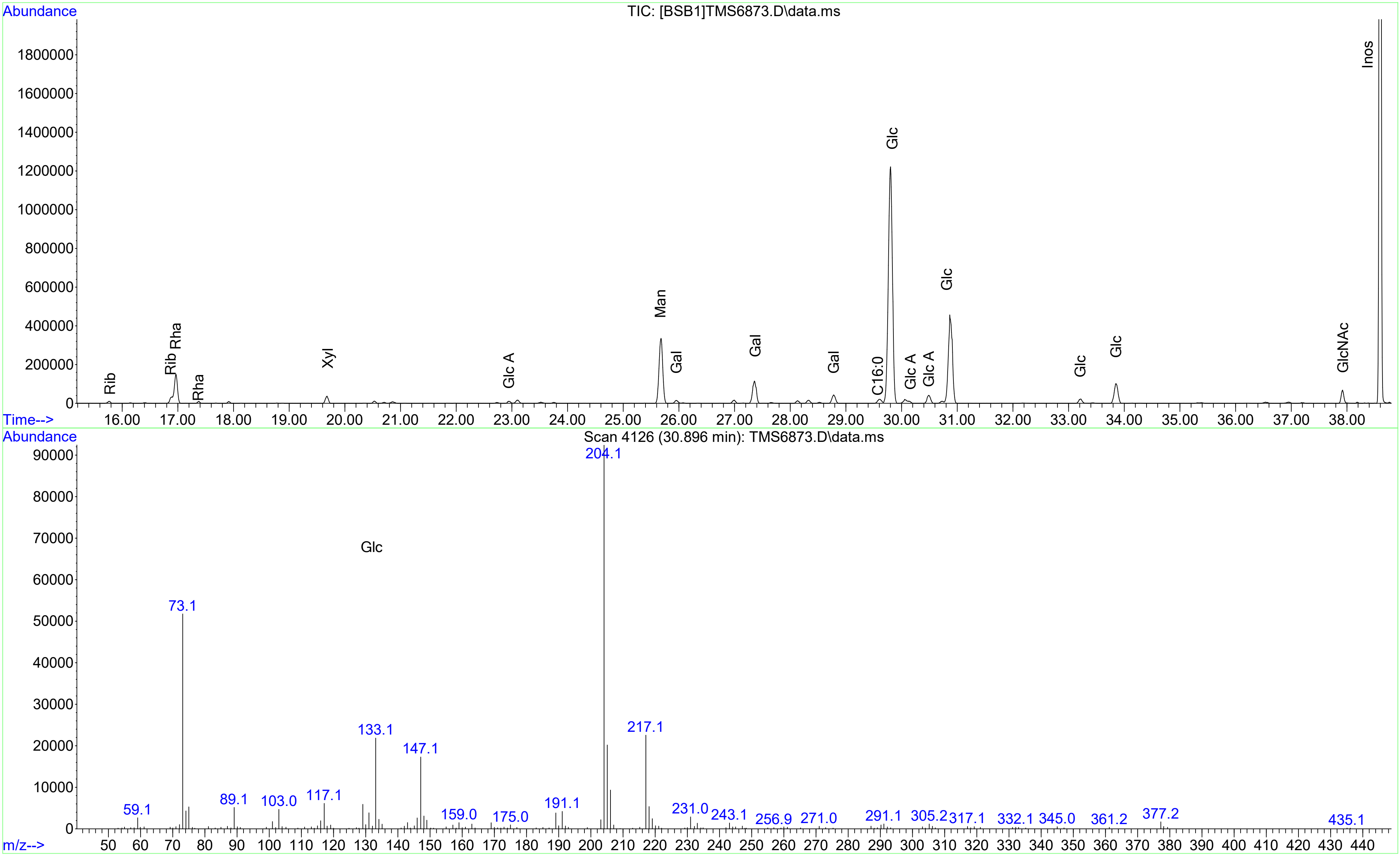

File :C:\Users\Ian\Desktop\BSB\TMS6873.D
Operator : [BSB1]Zhirui
Acquired : 17 Apr 2018 22:47 using AcqMethod TMS-EC1-1UL.M
Instrument : GC-TMS
Sample Name: KD
Misc Info :
Vial Number: 6

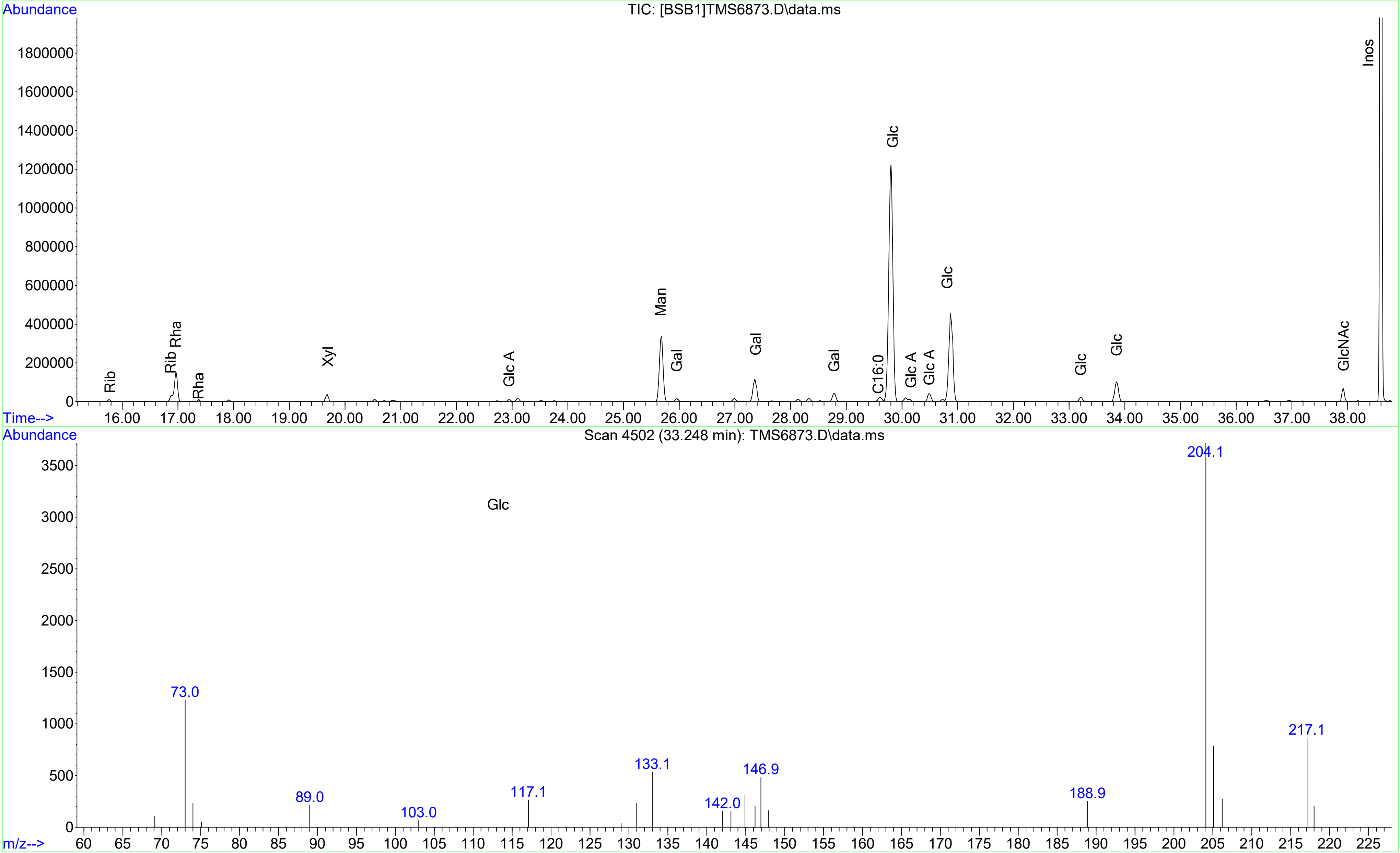

File       :C:\Users\Ian\Desktop\BSB\TMS6873.D
Operator   : [BSB1]Zhirui
Acquired   : 17 Apr 2018  22:47     using AcqMethod TMS-EC1-1UL.M
Instrument :    GC-TMS
Sample Name: KD
Misc Info  :
Vial Number: 6

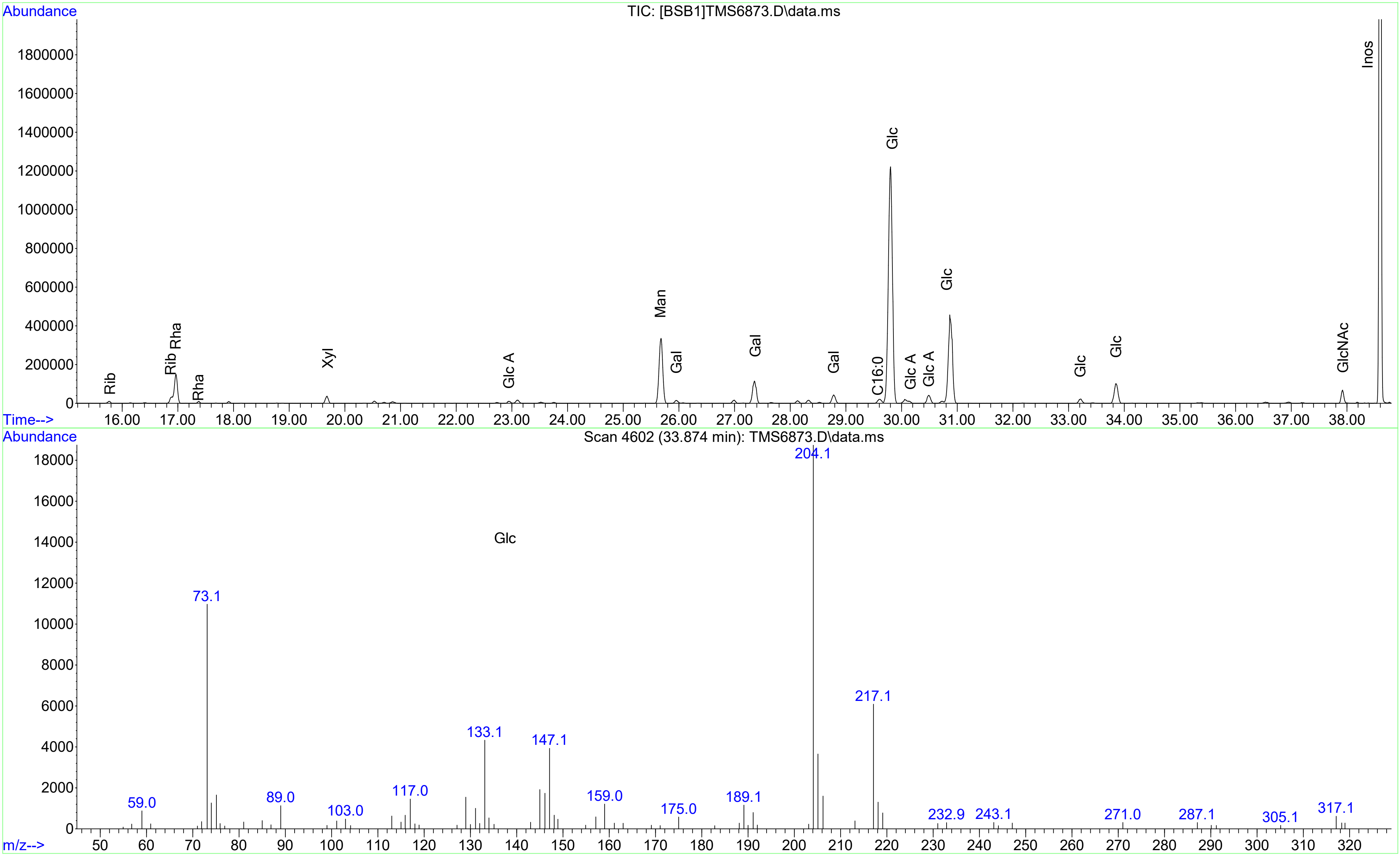

File :C:\Users\Ian\Desktop\BSB\TMS6873.D
Operator : [BSB1]Zhirui
Acquired : 17 Apr 2018 22:47 using AcqMethod TMS-EC1-1UL.M
Instrument : GC-TMS
Sample Name: KD
Misc Info :
Vial Number: 6

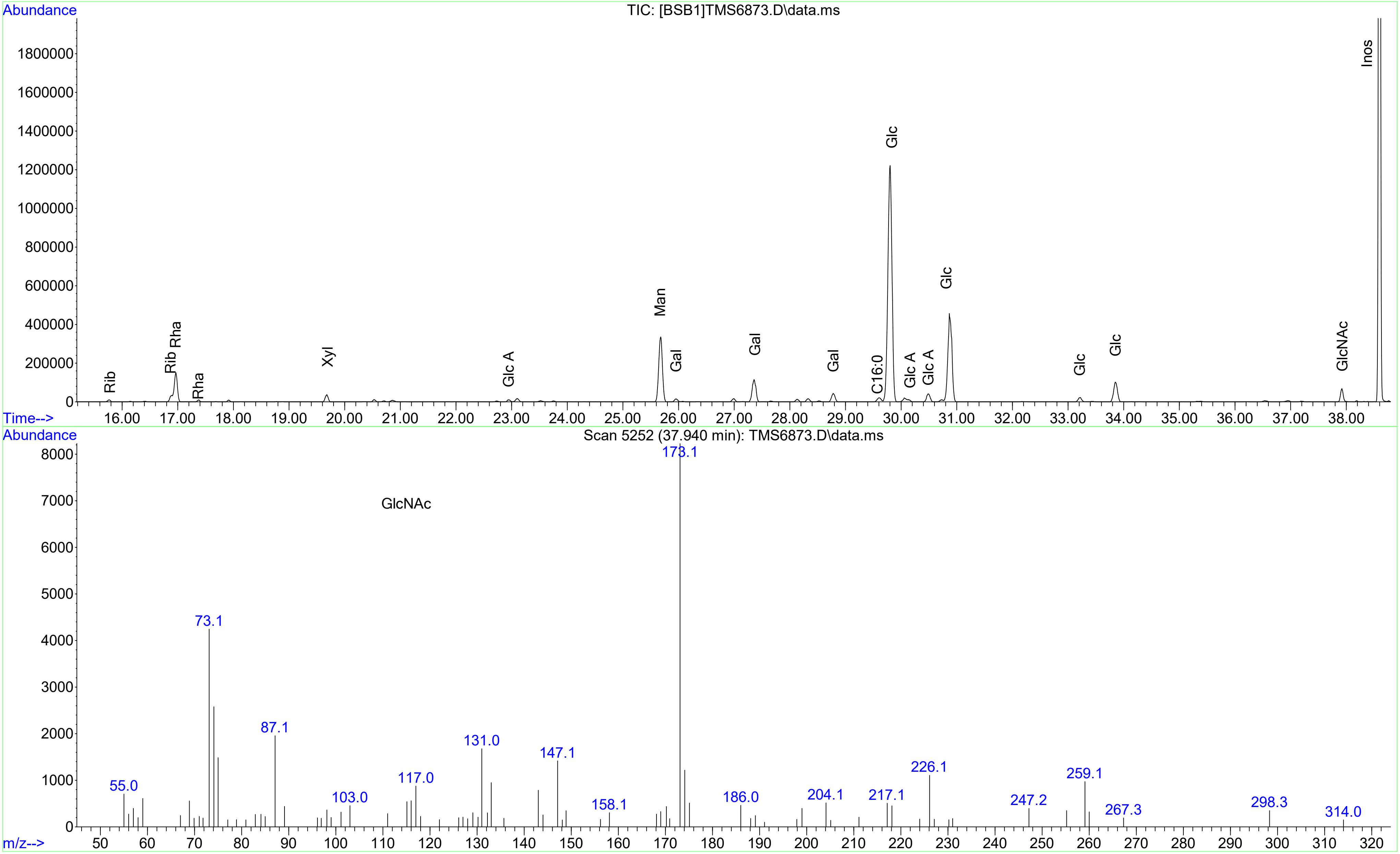

File :C:\Users\Ian\Desktop\BSB\TMS6873.D
Operator : [BSB1]Zhirui
Acquired : 17 Apr 2018 22:47 using AcqMethod TMS-EC1-1UL.M
Instrument : GC-TMS
Sample Name: KD
Misc Info :
Vial Number: 6

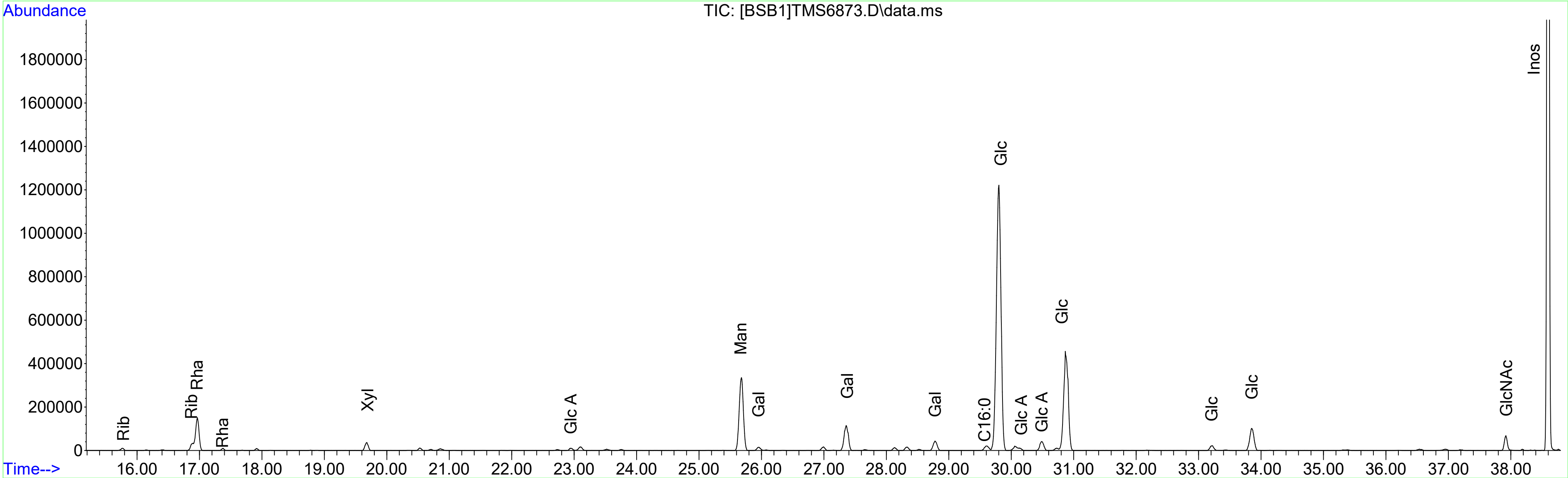


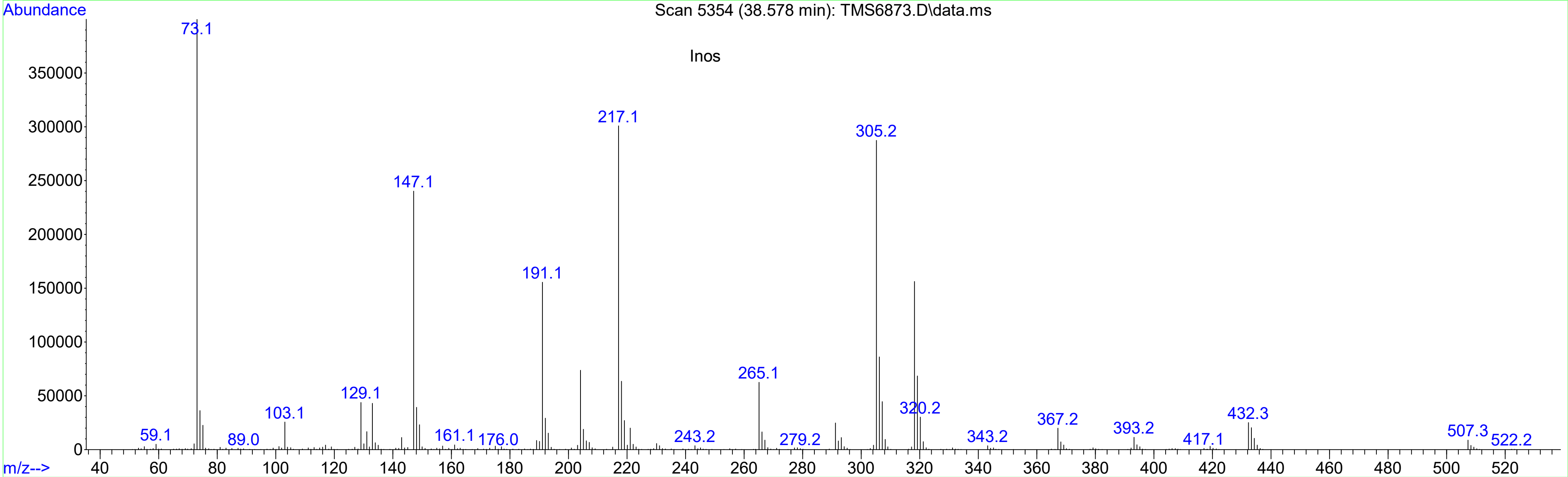

Supplement: Supplementary file 1 [file Data_Sheet_1.PDF]
