# Supplementary material for: Modulation of NKG2D, KIR2DL and Cytokine Production by Pleurotus ostreatus Glucan Enhances Natural Killer Cell Cytotoxicity Toward Cancer Cells
Source: Front Cell Dev Biol. 2019 Aug 13;7:165. doi: 10.3389/fcell.2019.00165 (PMC6700253; doi:10.3389/fcell.2019.00165)

File :C:\Users\Ian\Desktop\BSB\TMS6897.D
Operator : [BSB1]Khadar
Acquired : 23 Apr 2018 12:14 using AcqMethod AMINOZHIRUI.M
Instrument : GC-TMS
Sample Name: DW Link
Misc Info :
Vial Number: 1

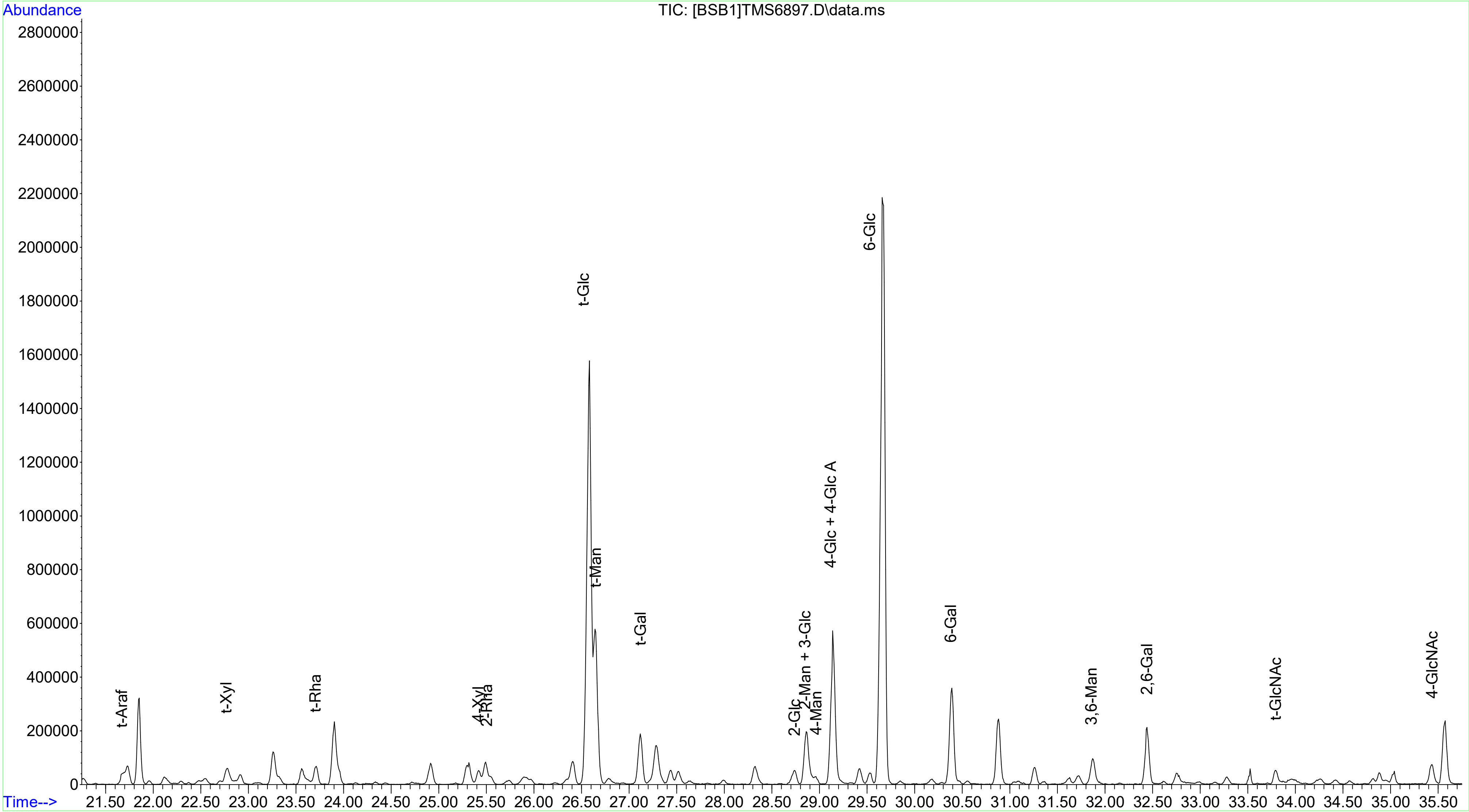

File   :C:\Users\Ian\Desktop\BSB\TMS6897.D
Operator   : [BSB1]Khadar
Acquired   : 23 Apr 2018 12:14   using AcqMethod AMINOZHIRUI.M
Instrument :   GC-TMS
Sample Name: DW Link
Misc Info :
Vial Number: 1

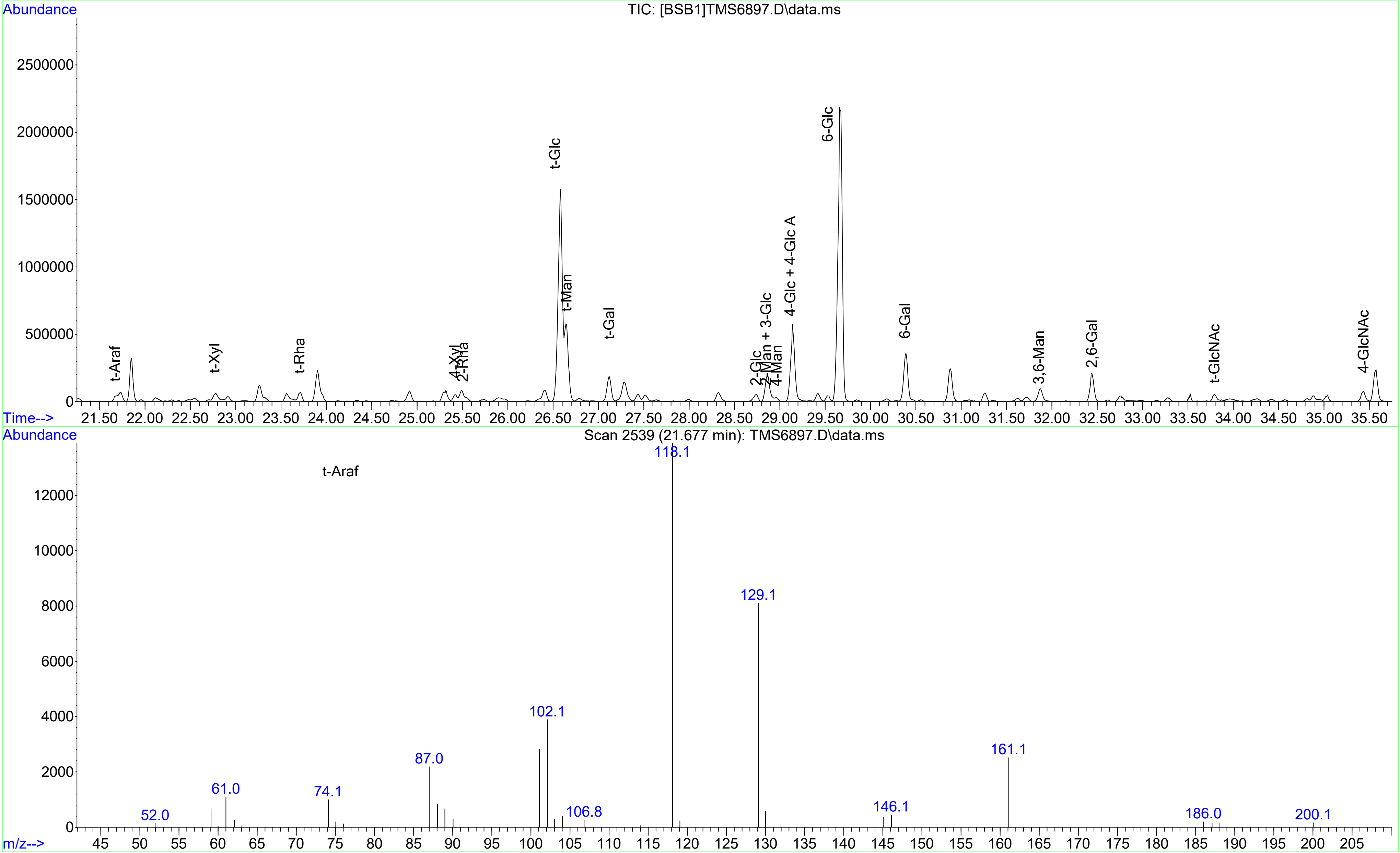

File :C:\Users\Ian\Desktop\BSB\TMS6897.D
Operator : [BSB1]Khadar
Acquired : 23 Apr 2018 12:14 using AcqMethod AMINOZHIRUI.M
Instrument : GC-TMS
Sample Name: DW Link
Misc Info :
Vial Number: 1

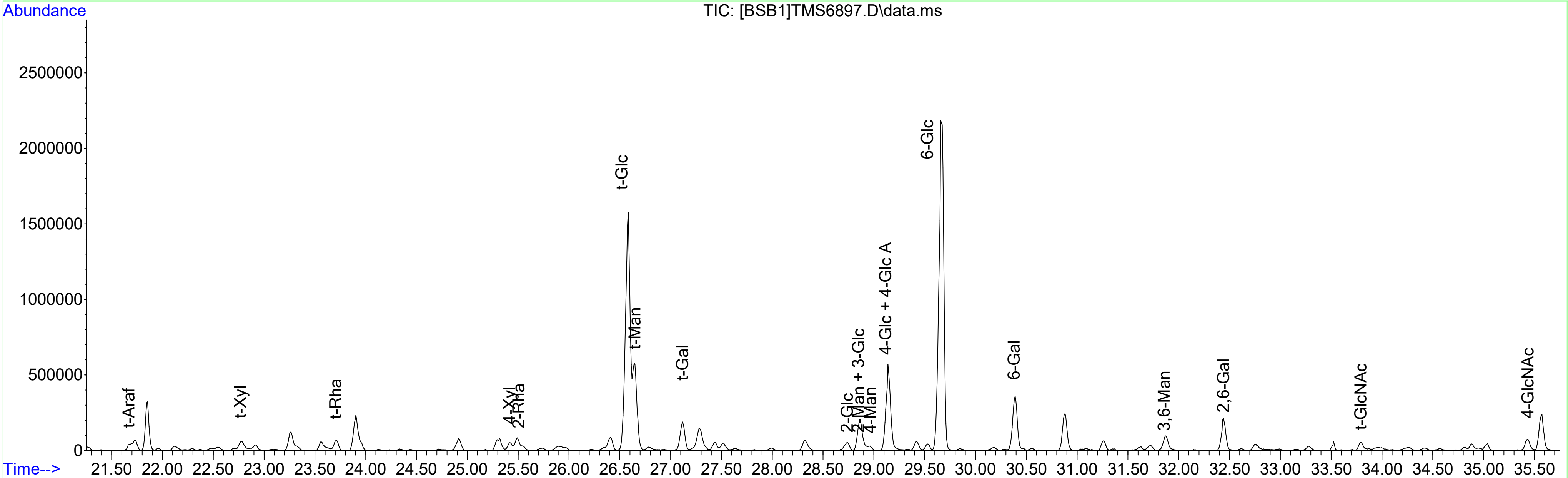

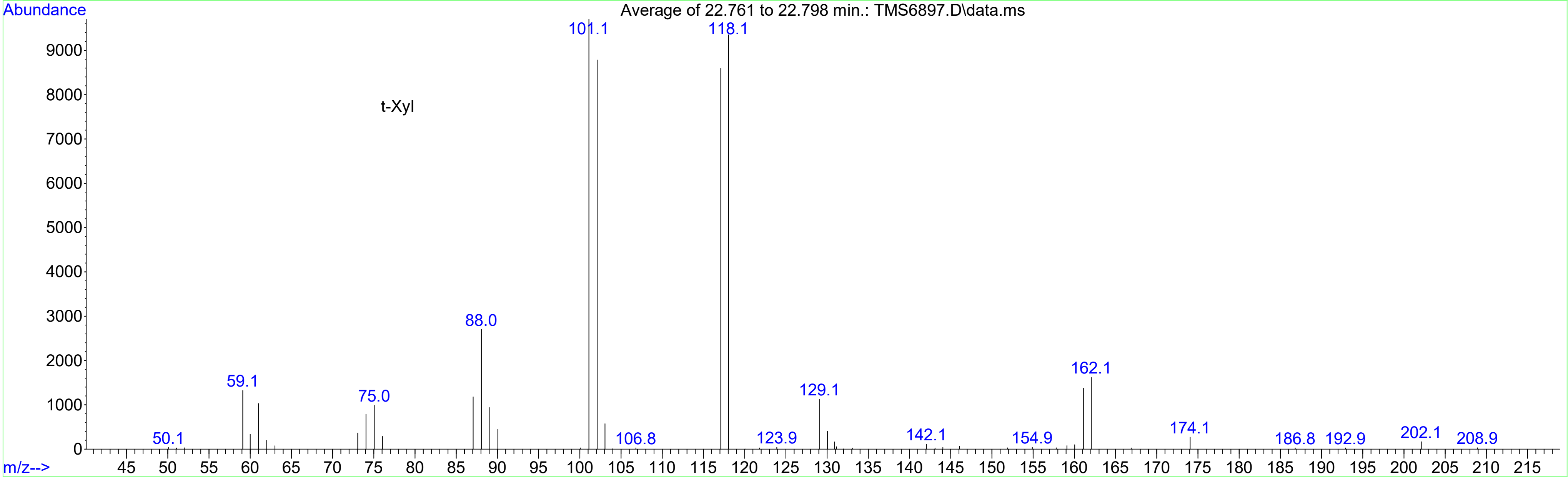

File :C:\Users\Ian\Desktop\BSB\TMS6897.D
Operator : [BSB1]Khadar
Acquired : 23 Apr 2018 12:14 using AcqMethod AMINOZHIRUI.M
Instrument : GC-TMS
Sample Name: DW Link
Misc Info :
Vial Number: 1

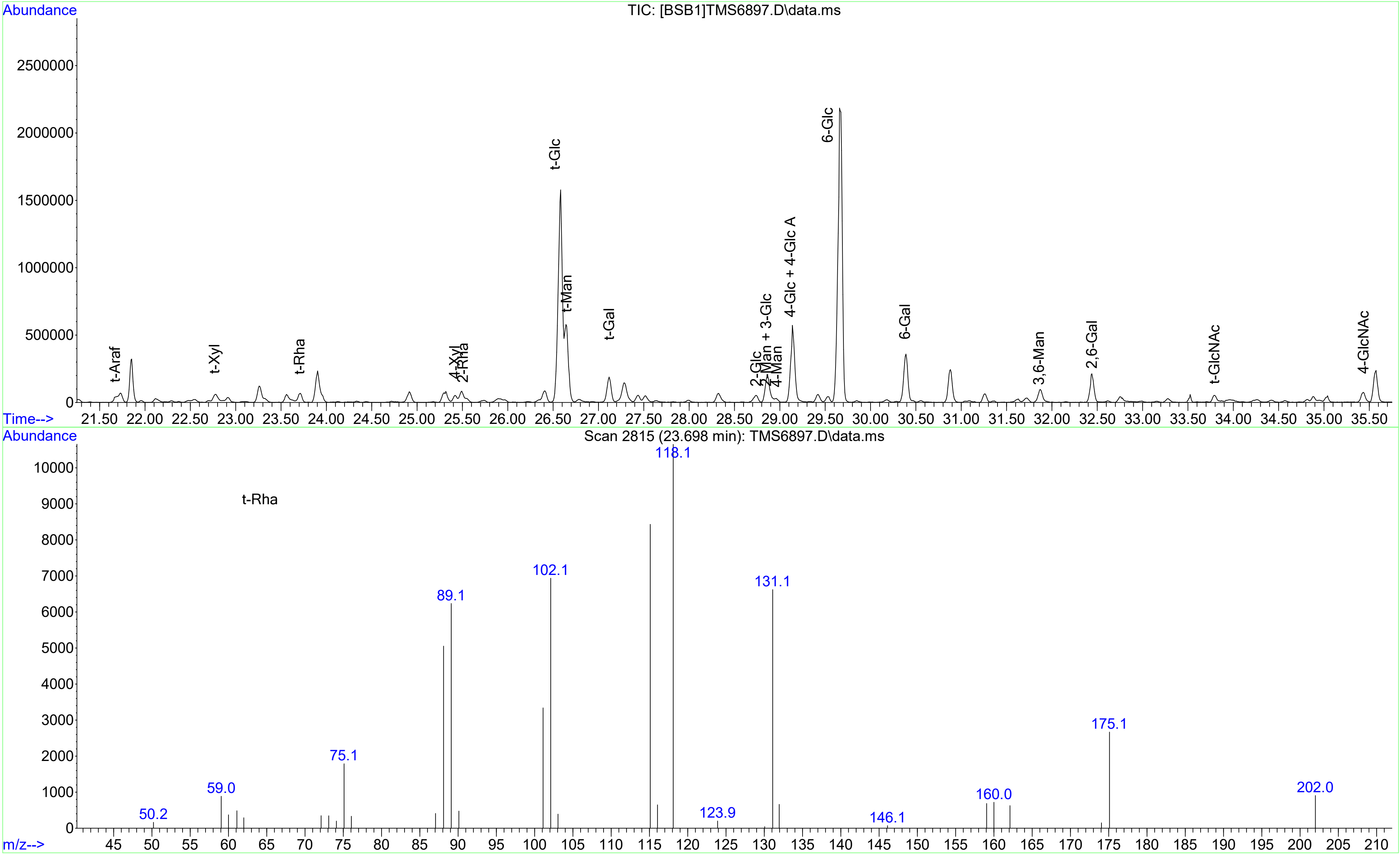

File :C:\Users\Ian\Desktop\BSB\TMS6897.D
Operator : [BSB1]Khadar
Acquired : 23 Apr 2018 12:14 using AcqMethod AMINOZHIRUI.M
Instrument : GC-TMS
Sample Name: DW Link
Misc Info :
Vial Number: 1

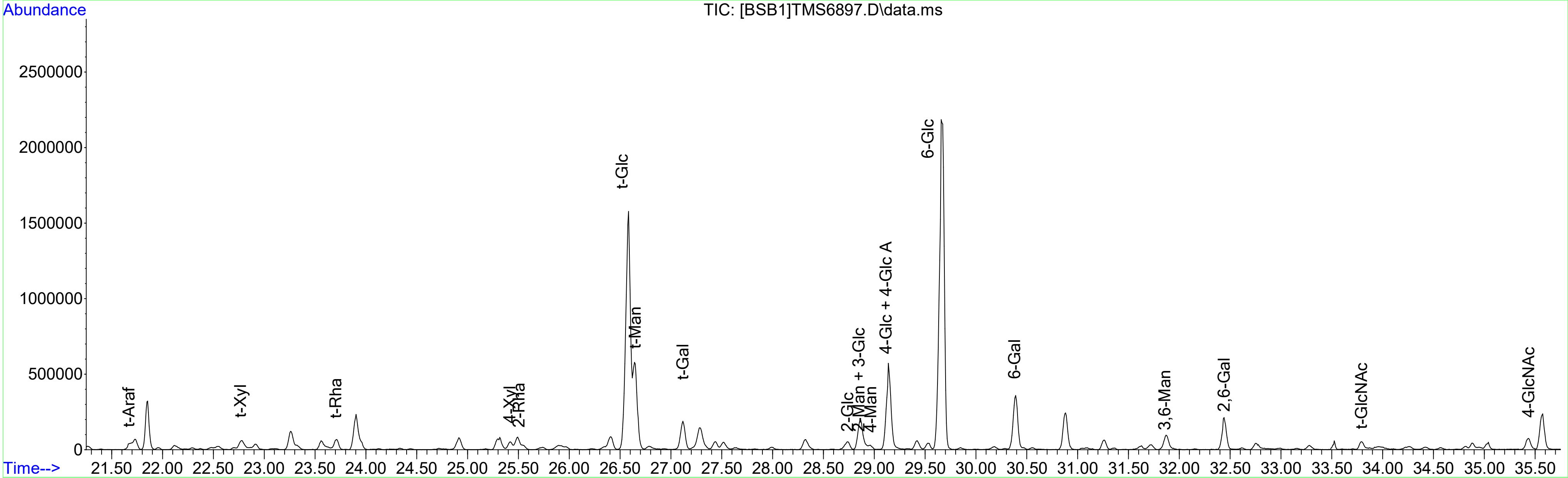

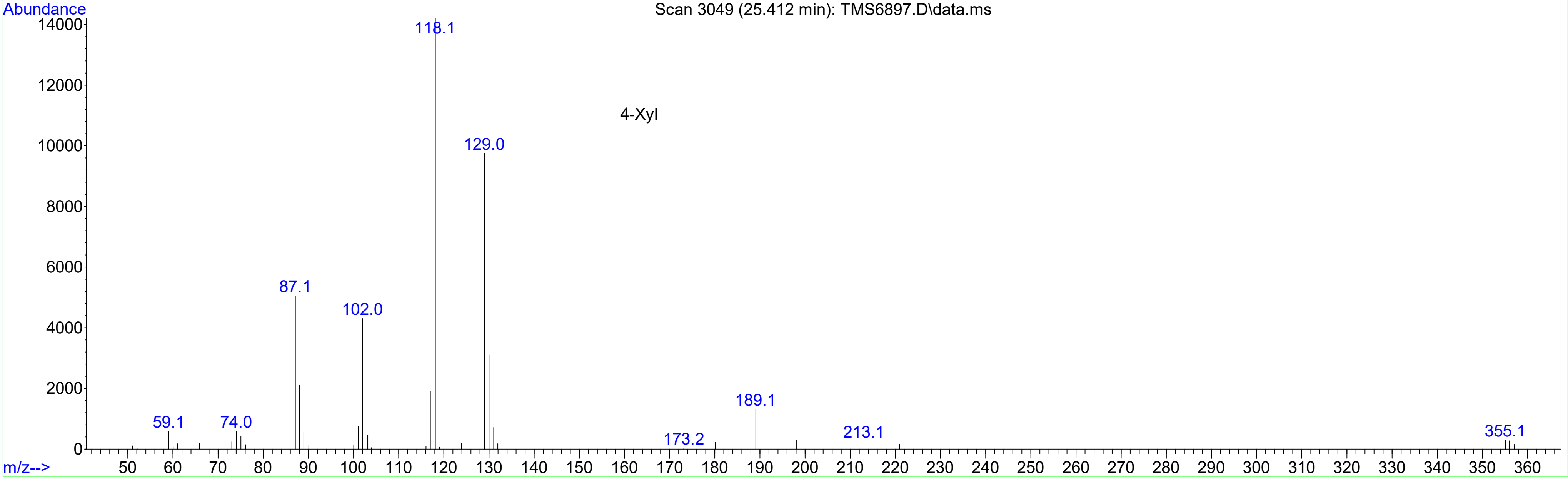

File :C:\Users\Ian\Desktop\BSB\TMS6897.D
Operator : [BSB1]Khadar
Acquired : 23 Apr 2018 12:14 using AcqMethod AMINOZHIRUI.M
Instrument : GC-TMS
Sample Name: DW Link
Misc Info :
Vial Number: 1

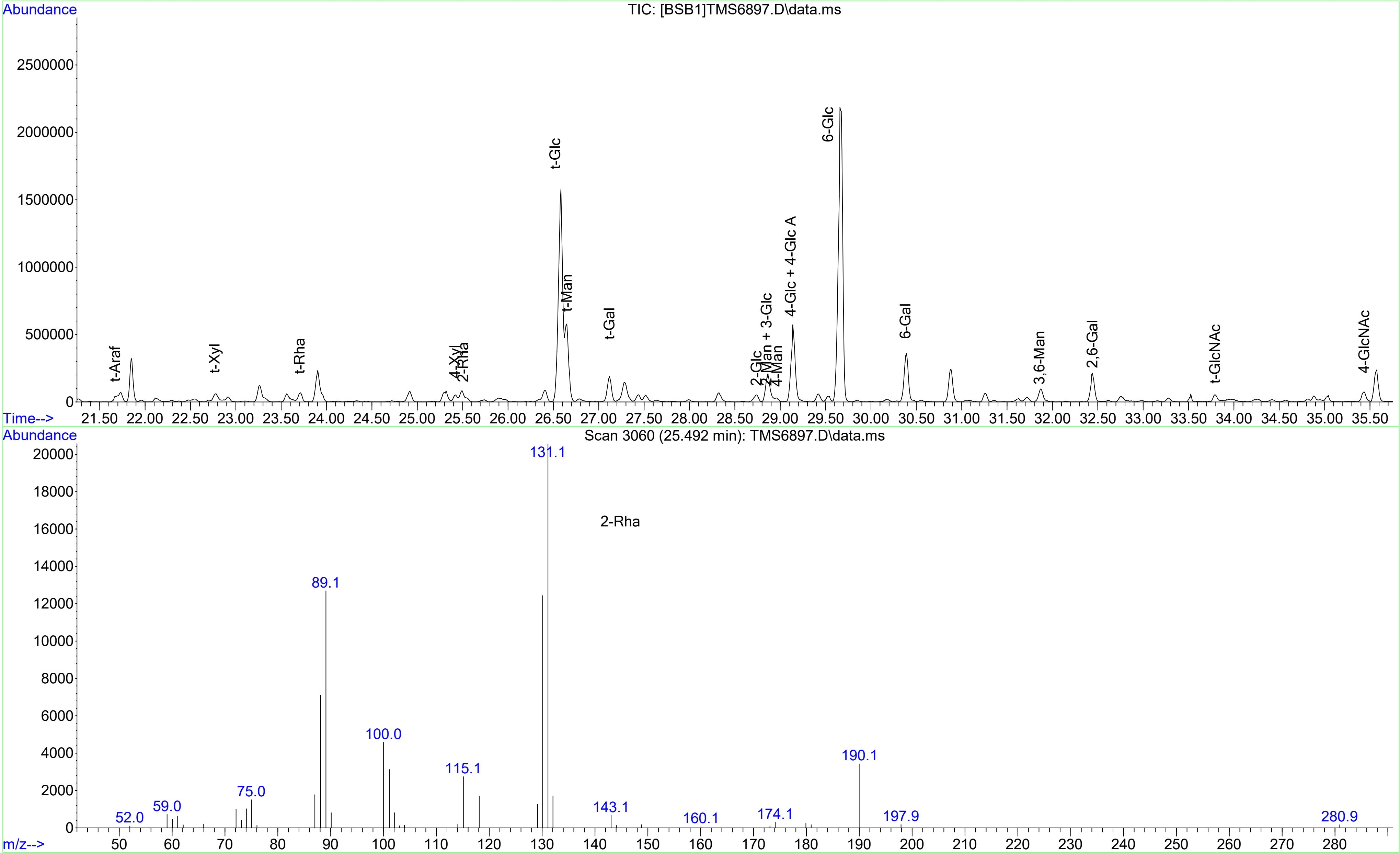

File   :C:\Users\Ian\Desktop\BSB\TMS6897.D
Operator   : [BSB1]Khadar
Acquired   : 23 Apr 2018 12:14   using AcqMethod AMINOZHIRUI.M
Instrument :   GC-TMS
Sample Name: DW Link
Misc Info :
Vial Number: 1

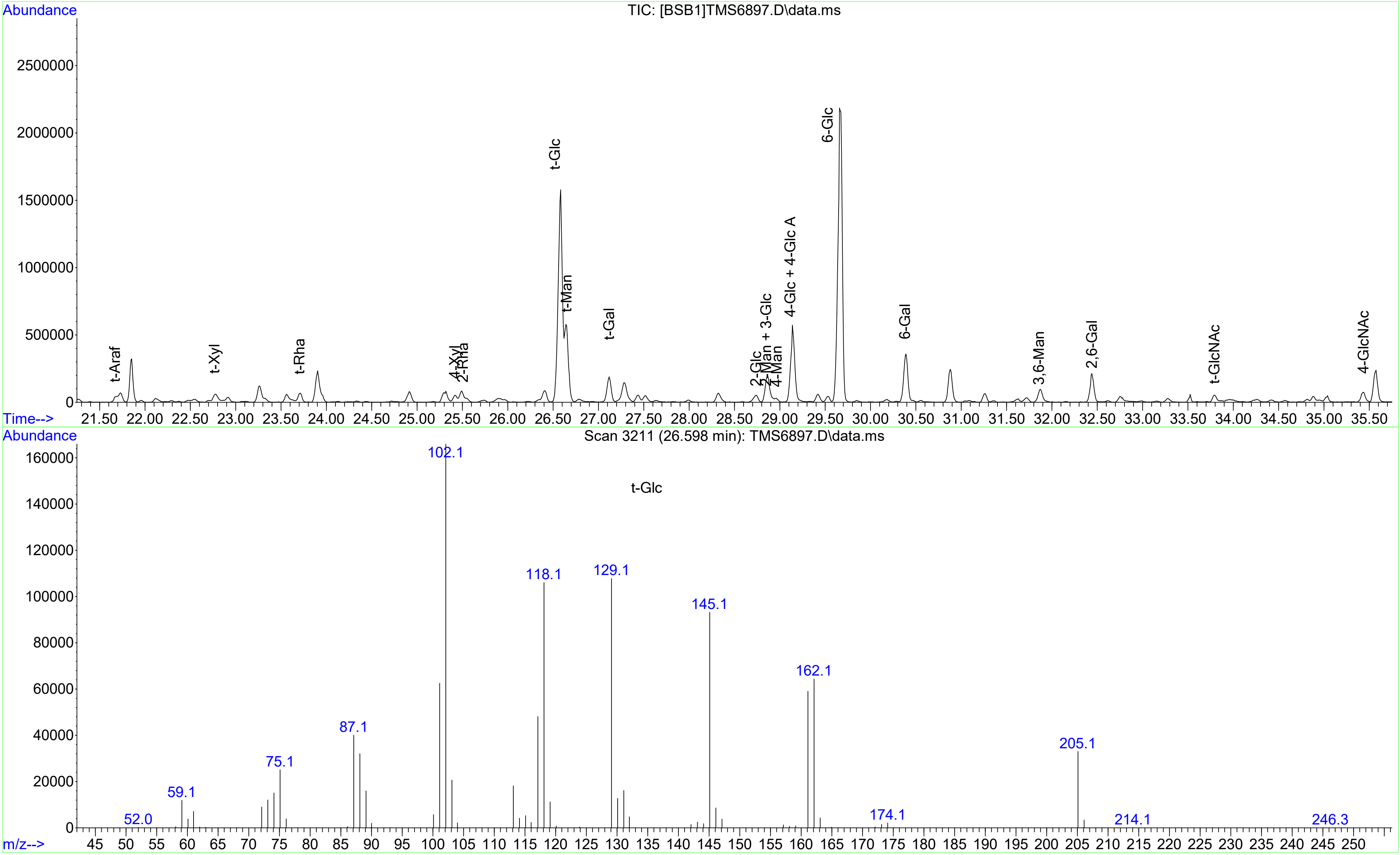

File :C:\Users\Ian\Desktop\BSB\TMS6897.D
Operator : [BSB1]Khadar
Acquired : 23 Apr 2018 12:14 using AcqMethod AMINOZHIRUI.M
Instrument : GC-TMS
Sample Name: DW Link
Misc Info :
Vial Number: 1

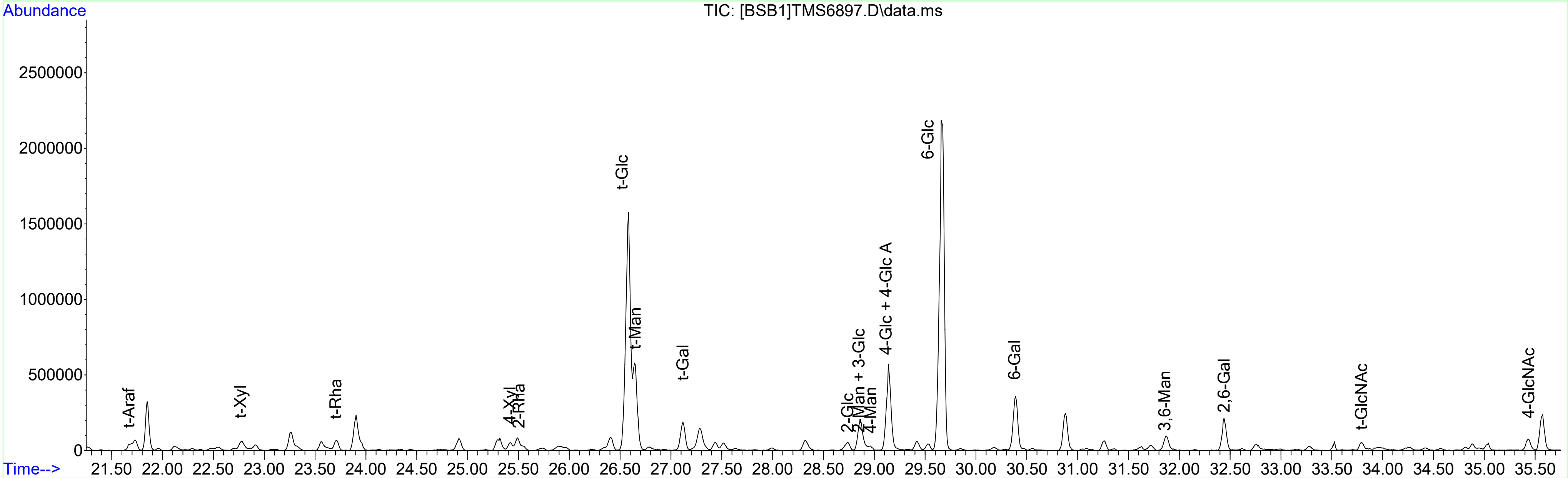

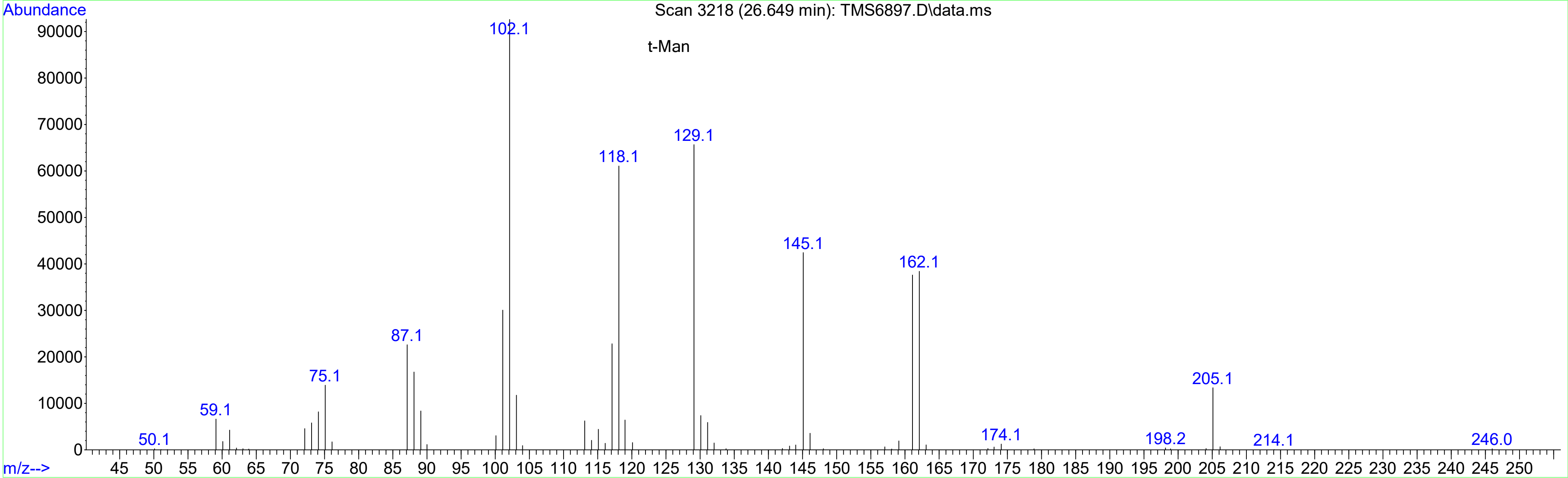

File :C:\Users\Ian\Desktop\BSB\TMS6897.D
Operator : [BSB1]Khadar
Acquired : 23 Apr 2018 12:14 using AcqMethod AMINOZHIRUI.M
Instrument : GC-TMS
Sample Name: DW Link
Misc Info :
Vial Number: 1

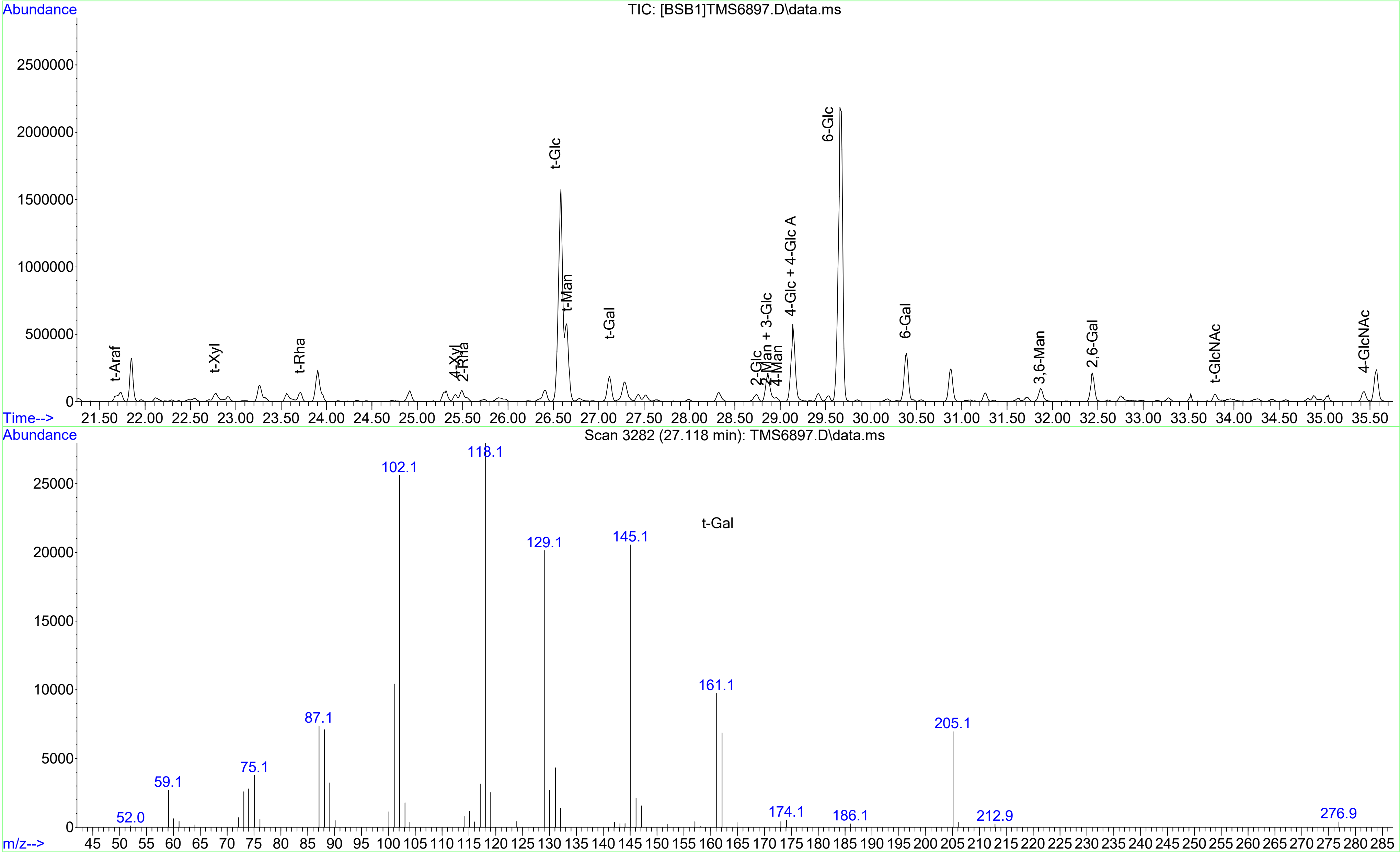

File :C:\Users\Ian\Desktop\BSB\TMS6897.D
Operator : [BSB1]Khadar
Acquired : 23 Apr 2018 12:14 using AcqMethod AMINOZHIRUI.M
Instrument : GC-TMS
Sample Name: DW Link
Misc Info :
Vial Number: 1

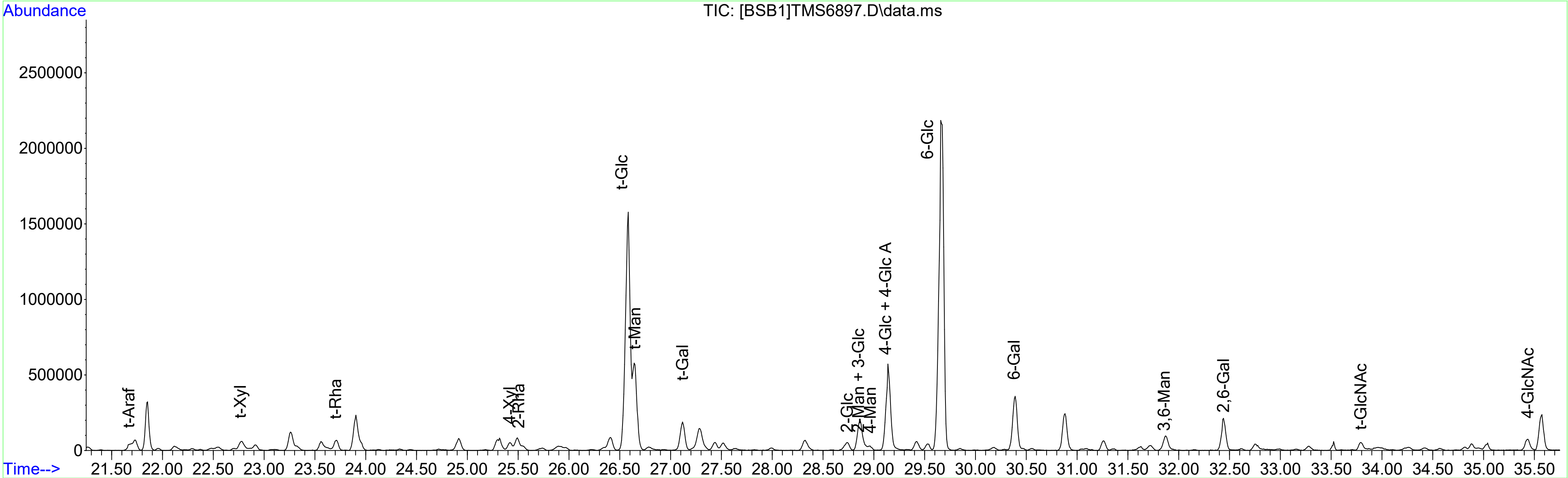

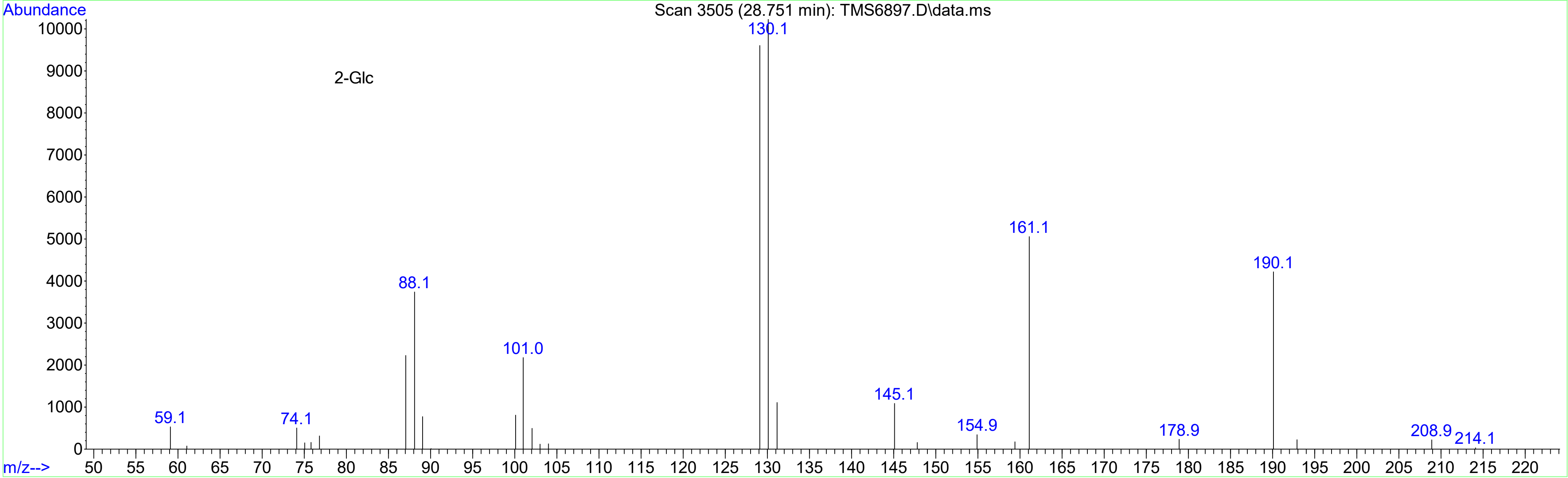

File :C:\Users\Ian\Desktop\BSB\TMS6897.D
Operator : [BSB1]Khadar
Acquired : 23 Apr 2018 12:14 using AcqMethod AMINOZHIRUI.M
Instrument : GC-TMS
Sample Name: DW Link
Misc Info :
Vial Number: 1

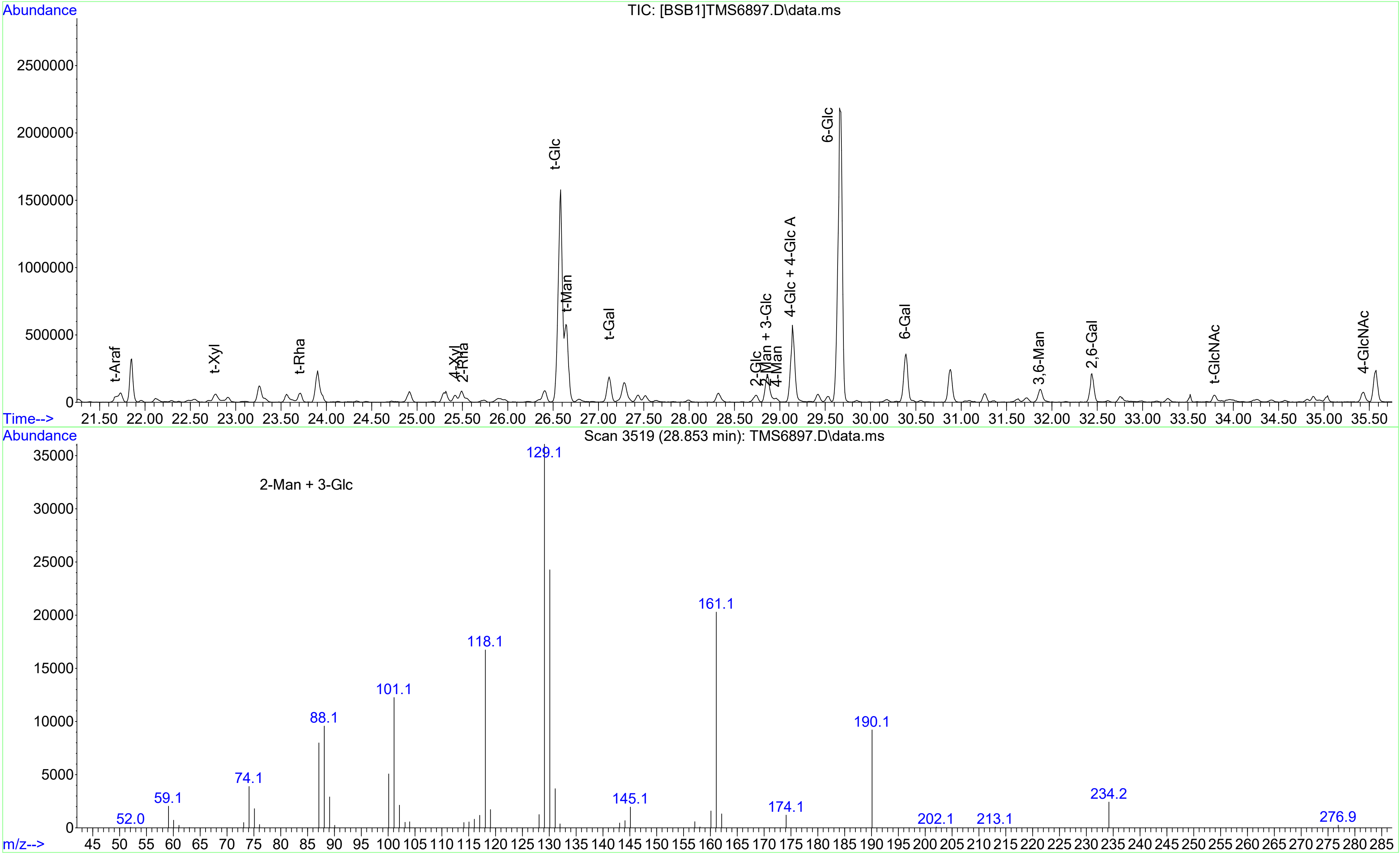

File :C:\Users\Ian\Desktop\BSB\TMS6897.D
Operator : [BSB1]Khadar
Acquired : 23 Apr 2018 12:14 using AcqMethod AMINOZHIRUI.M
Instrument : GC-TMS
Sample Name: DW Link
Misc Info :
Vial Number: 1

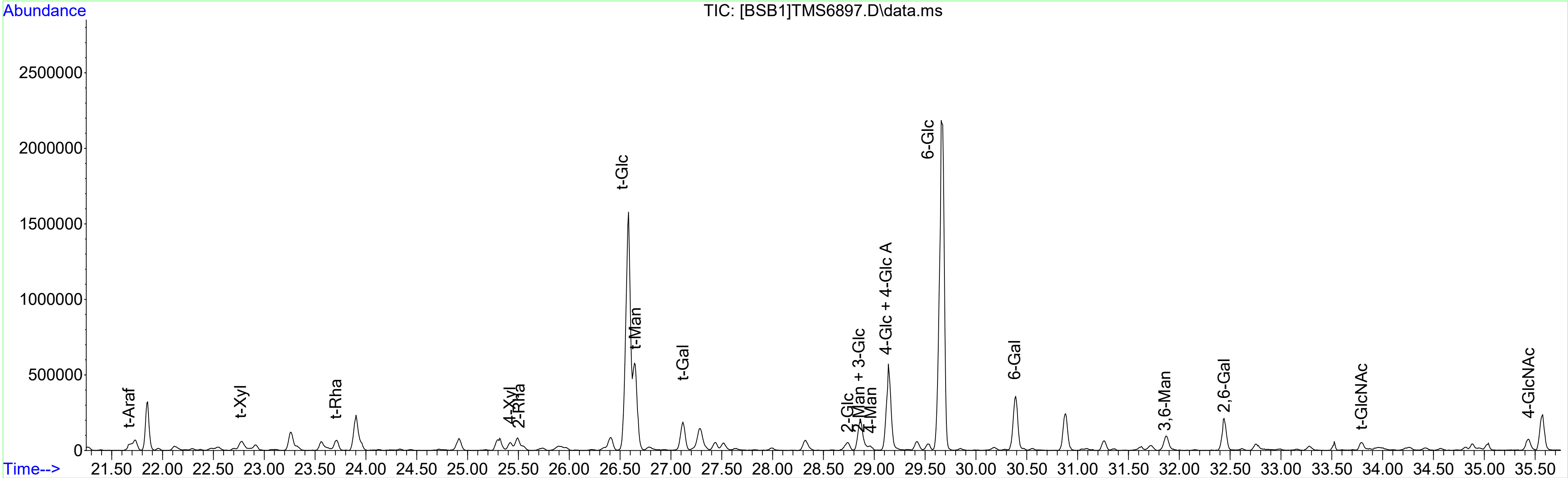

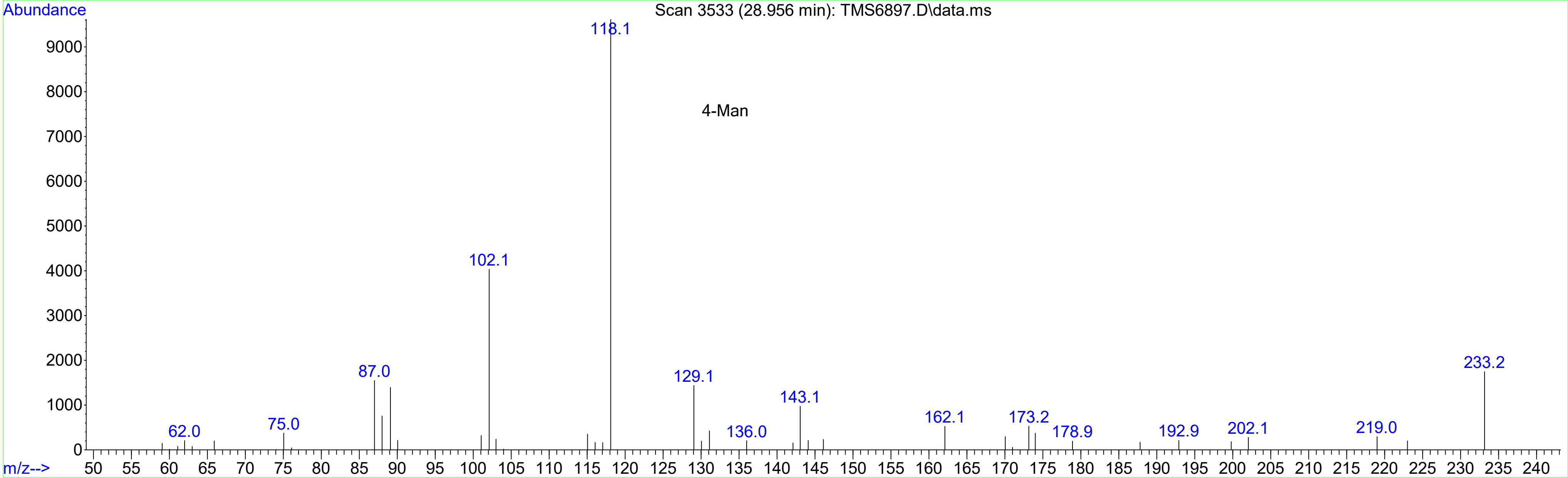

File :C:\Users\Ian\Desktop\BSB\TMS6897.D
Operator : [BSB1]Khadar
Acquired : 23 Apr 2018 12:14 using AcqMethod AMINOZHIRUI.M
Instrument : GC-TMS
Sample Name: DW Link
Misc Info :
Vial Number: 1

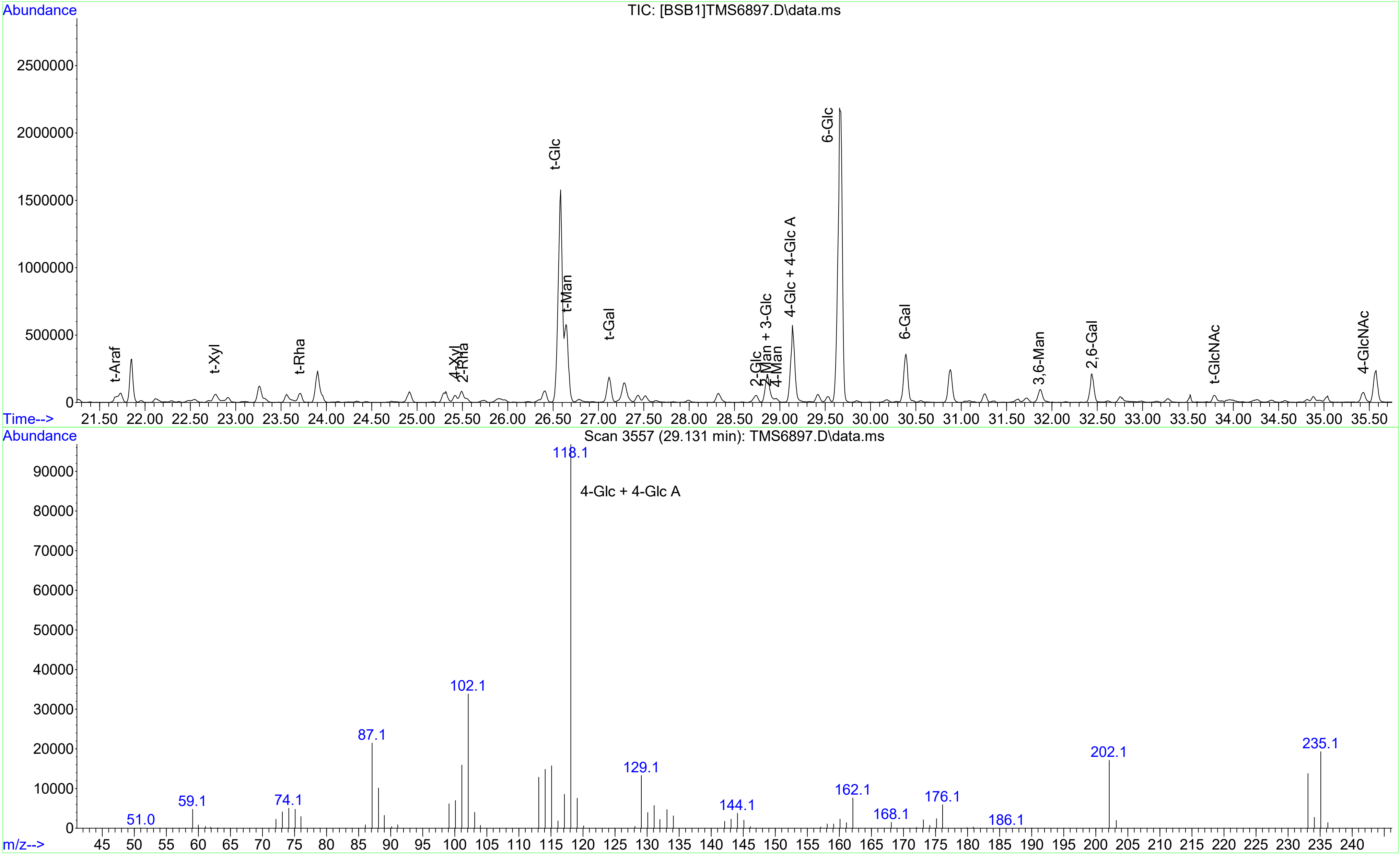

File :C:\Users\Ian\Desktop\BSB\TMS6897.D
Operator : [BSB1]Khadar
Acquired : 23 Apr 2018 12:14 using AcqMethod AMINOZHIRUI.M
Instrument : GC-TMS
Sample Name: DW Link
Misc Info :
Vial Number: 1

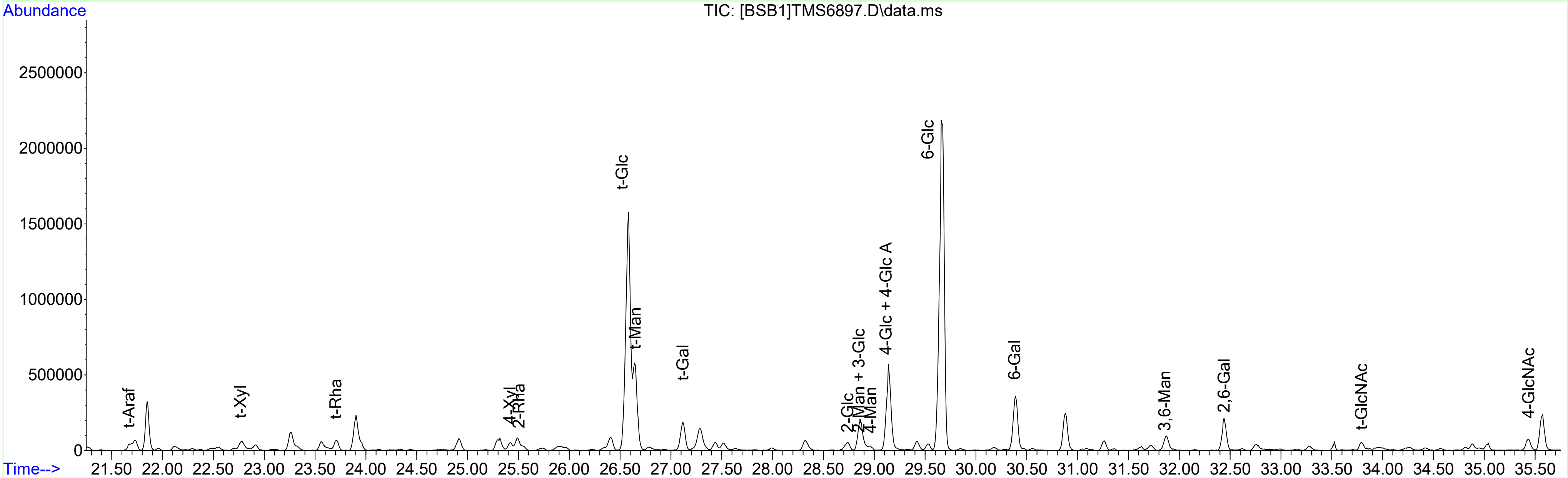

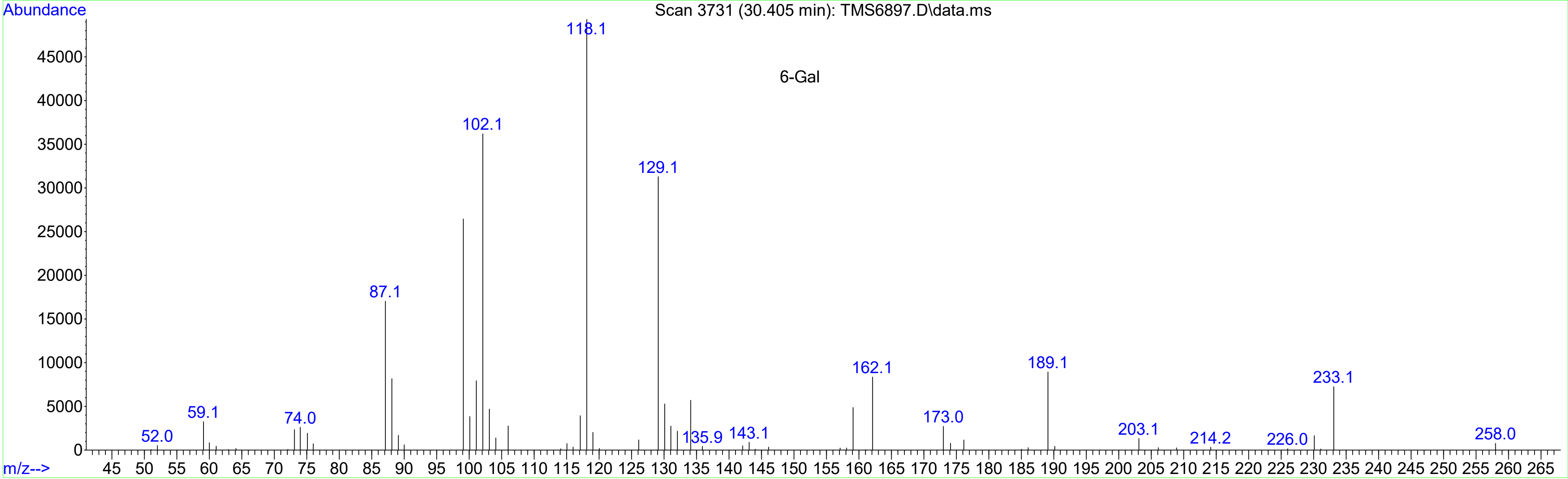

File :C:\Users\Ian\Desktop\BSB\TMS6897.D
Operator : [BSB1]Khadar
Acquired : 23 Apr 2018 12:14 using AcqMethod AMINOZHIRUI.M
Instrument : GC-TMS
Sample Name: DW Link
Misc Info :
Vial Number: 1

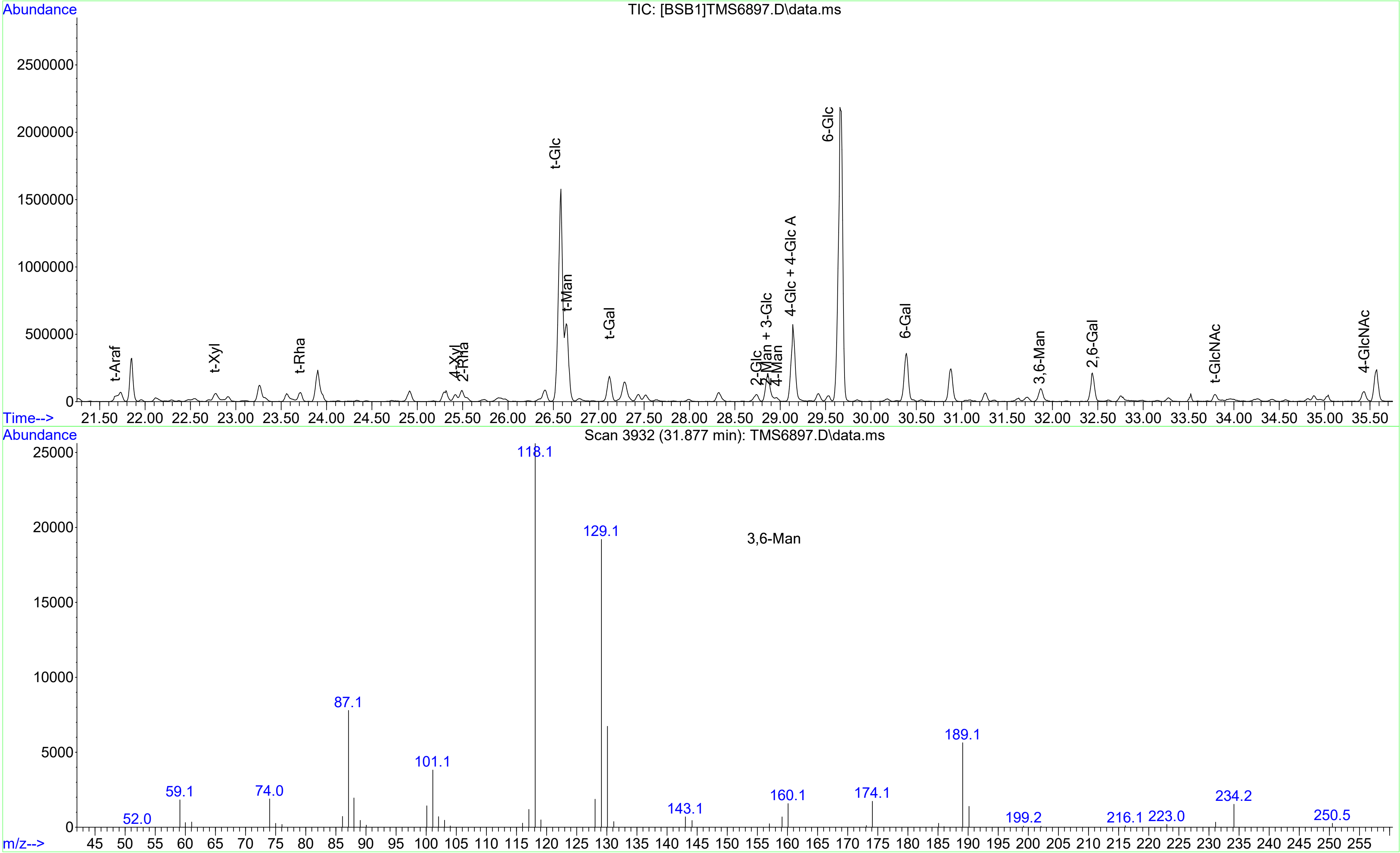

File   :C:\Users\Ian\Desktop\BSB\TMS6897.D
Operator   : [BSB1]Khadar
Acquired   : 23 Apr 2018 12:14   using AcqMethod AMINOZHIRUI.M
Instrument :   GC-TMS
Sample Name: DW Link
Misc Info :
Vial Number: 1

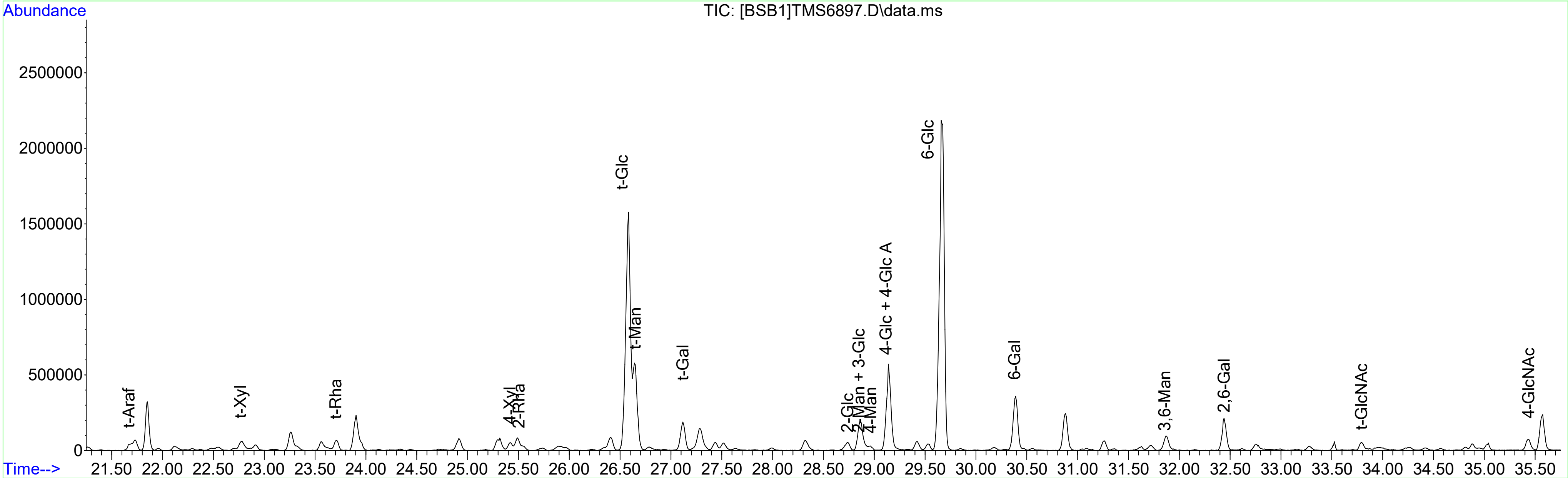

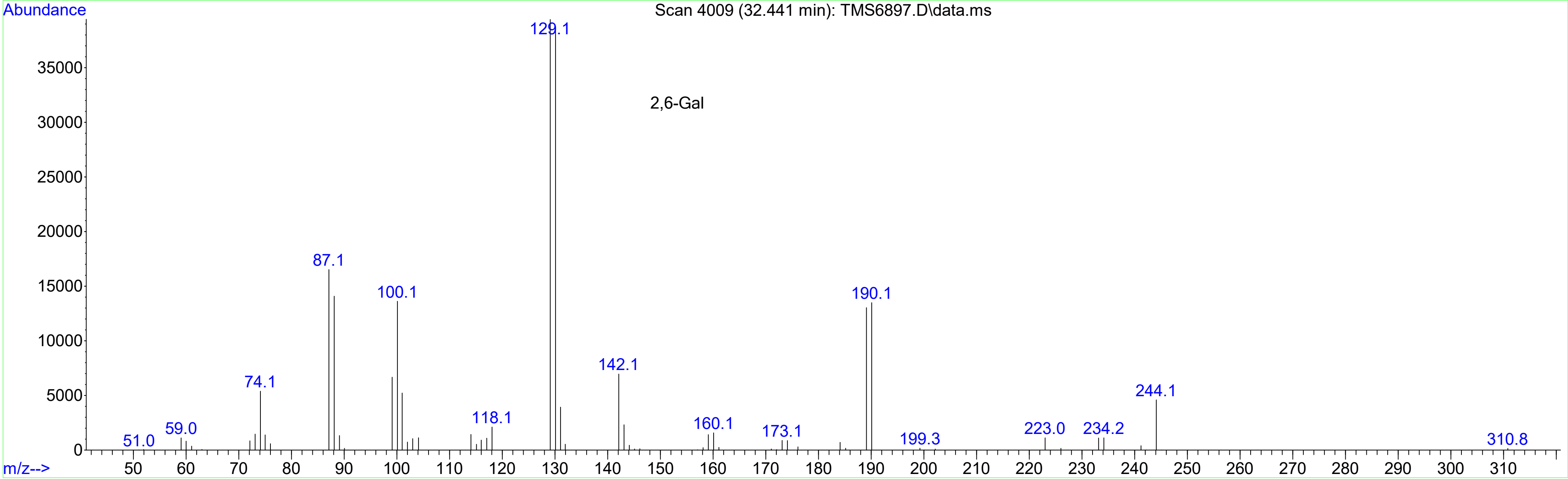

File :C:\Users\Ian\Desktop\BSB\TMS6897.D
Operator : [BSB1]Khadar
Acquired : 23 Apr 2018 12:14 using AcqMethod AMINOZHIRUI.M
Instrument : GC-TMS
Sample Name: DW Link
Misc Info :
Vial Number: 1

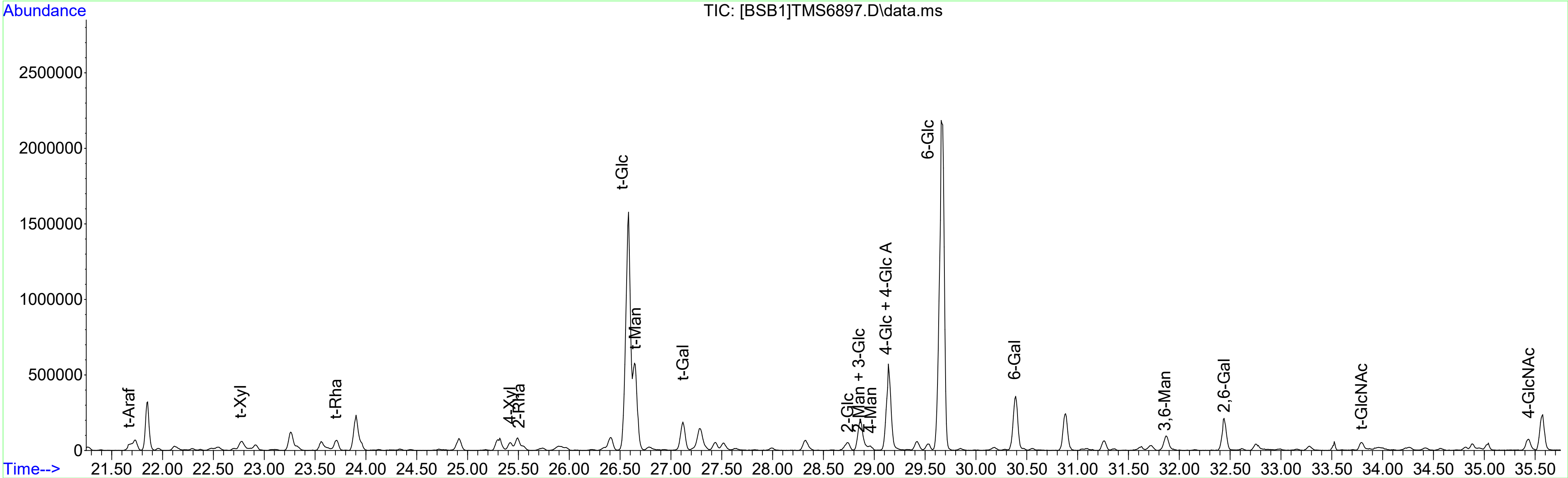

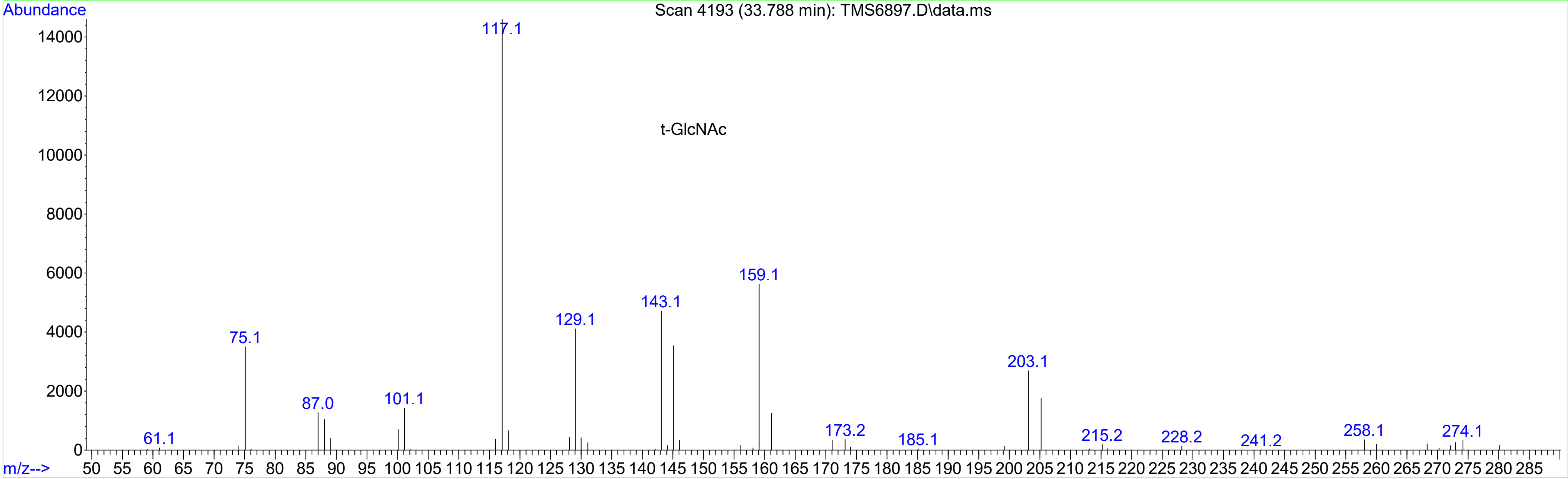

File :C:\Users\Ian\Desktop\BSB\TMS6897.D
Operator : [BSB1]Khadar
Acquired : 23 Apr 2018 12:14 using AcqMethod AMINOZHIRUI.M
Instrument : GC-TMS
Sample Name: DW Link
Misc Info :
Vial Number: 1

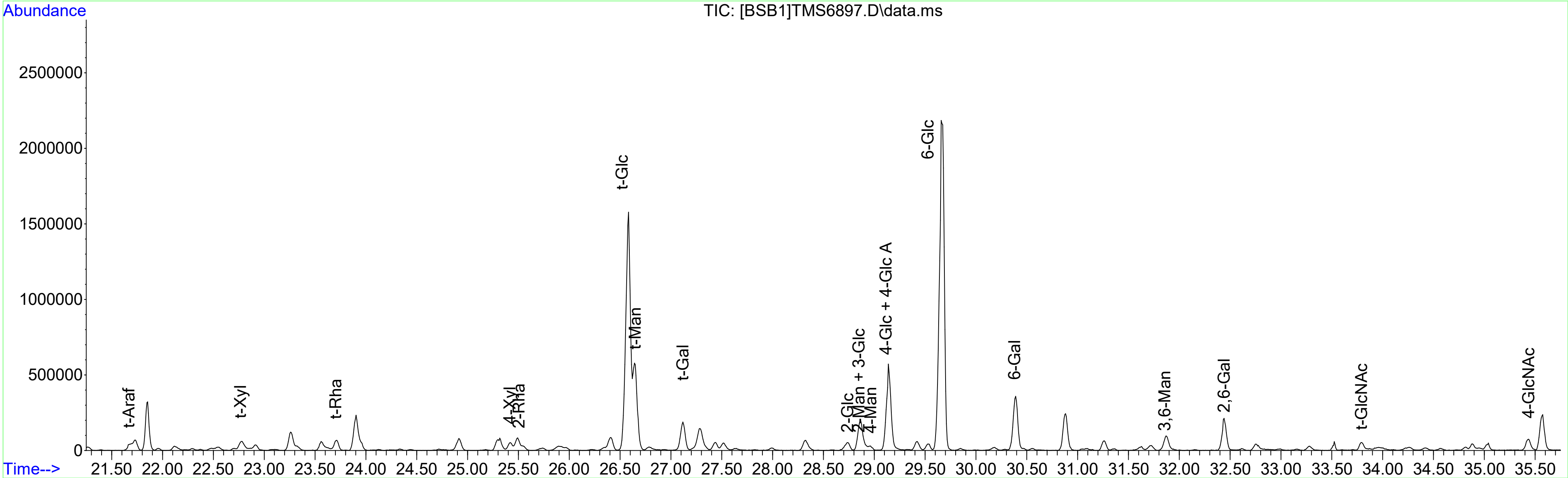

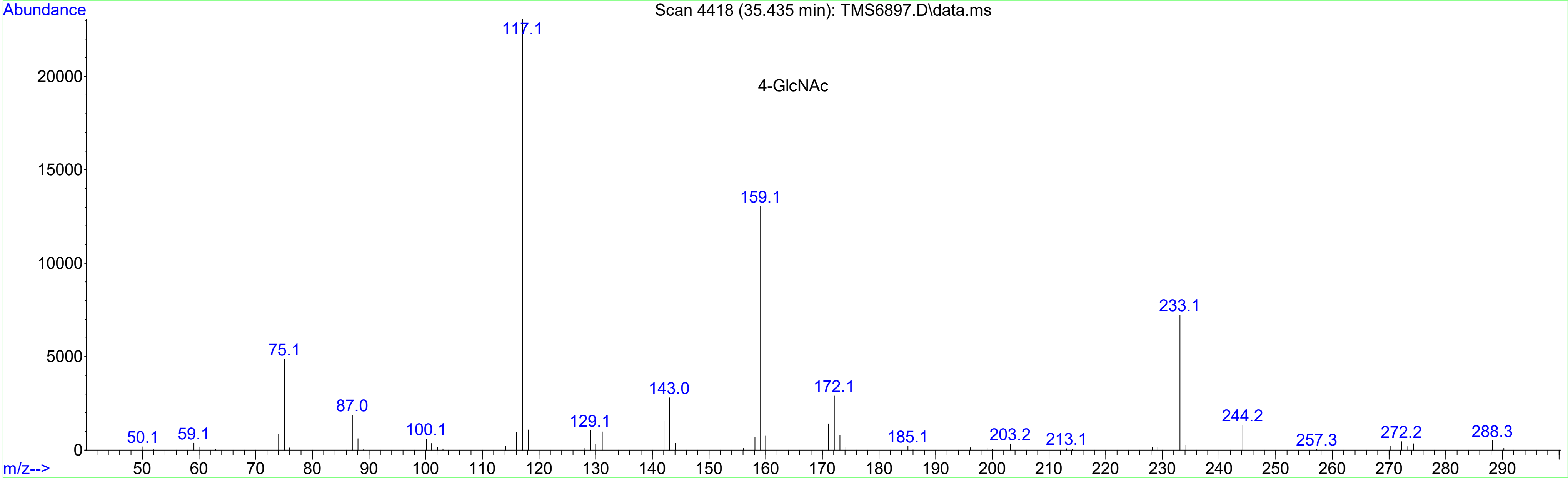

Supplement: Supplementary file 2 [file Data_Sheet_2.PDF]
