# Supplementary material for: Modulation of NKG2D, KIR2DL and Cytokine Production by Pleurotus ostreatus Glucan Enhances Natural Killer Cell Cytotoxicity Toward Cancer Cells
Source: Front Cell Dev Biol. 2019 Aug 13;7:165. doi: 10.3389/fcell.2019.00165 (PMC6700253; doi:10.3389/fcell.2019.00165)
Supplement: Supplementary file 3 [file Data_Sheet_3.PDF]

Table 1s: HLA typing from the cancer cell lines

Cell line	Disease	HLA-A	HLA-B	HLA-C
MCF7	breast ductal carcinoma	**A*02:01/A*02:01**	**B*44:02/B*18:01**	**C*05:01/C*05:01**
A549	Lung carcinoma	**A*25:01**	**A*30:01**	**B*44:03**
HepG2	Hepatocellular carcinoma	**A*02:01,24:02**	**B*51:01,35:14**	**C*16:02,04:01**

Table 2s: List of primers used

List	Sequences
NKG2D	Forward 5'-TTC AAC ACG ATG GCA AAA GC-3'
	Reverse 5'-CTA CAG CGA TGA AGC AGC AGA-3'
KIR2DL4R	Forward 5′ GTC ACT CGG GTC TGA CCA CT 3′
	Reverse 5′ TCT CCA TCA GTGC GCA TGA A/G 3′
Interferon-γ	Forward 5′CAG GTC ATT CAG ATG TAG CGG ATA 3′
	Reverse 5′AGG AGA CAA TTT GGC TCT GCA TT 3′
β actin	Forward 5′- GTGGGGCGCCCCAGGCACCA-3′
	Reverse 3′- CTCCTTAATGTCACGCACGATTTC -3′

Table 3s: Quantification of the activation markers of NK cells in NK-MCF7 cells model using RTqPCR

Target	Sample	Mean Cq	Mean Efficiency Corrected Cq	Normalized Expression	Relative Normalized Expression	Regulation	Compared to Regulation Threshold
Actin	control	20.73	20.73				No change
Actin	crude	22.01	22.01				No change
Actin	F1	20.97	20.97				No change
Actin	F2	23.22	23.22				No change
Actin	F3	21.31	21.31				No change
INF	control	23.44	23.44	0.15260	1.00000	1.00000	No change
INF	crude	24.46	24.46	0.18328	1.20102	1.20102	No change
INF	F1	24.84	24.84	0.06833	0.44776	-2.23336	No change
INF	F2	24.51	24.51	0.40915	2.68115	2.68115	No change
INF	F3	25.05	25.05	0.07508	0.49196	-2.03268	No change
KIR2DL	control	12.71	12.71	259.85633	1.00000	1.00000	No change
KIR2DL	crude	21.19	21.19	1.76397	0.00679	-147.31295	Down regulated
KIR2DL	F1	24.26	24.26	0.10226	0.00039	-2541.09605	Down regulated
KIR2DL	F2	24.38	24.38	0.44806	0.00172	-579.95249	Down regulated
KIR2DL	F3	20.53	20.53	1.72067	0.00662	-151.02052	Down regulated
NKG2D	control	18.77	18.77	3.88961	1.00000	1.00000	No change
NKG2D	crude	13.96	13.96	264.28112	67.94539	67.94539	Up regulated
NKG2D	F1	18.30	18.30	6.33338	1.62828	1.62828	No change
NKG2D	F2	14.81	14.81	339.06976	87.17319	87.17319	Up regulated
NKG2D	F3	15.31	15.31	64.42737	16.56396	16.56396	Up regulated

Table 4s: Quantification of the activation markers of NK cells in NK-MCF7 cells model in the presence of IL2 using RTqPCR

Target	Sample	Mean Cq	Mean Efficiency Corrected Cq	Normalized Expression	Relative Normalized Expression	Regulation	Compared to Regulation Threshold	P-Value	Exceeds P-Value Threshold
Actin	control	6.04	6.04				No change		No
Actin	crude	22.56	22.56				No change		No
Actin	F1	2.80	2.80				No change		No
Actin	F2	22.18	22.18				No change		No
Actin	F3	16.79	16.79				No change		No
INF	control	5.29	5.29	1.68142	1.00000	1.00000	No change		No
INF	crude	19.69	19.69	7.29213	4.33689	4.33689	Up regulated		No
INF	F1	22.15	22.15	0.00000	0.00000	-1120423.97747	Down regulated		No
INF	F2	3.41	3.41	444932.33436	264617.21177	264617.21177	Up regulated		No
INF	F3	5.86	5.86	1947.67902	1158.35455	1158.35455	Up regulated		No
KIR2DL	control	21.19	21.19	0.00003	1.00000	1.00000	No change		No
KIR2DL	crude	20.22	20.22	5.05708	183997.83537	183997.83537	Up regulated		No
KIR2DL	F1	5.94	5.94	0.11391	4144.56043	4144.56043	Up regulated		No
KIR2DL	F2	12.96	12.96	595.58791	21670004.36610	21670004.36610	Up regulated		No
KIR2DL	F3	22.15	22.15	0.02428	883.26881	883.26881	Up regulated		No
NKG2D	control	12.72	12.72	0.00977	1.00000	1.00000	No change		No
NKG2D	crude	14.04	14.04	366.83150	37541.65225	37541.65225	Up regulated		No
NKG2D	F1	16.10	16.10	0.00010	0.01013	-98.71793	Down regulated		No
NKG2D	F2	14.79	14.79	167.24997	17116.41492	17116.41492	Up regulated		No
NKG2D	F3	15.36	15.36	2.68977	275.27218	275.27218	Up regulated		No

Table 5s: Quantification of the activation markers of NK cells in NK- A549 cells model using RTqPCR

Target	Sample	Mean Cq	Mean Efficiency Corrected Cq	Normalized Expression	Relative Normalized Expression	Regulation	Compared to Regulation Threshold	P-Value	Exceeds P-Value Threshold
Actin	control	10.33	10.33				No change		No
Actin	crude	39.07	39.07				No change		No
Actin	F1	36.20	36.20				No change		No
Actin	F2	36.64	36.64				No change		No
Actin	F3	39.65	39.65				No change		No
INF	control	23.39	23.39	0.00012	1.00000	1.00000	No change		No
INF	crude	24.33	24.33	27417.48932	234817167.46315	234817167.46315	Up regulated		No
INF	F1	22.52	22.52	13078.26295	112008821.27438	112008821.27438	Up regulated		No
INF	F2	24.04	24.04	6206.99559	53159831.88345	53159831.88345	Up regulated		No
INF	F3	24.55	24.55	35094.14923	300563943.70796	300563943.70796	Up regulated		No
KIR2DL	control	21.30	21.30	0.00050	1.00000	1.00000	No change		No
KIR2DL	crude	23.29	23.29	56101.70765	112393062.74907	112393062.74907	Up regulated		No
KIR2DL	F1	33.18	33.18	8.07240	16172.09377	16172.09377	Up regulated		No
KIR2DL	F2	21.85	21.85	28401.90205	56899814.51991	56899814.51991	Up regulated		No
KIR2DL	F3	23.73	23.73	62319.80413	124850275.49201	124850275.49201	Up regulated		No
NKG2D	control	13.55	13.55	0.10705	1.00000	1.00000	No change		No
NKG2D	crude	27.70	27.70	2642.47301	24683.40273	24683.40273	Up regulated		No
NKG2D	F1	15.99	15.99	1210865.15176	11310719.93335	11310719.93335	Up regulated		No
NKG2D	F2	20.40	20.40	77752.00381	726283.30093	726283.30093	Up regulated		No
NKG2D	F3	15.13	15.13	24124816.04349	225350475.49593	225350475.49593	Up regulated		No

Table 6s: Quantification of the activation markers of NK cells in NK-A549 cells model in the presence of IL2 using RTqPCR

Target	Sample	Mean Cq	Mean Efficiency Corrected Cq	Normalized Expression	Relative Normalized Expression	Regulation	Compared to Regulation Threshold	P-Value	Exceeds P-Value Threshold
Actin	control	21.57	21.57				No change		No
Actin	crude	18.81	18.81				No change		No
Actin	F1	19.91	19.91				No change		No
Actin	F2	21.40	21.40				No change		No
Actin	F3	24.05	24.05				No change		No
INF	control	22.12	22.12	0.68169	1.00000	1.00000	No change		No
INF	crude	10.55	10.55	307.31460	450.81415	450.81415	Up regulated		No
INF	F1	6.43	6.43	11424.28875	16758.82285	16758.82	Up regulated		No
INF	F2	14.51	14.51	117.94804	173.02350	173.02350	Up regulated		No
INF	F3	1.59	1.59	5772106.25900	8467372.31486	8467372.3	Up regulated		No
KIR2DL	control	10.68	10.68	1894.30853	1.00000	1.00000	No change		No
KIR2DL	crude	7.90	7.90	1929.16414	1.01840	1.01840	No change		No
KIR2DL	F1	20.21	20.21	0.81152	0.00043	-2334.27	Down regulated		No
KIR2DL	F2	9.01	9.01	5335.80017	2.81675	2.81675	No change		No
KIR2DL	F3	20.42	20.42	12.35553	0.00652	-153.31660	Down regulated		No
NKG2D	control	16.26	16.26	39.69096	1.00000	1.00000	No change		No
NKG2D	crude	12.97	12.97	57.42774	1.44687	1.44687	No change		No
NKG2D	F1	16.11	16.11	13.90081	0.35023	-2.85530	No change		No
NKG2D	F2	15.42	15.42	62.90706	1.58492	1.58492	No change		No
NKG2D	F3	9.31	9.31	27275.08862	687.18649	687.18649	Up regulated		No

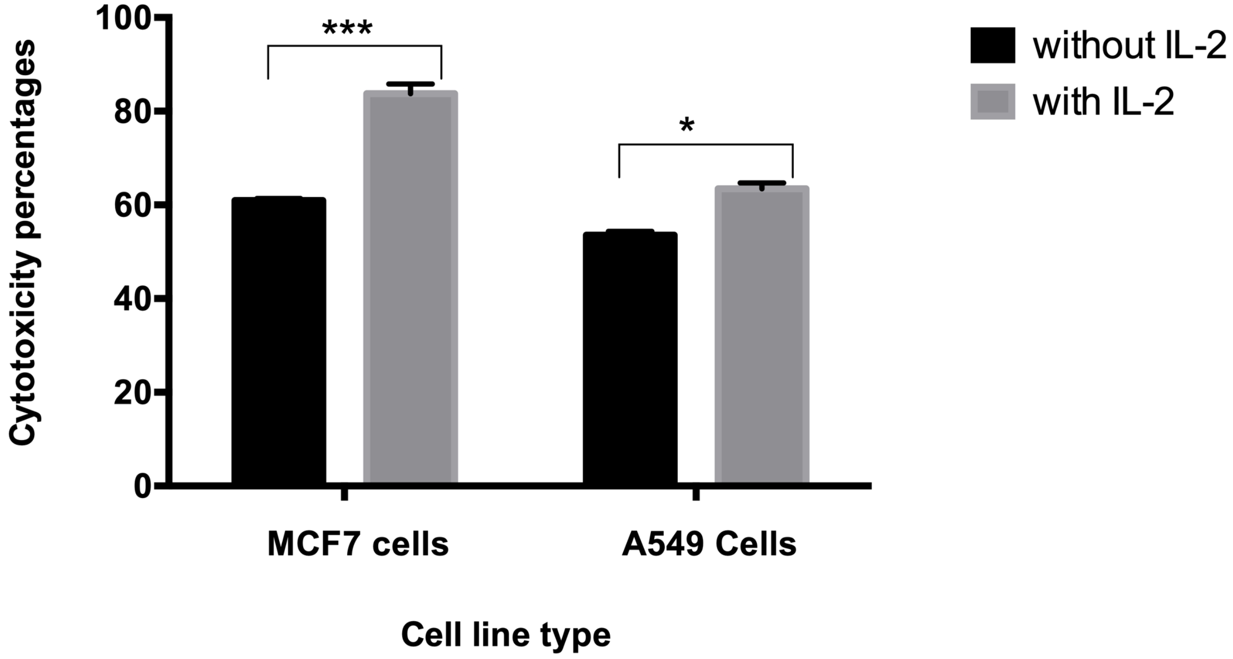

Fig 1S. Effect of IL2 on the anticancer activity of mushroom activated NK cells. The effect of pre-activated NK cells with mushroom fraction No. 1 with or without IL2 against MCF7 and A549 cells using trypan blue exclusion assay, Data are mean and s.d, ****$p < 0.0001$ and *$p < 0.037$ with one-way ANOVA with multiple comparisons (n=3).

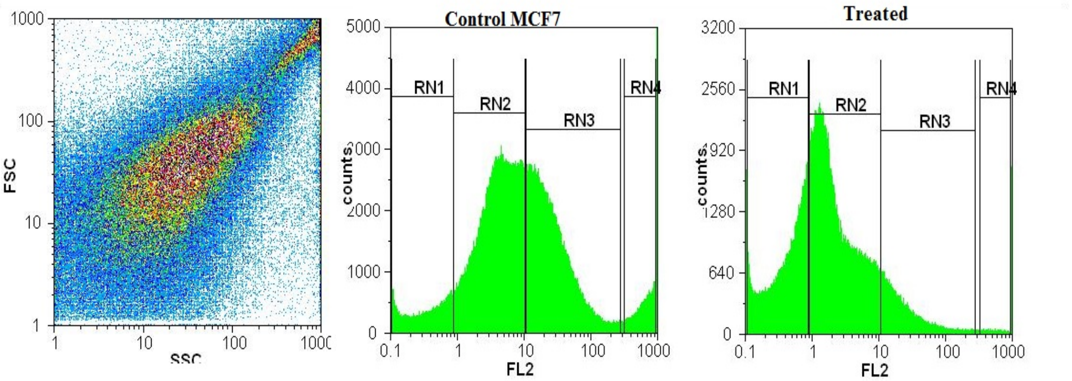

Region	Gate	Ungated	Count	Count/ml	%Gated	GMn-x	Mean-x	CV-x%
RN1	<None>	24666	24666	-	5.75	0.34	0.42	58.23
RN2	<None>	144440	144440	-	33.69	3.97	4.73	55.45
RN3	<None>	112335	112335	-	26.20	27.97	38.08	105.15
RN4	<None>	13687	13687	-	3.19	621.85	646.93	26.86

Region	Gate	Ungated	Count	Count/ml	%Gated	GMn-x	Mean-x	CV-x%
RN1	<None>	95728	95728	-	23.45	0.40	0.47	51.35
RN2	<None>	179893	179893	-	44.06	2.34	3.00	78.38
RN3	<None>	37874	37874	-	9.28	26.63	39.12	119.54
RN4	<None>	2740	2740	-	0.67	541.33	566.28	30.21

Region	Gate	Ungated	Count	Count/ml	%Gated	GMn-x	Mean-x	CV-x%
RN1	<None>	28475	28475	-	6.90	0.34	0.42	58.26
RN2	<None>	151846	151846	-	36.81	3.72	4.45	57.45
RN3	<None>	97114	97114	-	23.54	27.45	36.53	99.35
RN4	<None>	6440	6440	-	1.56	671.63	694.19	23.89

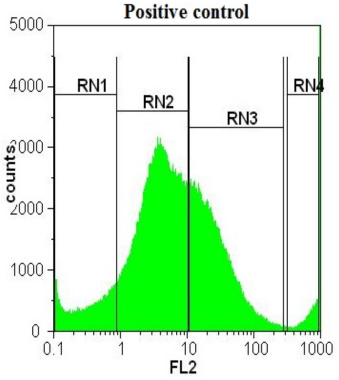

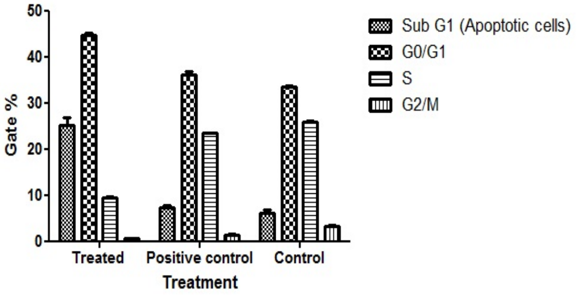

Fig 2s. Cell cycle analysis in NK-MCF7 cells coculture model.

Purified NK cells were first cultured for 3 days in the presence or absence of mushroom fractions mu1 and IL-2, NK cells from each group were then coculture with MCF7 cells (ratio of 3:1, NK cells: cancer cells) for 3 days, data are represented as mean (n=3).

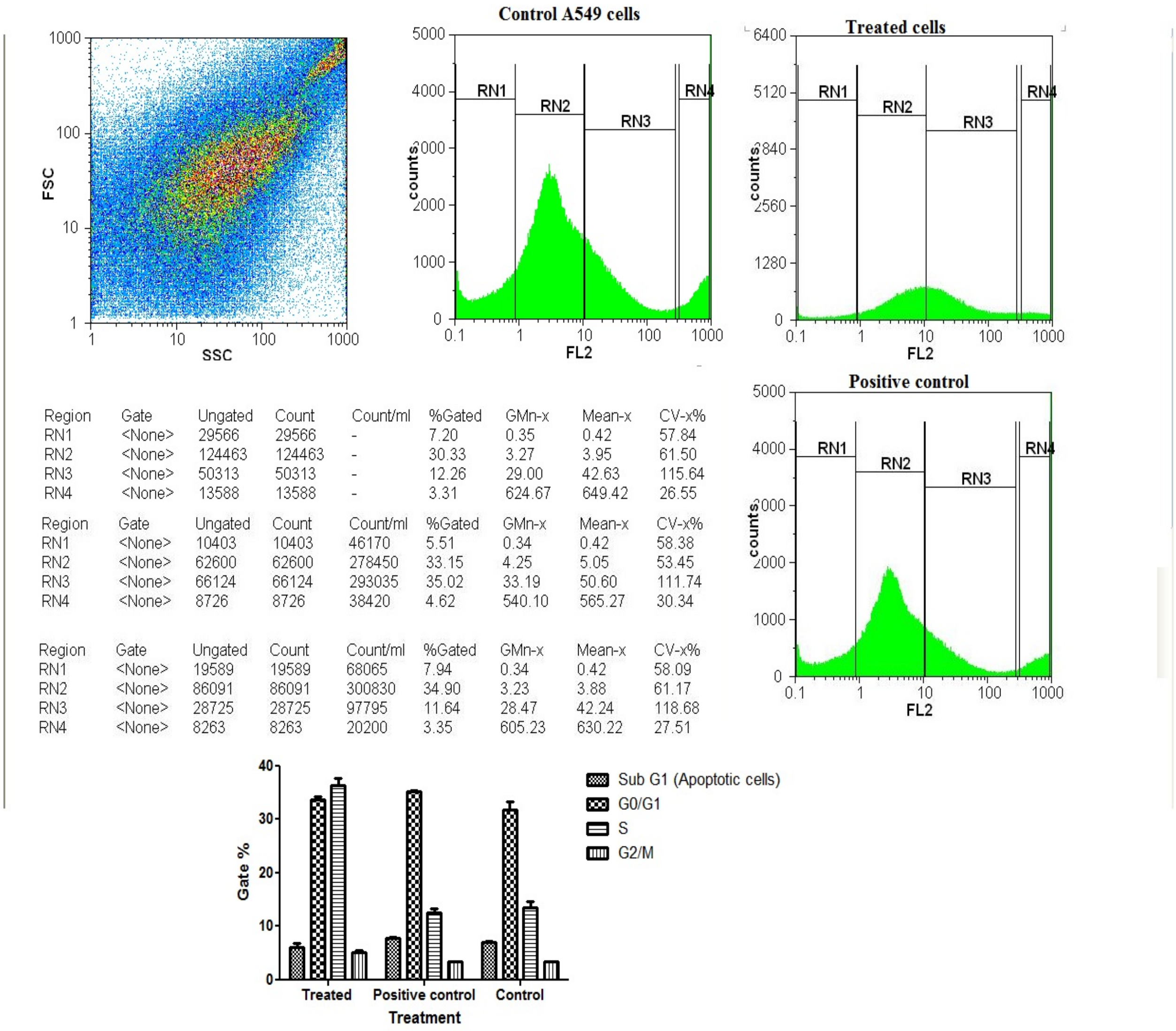

Region	Gate	Ungated	Count	Count/ml	%Gated	GMn-x	Mean-x	CV-x%
RN1	<None>	29566	29566	-	7.20	0.35	0.42	57.84
RN2	<None>	124463	124463	-	30.33	3.27	3.95	61.50
RN3	<None>	50313	50313	-	12.26	29.00	42.63	115.64
RN4	<None>	13588	13588	-	3.31	624.67	649.42	26.55

Region	Gate	Ungated	Count	Count/ml	%Gated	GMn-x	Mean-x	CV-x%
RN1	<None>	10403	10403	46170	5.51	0.34	0.42	58.38
RN2	<None>	62600	62600	278450	33.15	4.25	5.05	53.45
RN3	<None>	66124	66124	293035	35.02	33.19	50.60	111.74
RN4	<None>	8726	8726	38420	4.62	540.10	565.27	30.34

Region	Gate	Ungated	Count	Count/ml	%Gated	GMn-x	Mean-x	CV-x%
RN1	<None>	19589	19589	68065	7.94	0.34	0.42	58.09
RN2	<None>	86091	86091	300830	34.90	3.23	3.88	61.17
RN3	<None>	28725	28725	97795	11.64	28.47	42.24	118.68
RN4	<None>	8263	8263	20200	3.35	605.23	630.22	27.51

Fig 3s: Cell cycle analysis in NK-A549 cells coculture model.

Purified NK cells were first cultured for 3 days in the presence or absence of mushroom fractions mu1 and IL-2, NK cells from each group were then coculture with A549 cells (ratio of 3:1, NK cells: cancer cells) for 3 days, , data are represented as mean (n=3).

Table 7S. The estimated amounts and mole percentage of each detected monosaccharide in the dialyzed sample.

Sample	Glycosyl residue	Mass (μg)	Mol %
	Ribose (Rib)	0.3	1.3
	Arabinose (Ara)	n.d.	-
	Rhamnose (Rha)	1.4	5.7
	Fucose (Fuc)	n.d.	-
	Xylose (Xyl)	0.3	1.3
	Glucuronic Acid (GlcA)	0.8	2.8
	Galacturonic acid (GalA)	n.d.	-
	Mannose (Man)	3.3	12.0
	Galactose (Gal)	1.7	6.1
	Glucose (Glc)	18.6	67.1
	N-Acetyl Galactosamine (GalNAc)	n.d.	-
	N-Acetyl Glucosamine (GlcNAc)	1.3	3.8
	N-Acetyl Glucosamine (GlcNAc)	n.d.	-
	Σ=	27.8	

Table 8S. Relative percentage of each detected linkage in the sample.

peak	area %
Terminal Arabinofuranosyl residue (t-Araf)	0.4
Terminal Xylopyranosyl residue (t-Xyl)	0.9
Terminal Rhamnopyranosyl residue (t-Rha)	0.9
4 linked Xylopyranosyl residue (4-Xyl)	0.5
2 linked Rhamnopyranosyl (2-Rha)	1.0
Terminal Glucopyranosyl residue (t-Glc)	24.1
Terminal Mannopyranosyl residue (t-Man)	8.7
Terminal Galactopyranosyl residue (t-Gal)	2.9
2 linked Glucopyranosyl residue (2-Glc)	0.8
2 linked Mannopyranosyl residue (2-Man)	2.7
3 Linked Glucopyranosyl residue (3-Glc)	0.5
4 linked Mannopyranosyl residue (4-Man)	0.3
4 linked Glucopyranosyl residue (4-Glc)	4.3
4 linked Glucuronic Acid Residue (4-Glc A)	3.9
6 linked Glucopyranosyl residue (6-Glc)	36.0
6 linked Galactopyranosyl residue (6-Gal)	5.5
3,6 linked Mannopyranosyl residue (3,6-Man)	1.6
2,6 linked Galactopyranosyl residue (2,6-Gal)	3.0
Terminal N-acetyl Glucosamine (t-GlcNAc)	0.7
4 linked N-acetyl Glucosamine (4-GlcNAc)	1.2
